# LAPTM4B counteracts ferroptosis via suppressing the ubiquitin-proteasome degradation of SLC7A11 in non-small cell lung cancer

**DOI:** 10.1038/s41419-024-06836-x

**Published:** 2024-06-20

**Authors:** Ruyu Yan, Dan Liu, Hongjuan Guo, Minxia Liu, Dongjin Lv, Benny Björkblom, Mingsong Wu, Hongtao Yu, Hao Leng, Bingxiao Lu, Yuxiang Li, Miaomiao Gao, Tomas Blom, Kecheng Zhou

**Affiliations:** 1 School of Life Sciences, Anhui Medical University, Hefei, 230032 China; 2 Faculty of Medicine, University of Helsinki, Helsinki, 00014 Finland; 3 Department of Clinical Research, The Third Affiliated Hospital of Kunming Medical University (Tumor Hospital of Yunnan Province), Kunming, China; 4 Department of Chemistry, Umeå University, Umeå, 90187 Sweden; 5 School of Stomatology, Zunyi Medical University, Zunyi, Guizhou 563000 China; 6 Department of Medical Oncology, The Third Affiliated Hospital of Kunming Medical University (Tumor Hospital of Yunnan Province), Kunming, China; 7 Minerva Foundation Institute for Medical Research, Helsinki, 00014 Finland

**Keywords:** Cell death, Cancer metabolism

## Abstract

Non-small cell lung cancer (NSCLC) is a leading cause of cancer-related deaths worldwide, necessitating the identification of novel therapeutic targets. Lysosome Associated Protein Transmembrane 4B (LAPTM4B) is involved in biological processes critical to cancer progression, such as regulation of solute carrier transporter proteins and metabolic pathways, including mTORC1. However, the metabolic processes governed by LAPTM4B and its role in oncogenesis remain unknown. In this study, we conducted unbiased metabolomic screens to uncover the metabolic landscape regulated by LAPTM4B. We observed common metabolic changes in several knockout cell models suggesting of a role for LAPTM4B in suppressing ferroptosis. Through a series of cell-based assays and animal experiments, we demonstrate that LAPTM4B protects tumor cells from erastin-induced ferroptosis both in vitro and in vivo. Mechanistically, LAPTM4B suppresses ferroptosis by inhibiting NEDD4L/ZRANB1 mediated ubiquitination and subsequent proteasomal degradation of the cystine-glutamate antiporter SLC7A11. Furthermore, metabolomic profiling of cancer cells revealed that LAPTM4B knockout leads to a significant enrichment of ferroptosis and associated metabolic alterations. By integrating results from cellular assays, patient tissue samples, an animal model, and cancer databases, this study highlights the clinical relevance of the LAPTM4B-SLC7A11-ferroptosis signaling axis in NSCLC progression and identifies it as a potential target for the development of cancer therapeutics.

## Introduction

Non-small cell lung cancer (NSCLC) is a commonly diagnosed cancer and remains a leading cause of cancer-related deaths worldwide. Despite the availability of multiple treatment options, the survival rates for patients remain low [1]. Disordered metabolism has been identified as one of the central cancer characteristics and have emerged as potential targets for therapeutics. Within the cell, lysosomes are crucial metabolism hubs, which act as organizing centers for signal transduction that regulate cellular nutrient sensing, various metabolic processes, and metabolic adaptation [2], and lysosomal transmembrane proteins play a central role in lysosome-mediated signaling [3]. Defects in lysosome-coordinated signaling pathways are also involved in the development of a number of diseases, including cancer [4]. However, the knowledge of how lysosome-mediated cell metabolism contributes to NSCLC development is still limited.

Ferroptosis is a recently discovered form of regulated cell death characterized by increased levels of membrane lipid peroxidation [5]. The metabolic and signaling pathways regulating ferroptosis are potential therapeutic targets [6, 7], and several key molecules involved in ferroptosis regulation have been identified [8]. The antiport system Xc^−^, which consists of a SLC7A11 and SLC3A2 subunits, plays a crucial role in importing cystine into cells in exchange for exporting glutamate. Cellular cystine is further converted into cysteine [9], ultimately contributing to the synthesis of glutathione (GSH) [10]. GSH is utilized by GPX4 to counteract lipid reactive oxygen species (ROS) and malondialdehyde (MDA), the key mediators of ferroptosis [8]. Current research primarily focuses on investigating the impact of mitochondria [11], nuclei [12], and the endoplasmic reticulum [13] on ferroptosis regulation, with most molecular mechanisms being studied at the transcriptional level [12]. However, the involvement of lysosomes in ferroptosis and the post-transcriptional modifications of essential regulatory proteins in this process remains elusive.

The lysosomal transmembrane protein LAPTM4B was first cloned from hepatocellular carcinoma [14]. Serval cohort studies have reported that it is highly expressed in cancers and correlated with poor prognosis, particularly in breast cancer [15], hepatocellular carcinoma [16], acute myeloid leukemia [17], and NSCLC [18]. Functional studies have shown that LAPTM4B promotes cancer growth and metastasis [19]. Moreover, LAPTM4B stimulates cancer cell stemness [17], autophagy [20], and drug resistance [21]. Mechanistically LAPTM4B can regulate cancer progression via several pathways: LAPTM4B promotes integrin beta1 recycling and regulate cytoskeleton organization, thus enhances cell migration [22, 23]; It regulates drug resistance via the PI3K-AKT pathway [24], stimulates tumor growth via activating mTORC1 signaling [25], and regulates cell growth and autophagy though prolonging EGFR signaling [26, 27]. We have shown that LAPTM4B interacts with ceramide [28, 29] which promotes its interaction with the leucine transporter SLC3A2/SLC7A5, stimulating lysosomal leucine uptake and the downstream mTORC1 signaling [30], which is in agreement with the finding by Milkereit et al. that LAPTM4B recruits SLC3A2 into lysosome and promotes mTORC1 activity [25]. Moreover, LAPTM4B responds to cellular nutrient status [31], and regulates cellular sphingolipid and ether lipid signatures [32]. These studies suggest that LAPTM4B is vital in regulating cellular amino acid metabolism and lipid metabolism. However, the broader metabolic landscape controlled by LAPTM4B and its potential connection to oncogenesis are largely unknown.

In this study, we conducted an unbiased screen to investigate the metabolic landscape regulated by LAPTM4B. Specifically, LAPTM4B was found to impact ferroptosis, glutathione metabolism, as well as cysteine and methionine metabolism. Among these pathways, we selected ferroptosis for further investigation for the following reasons: (1) its central role in relation to other detected metabolic alterations, (2) its regulation by cellular amino acids and its effects on lipid metabolism, which is in line with previous studies on LAPTM4B [28, 30, 32], and (3) its clinical significance and potential as a therapeutic target. Through measuring the main features of ferroptosis, including lipid peroxidation, MDA, and GSH, we established that LAPTM4B suppresses ferroptosis. This finding was supported by ultrastructure analysis using transmission electron microscopy, which revealed an abundance of small, shrunk, and degenerated mitochondria in LAPTM4B-depleted NSCLC cells. Mechanistically, we demonstrate that LAPTM4B promotes the stabilization of the SLC7A11 protein by inhibiting its proteasomal degradation.

We further investigated the effects of LAPTM4B on ferroptosis using various cellular experiments and the nude mice xenograft model. Our results showed that LAPTM4B counteracts erastin-induced ferroptosis in vitro and tumor growth in vivo. Importantly, we observed a correlation between the expression levels of LAPTM4B and SLC7A11 in tissue samples from nude mice and NSCLC patients. Additionally, data analysis of The Cancer Genome Atlas (TCGA) revealed that high expression of LAPTM4B and SLC7A11 associated with poor prognosis in NSCLC patients. To generalize the potential impact of our findings, we profiled LAPTM4B regulation of the soluble metabolome in three cancer cell lines. Interestingly, the metabolomics profiling revealed a high enrichment of ferroptosis and related metabolic alterations.

In summary, our study uncovers the metabolic landscape regulated by LAPTM4B. We demonstrate that LAPTM4B counteracts ferroptosis via suppressing the NEDD4L/ZRANB1 mediated proteasomal degradation of SLC7A11. This signaling pathway promotes the progression of NSCLC and potentially other cancers, making it a promising target for the development of cancer therapeutics.

## Results

### Metabolomics profiling depicts the metabolic landscape regulated by LAPTM4B

**Fig. 1:**
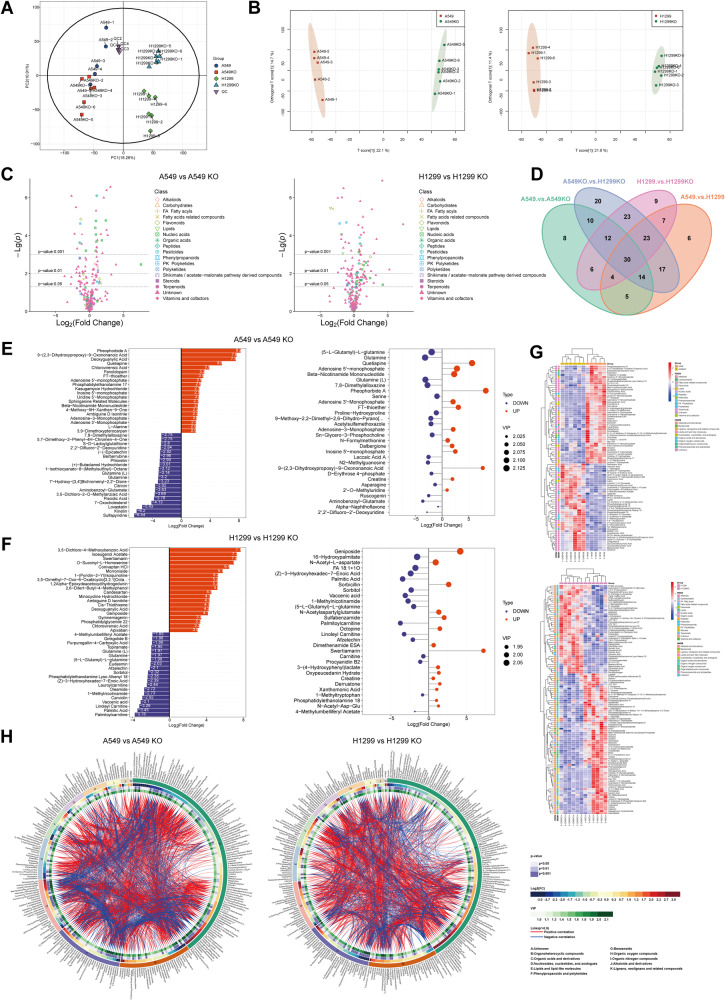
Metabolomics profiling reveals the metabolic landscape regulated by LAPTM4B. **A** Principal component analysis (PCA) of metabolomics profiling results in wild-type (WT) and LAPTM4B knockout (KO) A549 cells, as well as in WT and LAPTM4B KO H1299 cells, based on negative mode (NEG) data. The plot shows replicates from 5–6 independent experiments. **B** Orthogonal partial least squares-discriminant analysis (OPLS-DA) plot depicting metabolites perturbed by LAPTM4B depletion in A549 (left panel) and H1299 cells (right panel), based on NEG data. **C** Volcano plots displaying significantly dysregulated metabolites in A549 cells (left panel) and H1299 cells (right panel) after applying the threshold (Fold Change > 1.5 or Fold Change < 0.667, *p* < 0.05, and VIP > 1). The log_2_(Fold Change) and −Lg(*p*) values of the significantly altered metabolites are shown. **D** Intersection analysis of altered metabolites in WT and LAPTM4B KO cells. A total of 52 metabolites were commonly enriched in LAPTM4B KO samples from both cell lines, based on NEG data. **E** Overview of enriched metabolites in WT and LAPTM4B KO A549 cells. Left panel: Top 20 upregulated (red) and top 20 downregulated (blue) metabolites based on fold change. Right panel: Top 30 dysregulated metabolites based on VIP values and fold change. **F** Overview of enriched metabolites in WT and LAPTM4B KO H1299 cells. Left panel: Top 20 upregulated (red) and top 20 downregulated (blue) metabolites based on fold change. Right panel: Top 30 dysregulated metabolites based on VIP values and fold change. **G** Heatmap depicting the altered metabolites in WT and KO A549 cells (upper panel) and in WT and KO H1299 cells (lower panel), based on NEG data. “Red” indicates upregulation, while “blue” indicates downregulation. **H** Circos plot displaying the associations between metabolites involved in different cellular metabolic processes. The plot includes the top 300 metabolites with the highest VIP values.

Previous studies conducted by us and others have demonstrated that LAPTM4B enhances lysosomal leucine uptake and influences cellular lipid signatures [25, 30, 32], indicating a role in amino acid and lipid metabolism. Additionally, a main interaction partner of LAPTM4B is SLC3A2, a subunit of heterodimeric amino acid transport complexes with central roles in regulating cellular nutrient signaling [25, 30]. Moreover, LAPTM4B expression is affected by cellular nutrient statue [31]. These findings led us to hypothesize that LAPTM4B may play a wider role in cellular metabolic processes. To gain further insight into the metabolic landscape regulated by LAPTM4B in NSCLC, we conducted metabolomics analysis. We knocked out LAPTM4B from A549 and H1299 cell lines using CRISPR-Cas9, and verified the cell lines through immunoblotting and Sanger sequencing (Supplementary Fig. S1A, B). Six biological replicate samples from each cell line were prepared and harvested for LC-MS metabolomics profiling. In our analysis, we successfully detected more than 732 metabolites in both wild-type (WT) and LAPTM4B knock-out (KO) A549 cells, as well as 733 metabolites in WT and LAPTM4B KO H1299 cells. Principal Component Analysis (PCA) plots demonstrated that all individual samples were clustered within their respective replicates, with clear separation between WT and LAPTM4B KO samples in both A549 and H1299 cells (Fig. 1A, Supplementary Fig. S1C). The orthogonal partial least squares-discriminant analysis (OPLS-DA) plot clearly separated tested samples into two blocks according to their metabolic profiles of different groups in both positive (POS) and negative (NEG) ion modes, indicating that the metabolites in both A549 and H1299 were significantly perturbed by LAPTM4B depletion (Fig. 1B, Supplementary Fig. S1D).

We then performed feature detection, alignment, and threshold selection (Fold Change > 1.5 or Fold Change < 0.667, *p* < 0.05, and VIP > 1), the altered metabolites with classification were visualized by volcano plot (Fig. 1C). We next employed Venny 2.1 (https://bioinfogp.cnb.csic.es/tools/venny/) diagram to identify the common metabolites regulated by LAPTM4B. To this end, 281 metabolites were found to be significantly altered by LAPTM4B in A549 cells (*p* < 0.05, and VIP > 1), while 326 metabolites showed significant changes in H1299 cells (Supplementary Table S1). Among these, 52 metabolites were commonly enriched in LAPTM4B KO samples from both cell lines when analyzing the NEG dataset (Fig. 1D), and 84 metabolites when analyzing the POS dataset (Supplementary Fig. S1E, Supplementary Table S2). An overview of the enriched metabolites revealed the identification of Glutathione, Glutamine, and Cysteine, which are crucial metabolites in ferroptosis (Fig. 1E, F). The heatmap displayed the significantly altered metabolites in A549 and H1299 cells (Fig. 1G, Supplementary Fig. S1F). We further selected the metabolites with top 300 VIP values and the Circos plots display the association between LAPTM4B regulated metabolites, classification, log_2_(Fold Change), as well as *p* value and VIP (Fig. 1H). Herein, our unbiased metabolomics analysis revealed the metabolic landscape regulated by LAPTM4B, and noted a number of metabolites related with ferroptosis interfered upon the depletion of LAPTM4B.

### LAPTM4B suppresses ferroptosis in NSCLC

**Fig. 2:**
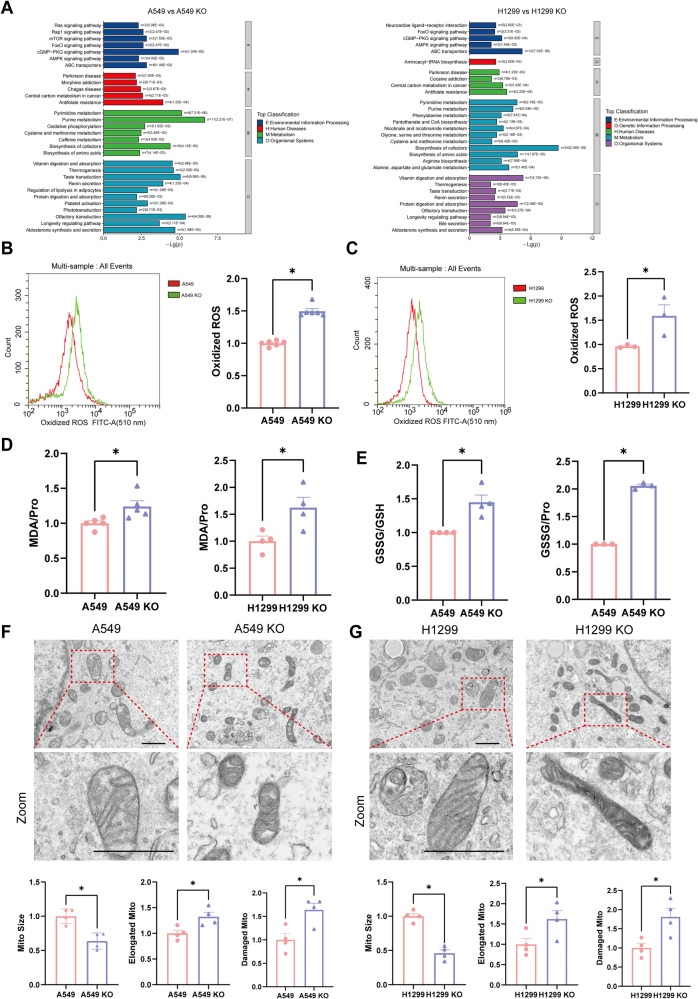
LAPTM4B suppresses ferroptosis in NSCLC. **A** KEGG analysis of the top 30 pathways regulated by LAPTM4B. Left Panel: Analysis from wild-type (WT) and LAPTM4B knockout (KO) A549 cells. Right Panel: Analysis from WT and LAPTM4B KO H1299 cells. **B** Flow cytometry analysis of lipid peroxidation using a fluorescence-based reporter BODIPY® 581/591 C11. Left panel: Representative experiment showing oxidized ROS levels in A549 and A549 KO cells. Right panel: Quantification of oxidized ROS levels from three independent experiments, presented as mean ± SEM. Data normalized to “A549”. *p*(A549, A549 KO) = 0.0000001. **C** Flow cytometry analysis of lipid peroxidation using a fluorescence-based reporter BODIPY® 581/591 C11. Left panel: Representative experiment showing oxidized ROS levels in H1299 and H1299 KO cells. Right panel: Quantification of oxidized ROS levels from three independent experiments, presented as mean ± SEM. Data normalized to “H1299”. *p*(H1299, H1299 KO) = 0.0263. **D** Measurement of malondialdehyde (MDA) levels in WT and KO A549 cells, as well as in WT and KO H1299 cells. Quantification of MDA levels from four independent experiments, presented as mean ± SEM. *p*(A549, A549 KO) = 0.0336. *p*(H1299, H1299 KO) = 0.027. **E** Measurement of the glutathione disulfide (GSSG)/glutathione (GSH) ratio and the amount of GSSG normalized to total protein in WT and KO A549 cells. Quantification of GSSG/GSH ratio and GSSG/Protein levels from three independent experiments, presented as mean ± SEM. For GSSG/GSH, *p*(A549, A549 KO) = 0.0029. For GSSG/Pro, *p*(A549, A549 KO) = 2.68E−06. **F** Transmission electron microscopy analysis of mitochondria ultrastructure in WT and KO A549 cells. Upper panel: Representative images showing mitochondria morphology. Lower panel: Quantification of mitochondria size, elongated mitochondria, and damaged mitochondria from four independent experiments, presented as mean ± SEM. Data normalized to “A549”. For mitochondria size, *p*(A549, A549 KO) = 0.0021. For elongated mitochondria, *p*(A549, A549 KO) = 0.0131. For damaged mitochondria, *p*(A549, A549 KO) = 0.0078. Scale bar: 1 µm. The region in the dashed red box is amplified in the lower panel. **G** Transmission electron microscopy analysis of mitochondria ultrastructure in WT and KO H1299 cells. Upper panel: Representative images showing mitochondria morphology. Lower panel: Quantification of mitochondria size, elongated mitochondria, and damaged mitochondria from four independent experiments, presented as mean ± SEM. Data normalized to “H1299”. For mitochondria size, *p*(H1299, H1299 KO) = 0.0001. For elongated mitochondria, *p*(H1299, H1299 KO) = 0.0247. For damaged mitochondria, *p*(H1299, H1299 KO) = 0.0133. Scale bar: 1 µm. The region in the dashed red box is amplified in the lower panel.

We then conducted pathway enrichment of the altered metabolites after threshold selection (*p* < 0.05, and VIP > 1). KEGG analysis of the top 30 pathways indicated that LAPTM4B regulates ABC transporters, AMPK signaling pathway, Central carbon metabolism in cancer, and Cysteine and methionine metabolism, which are known to be central in cancer development (Fig. 2A). The Sankey diagrams based on the top 15 enriched pathways further predicts the flows from key metabolites to the biological pathways, suggesting that LAPTM4B regulated metabolic pathways associate with several diseases, including cancer (Supplementary Fig. S2A).

Among the various cellular metabolic processes analyzed, Cysteine and methionine metabolism, Purine metabolism, and AMPK signaling pathway emerged as top candidates (Fig. 2A), all of which play central roles in ferroptosis [8]. Notably, ferroptosis was found to be the most enriched metabolic process regulated by LAPTM4B in H1299 cells when analyzing the data in POS (positive ion)-KEGG pathway (Supplementary Fig. S2B-C). Based on these findings, we hypothesized that LAPTM4B is capable of modulating ferroptosis in NSCLC. Ferroptotic cells display elevated levels of lipid peroxidation and MDA, along with dysregulated GSH, and are morphologically characterized by dysmorphic small mitochondria with shrunken cristae [8]. To investigate whether LAPTM4B indeed regulates ferroptosis, we measured the lipid peroxidation using a fluorescence-based reporter BODIPY® 581/591 C11. Our experimental data demonstrated that the lipid peroxidation levels were increased 49.6–59% in LAPTM4B-depleted cells. These results were consistent in both A549 and H1299 cells, suggesting an essential role of LAPTM4B in controlling cellular oxidative stress (Fig. 2B, C). ROS-induced damage to DNA, proteins, and lipids are associated with several biological processes, including mitochondrial electron transport chain dysfunction, lipid oxidation, and ferroptosis [33]. Next, we assessed the level of MDA, a characteristic marker of ferroptosis and a secondary product of lipid peroxidation. Interestingly, MDA levels significantly increased in LAPTM4B KO H1299 cells (approximately 62.4%) and in LAPTM4B KO A549 cells (approximately 24.2%), indicating that LAPTM4B is capable of suppressing lipid peroxidation in NSCLC (Fig. 2D). To further validate our hypothesis, we measured the oxidated form (GSSG) and reduced form (GSH) of glutathione, another recognized biomarker of ferroptosis. Indeed, both the ratio of GSSG/GSH and total GSSG (normalized to total protein) were increased upon LAPTM4B KO (Fig. 2E). We next examined the ultrastructure of mitochondria using transmission electron microscope and observed a notable abundance of small mitochondria with shrunken cristae in LAPTM4B KO cells (Fig. 2F, G), particularly evident in LAPTM4B KO H1299 cells (Fig. 2G). Further quantification revealed a 36.2% reduction in mitochondria size in LAPTM4B KO A549 cells and a 63.9% reduction in LAPTM4B KO H1299 cells. Moreover, the elongated mitochondria increased about 32.4% in LAPTM4B KO A549 cells, and 63.9% in LAPTM4B KO H1299 cells. Accordingly, the damaged mitochondria increased about 63.9-81% upon LAPTM4B depletion, with visibly swollen and degenerated mitochondria (Fig. 2F, G, Supplementary Fig. S2D). Iron metabolism-associated proteins, such as TFRC, FTH1, FTL, SLC11A2 and SLC40A1, are crucial for ferroptosis defense [34]. Interestingly, both the transcript levels (Supplementary Fig. S3A) and protein levels (Supplementary Fig. S3B, C) of these iron metabolism-associated proteins remain unchanged in LAPTM4B depleted cells, suggesting other mechanisms are involved. These biochemical data, combined with the ultrastructural analysis of mitochondrial morphology, establish that LAPTM4B suppresses ferroptosis in NSCLC.

### LAPTM4B suppresses the ubiquitination-proteasome degradation of SLC7A11

**Fig. 3:**
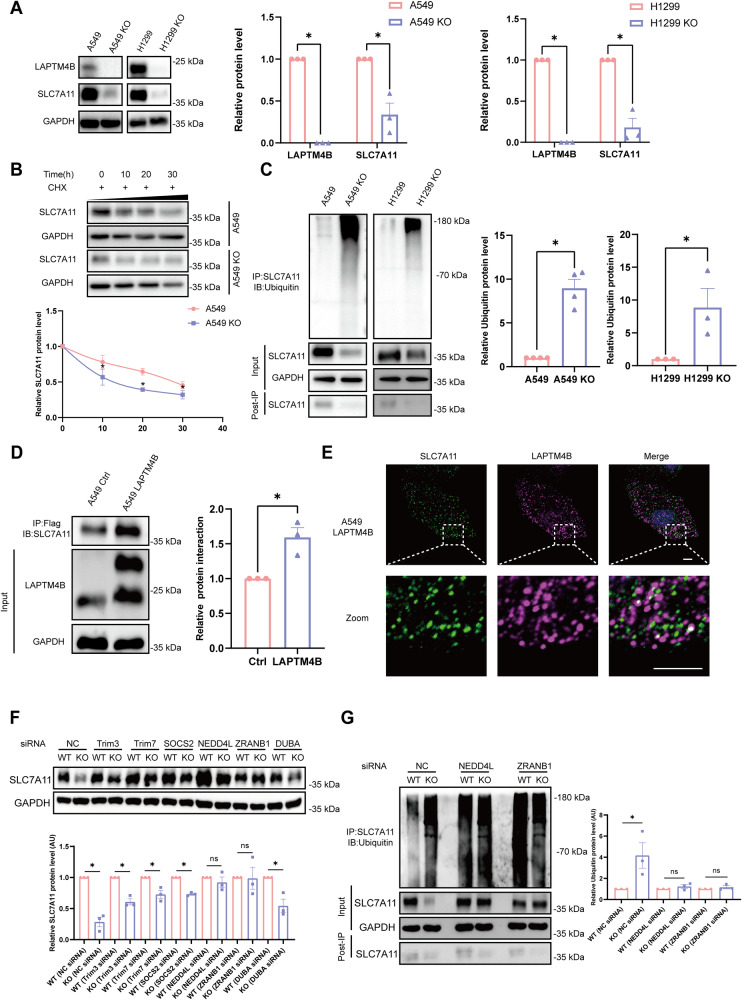
LAPTM4B suppresses the ubiquitination-proteasome degradation of SLC7A11. **A** Western blot analysis of SLC7A11 and LAPTM4B protein levels in LAPTM4B-depleted A549 and H1299 cells. Left panel: Representative experiment. Right panel: Quantification of *n* = 3 experiments, presented as mean ± SEM, data normalized to “A549” or “H1299”. For SLC7A11 levels, *p*(A549, A549 KO) = 0.0041, *p*(H1299, H1299 KO) = 0.0009. For LAPTM4B levels, *p*(A549, A549 KO) = 4.24E−13, *p*(H1299, H1299 KO) = 5.83E−18. **B** Western blot analysis of SLC7A11 protein levels in WT and LAPTM4B KO A549 cells treated with 50 µg/mL CHX for the indicated times. Upper panel: Representative experiment. Lower panel: Quantification of n = 3 experiments, presented as mean ± SEM. *p < 0.05. **C** Immunoprecipitation followed by immunoblotting with SLC7A11 antibody and Ubiquitin antibody in WT and LAPTM4B KO A549 cells, as well as WT and LAPTM4B KO H1299 cells. Left panel: Representative experiment. Right panel: Quantification of at least three experiments, presented as mean ± SEM, data normalized to “A549” or “H1299”. *p*(A549, A549 KO) = 0.0001, *p*(H1299, H1299 KO) = 0.0266. **D** Immunoprecipitation of Flag-tagged LAPTM4B stably expressing A549 cells and control cells using Flag antibody, followed by immunoblotting with SLC7A11 antibody. Left panel: Representative experiment. Right panel: Quantification of *n* = 3 experiments, presented as mean ± SEM, data normalized to “Ctrl”. *p*(Ctrl, LAPTM4B) = 0.0127. **E** Immunofluorescence staining of Flag-tagged LAPTM4B stably expressing cells using anti-LAPTM4B antibody (magenta) and anti-SLC7A11 antibody (green). Scale bar: 5 µm. The region in the dashed white box is amplified in the lower panel. **F** WT and LAPTM4B KO A549 cells were transfected with indicated siRNA, and subsequent western blotting was performed to determine SLC7A11 protein levels. Upper panel: Representative experiment. Lower panel: Quantification of *n* = 3 experiments, presented as mean ± SEM. *p < 0.05. **G** WT and LAPTM4B KO A549 cells were transfected with indicated siRNA. Immunoprecipitation of the cell lysate using SLC7A11 antibody, followed by immunoblotting with Ubiquitin antibody. Left panel: Representative experiment. Right panel: Quantification of at least three experiments, presented as mean ± SEM. *p < 0.05.

The cellular GSH is generated through the coordinated action of system Xc^−^ (SLC7A11/SLC3A2) and GPX4 [8], previous studies have shown that LAPTM4B directly interacts with the leucine transporter (SLC7A5/SLC3A2) and stimulates downstream mTORC1 signaling [25, 30]. Herein, in this study we questioned whether LAPTM4B’s ferroptosis-suppressive function is dependent on SLC7A11 or GPX4. Interestingly, the protein level of SLC7A11 significantly decreased in LAPTM4B KO cells, while the GPX4 level showed a tendency for increasing levels (Fig. 3A, Supplementary Fig. S4A). This observation piqued our interest in understanding how LAPTM4B regulates SLC7A11. qPCR experiments revealed no difference in SLC7A11 transcript levels upon LAPTM4B depletion (Supplementary Fig. S4B), leading us to suspect that LAPTM4B may instead regulate stability of SLC7A11 protein. To validate this hypothesis, we treated cells with the protein synthesis inhibitor cycloheximide and performed western blotting to measure the protein levels. Our data indicated that SLC7A11 has a half-life of approximately 10 hours (Supplementary Fig. S4C), consistent with previous studies in HepG2 cells [35]. To elucidate which pathway is responsible for SLC7A11 protein degradation, we additionally treated cells with the V-ATPase inhibitor bafilomycin-A1 to suppress lysosomal degradation or MG-132 to block proteasome activity, in combination with cycloheximide treatment. Our data showed that MG-132 treatment prominently diminished the degradation of SLC7A11, while bafilomycin-A1 did not exhibit any significant regulatory effect (Supplementary Fig. S4D). These findings suggest that the proteasomal pathway plays a major role in the turnover of SLC7A11. Next, we sought to determine whether LAPTM4B regulates the degradation of SLC7A11 protein. We measured the level of SLC7A11 in cycloheximide-treated WT and LAPTM4B KO cells and observed that LAPTM4B significantly attenuated the degradation of SLC7A11 (Fig. 3B). Poly-ubiquitination is the central feature of proteasome degradation, and we thus investigated the poly-ubiquitination level of SLC7A11 via immunoprecipitation with an SLC7A11 antibody in MG-132-treated cells, followed by western blotting to measure the ubiquitin level. As anticipated, the poly-ubiquitination level of SLC7A11 was substantially enhanced in LAPTM4B KO cells (approximately 9-folds) compared to WT A549 cells. Similar results were obtained in H1299 cells (approximately 8.5-folds) (Fig. 3C), indicating that LAPTM4B suppressive role on the proteasomal degradation of SLC7A11 is not cell-specific. Furthermore, we investigated whether these regulations were mediated by a direct protein-protein interaction. To this end, we generated stable LAPTM4B-expressing A549 and H1299 cells using a lentivirus system with approximately 3–5 folds increased LAPTM4B protein levels over WT protein (Supplementary Fig. S4E). Immunoprecipitation showed that LAPTM4B is indeed capable of interacting with the SLC7A11 protein (Fig. 3D), which was in line with immunofluorescent experiment revealing the partial co-localization between LAPTM4B and SLC7A11 (Fig. 3E).

Next, we investigated the regulatory interplay between LAPTM4B and the ubiquitin-mediated degradation of SLC7A11. Herein, we initially surveyed the literature and identified five well-established ubiquitin ligases pivotal in the degradation process of SLC7A11, namely Trim3 [36], Trim7 [37], SOCS2 [35], NEDD4L [38] and ZRANB1 [39]. Additionally, one deubiquitinase, DUBA [40], was also selected for further examination. Subsequently, a siRNA screening approach (Supplementary Fig. S4F) was employed to pinpoint the key ubiquitin ligases or deubiquitinases involved in LAPTM4B-mediated regulation of SLC7A11 degradation. Intriguingly, in LAPTM4B knockout cells, as compared to wild-type cells, it was observed that silencing of either NEDD4L or ZRANB1 effectively abrogated the differential levels of SLC7A11, while silencing the remaining four genes failed to elicit a similar effect (Fig. 3F). Subsequent ubiquitination assays revealed that knockdown of NEDD4L/ZRANB1 nullified the differential ubiquitination levels of SLC7A11 induced by LAPTM4B depletion (Fig. 3G). These results were obtained in both A549 and H1299 cells (Supplementary Fig. S4G, H), underscoring the role of the ubiquitin ligase NEDD4L/ZRANB1 in mediating LAPTM4B’s regulation of SLC7A11 ubiquitination degradation.

Collectively, these data indicate that LAPTM4B interacts with SLC7A11 and suppresses the NEDD4L/ZRANB1 mediated ubiquitination and proteasome degradation, which could represent a novel mechanism of ferroptosis regulation.

### Depletion of LAPTM4B accelerates erastin-induced ferroptosis

**Fig. 4:**
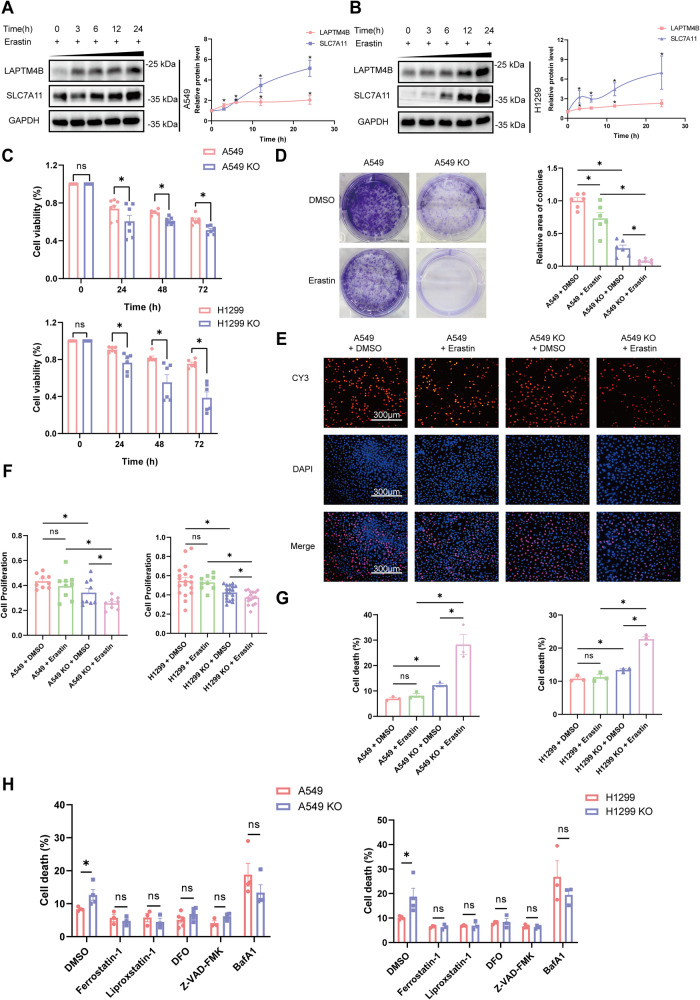
Depletion of LAPTM4B accelerates erastin-induced ferroptosis. **A** Western blot analysis of LAPTM4B and SLC7A11 protein levels in A549 cells treated with 5 µM erastin for the indicated times. Left panel: Representative experiment. Right panel: Quantification of *n* = 3 experiments, presented as mean ± SEM. *p < 0.05. **B** Western blot analysis of LAPTM4B and SLC7A11 protein levels in H1299 cells treated with 5 µM erastin for the indicated times. Left panel: Representative experiment. Right panel: Quantification of *n* = 3 experiments, presented as mean ± SEM. *p < 0.05. **C** Cell viability measured by the Cell Counting Kit-8 (CCK8) assay in WT and LAPTM4B depleted A549 cells (Up Panel), and WT and LAPTM4B KO H1299 cells (Down Panel) incubated with 5 µM erastin for 0 h, 24 h, 48 h, and 72 h. Quantification of at least five experiments, presented as mean ± SEM. *p*(A549, A549 KO)_24h = 0.0477, *p*(A549, A549 KO)_48h = 9.927E−05, *p*(A549, A549 KO)_72h = 0.0016, *p*(H1299, H1299 KO)_24h = 0.0048, *p*(H1299, H1299 KO)_48h = 0.0059, *p*(H1299, H1299 KO)_72h = 0.0001. **D** 4 × 10^3^ WT or LAPTM4B KO A549 cells were seeded into a 6-well plate, treated with 5 μM erastin for 24 h and cultured at 37 °C for 10 days. Afterwards, the cells were fixed with methanol, stained with crystal violet, and subsequently imaged and quantified. Left panel: representative experiment. Right panel: quantification of *n* = 3 experiments, mean ± SEM. *p*(A549_DMSO, A549_Erastin) = 0.0115, *p*(A549_DMSO, A549 KO_DMSO) = 3.644E-07, *p*(A549 KO_DMSO, A549 KO_Erastin) = 0.0015, *p*(A549_Erastin, A549 KO_Erastin) = 1.339E−05. **E** 8 × 10^3^ WT or LAPTM4B KO A549 cells were seeded into 96-well plates. Following treatment with 5 μM erastin for 24 h, the cells were stained with DAPI (blue) and EdU (red) to visualize the proliferative cells. Representative experiments shown. **F** Quantification results of Edu experiments in WT and LAPTM4B KO A549 cells (Left panel), as well as in WT and LAPTM4B KO H1299 cells (Right panel). Quantification of *n* = 5 experiments, mean ± SEM. *p*(A549_DMSO, A549 KO_DMSO) = 0.0158, *p*(A549 KO_DMSO, A549 KO_Erastin)=0.0371, *p*(A549_Erastin, A549 KO_Erastin) = 0.0011, *p*(H1299_DMSO, H1299 KO_DMSO) = 0.0032, *p*(H1299 KO_DMSO, H1299 KO_Erastin) = 0.0176, *p*(H1299 _Erastin, H1299 KO_Erastin) = 9.69E−06. **G** Cell death was measured by PI assay in WT and LAPTM4B KO A549 cells (Left panel), as well as in WT and LAPTM4B KO H1299 cells (Right panel). Cells were treated with 5 μM erastin for 24 h. Quantification of *n* = 3 experiments, mean ± SEM. *p*(A549_DMSO, A549 KO_DMSO) = 0.0017, *p*(A549 KO_DMSO, A549 KO_Erastin)=0.0081, *p*(A549_Erastin, A549 KO_Erastin)=0.0221, *p*(H1299_DMSO, H1299 KO_DMSO) = 0.0006, *p*(H1299 KO_DMSO, H1299 KO_Erastin) = 0.0003, *p*(H1299_Erastin, H1299 KO_Erastin) = 0.0003. **H** Cell death was measured by PI assay in WT and LAPTM4B KO A549 cells (Left panel), as well as WT and LAPTM4B KO H1299 cells (Right panel). Cells were treated with 1 μM Ferrostatin-1, 1 μM Liproxstatin-1, 50 μM Deferoxamine (DFO), 10 μM Z-VAD-FMK, or 5 μM Bafilomycin A1 (BafA1) for 24 h. Quantification of *n* = 3 experiments, mean ± SEM. *p*(A549_DMSO, A549 KO_DMSO) = 0.0216, *p*(H1299_DMSO, H1299 KO_DMSO) = 0.0241.

LAPTM4B appears to inhibit ferroptosis by suppressing the degradation of SLC7A11. Erastin is a well-known ferroptosis inducer which blocks the transport activity of system Xc^−^ [5], we next examine whether LAPTM4B protects from erastin-induced ferroptosis. Interestingly, in both A549 and H1299 cells, the protein level of LAPTM4B significantly increased in a time-dependent manner upon erastin treatment, suggesting a potential protective function of LAPTM4B in erastin-induced ferroptosis (Fig. 4A, B). Notably, we also observed an increase in SLC7A11 levels upon erastin treatment (Fig. 4A, B), which is in line with previous findings [41]. To further elaborate this concept, we measured cell viability to evaluate the cellular effects of erastin-induced ferroptosis. The Cell Counting Kit-8 (CCK8) assay demonstrated that erastin treatment resulted in a more pronounced reduction of cell viability in LAPTM4B KO cells compared to WT cells in both A549 and H1299 cells (Fig. 4C). Furthermore, we performed colony formation experiments to assess cell proliferative capability in the longer term (~2 weeks) and found that LAPTM4B KO cells formed less area of colonies upon erastin treatment, compared to WT cells (Fig. 4D, Supplementary Fig. S5A). Additionally, we employed EdU staining to quantify cell proliferation in live cells using fluorescent microscopy. These experiments revealed a more pronounced drop of proliferative cell percentage induced by erastin treatment specifically in LAPTM4B KO cells, compared to that observed in WT cells (Fig. 4E, F, Supplementary Fig. S5B). Thereafter, we employed PI assay to characterize cell death. Our findings reveal that LAPTM4B-deficient cells exhibit heightened susceptibility to erastin-induced cell death (Fig. 4G), consistent with the results observed through EdU staining. Furthermore, treatment with both ferroptosis inhibitors (Ferrostatin-1, Liproxstatin-1) and iron ion chelating agent (DFO) resulted in a reduction in the percentage of cell death and the mitigation of the differences induced by LAPTM4B depletion (Fig. 4H).

Previous studies indicate LAPTM4B inhibits apoptosis [18, 42] and promotes autophagy [20], our experiments from A549 and H1299 cells reported upregulated apoptotic cell rate (Supplementary Fig. S5C) and reduced LC3-II level (Supplementary Fig. S5D) in LAPTM4B knockout cells, suggesting LAPTM4B still regulates apoptosis and autophagy in NSCLC. To ascertain whether LAPTM4B-mediated regulation of ferroptosis represents the predominant mechanism of cell death in NSCLC, we subjected cells to inhibitors targeting apoptosis or autophagy, subsequently quantifying cell death through PI assay. Our results suggest that both ferroptosis and apoptosis constitute the major form of cell death regulated by LAPTM4B in NSCLC (Fig. 4H).

Collectively, the data from the CCK8 assay, colony formation experiment, EdU staining, and PI assay indicate that LAPTM4B depletion enhances erastin-induced ferroptosis.

### Overexpression of LAPTM4B counteracts ferroptosis in NSCLC via promoting the stability of SLC7A11

**Fig. 5:**
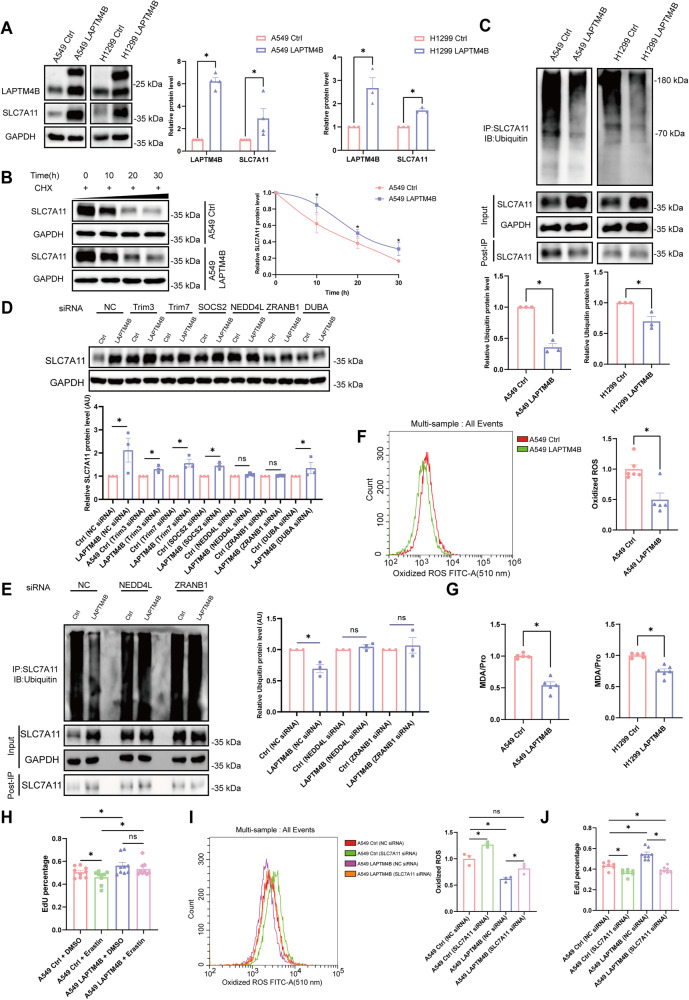
Overexpression of LAPTM4B enhances SLC7A11 stability and protects against ferroptosis in NSCLC. **A** Protein levels of SLC7A11 in LAPTM4B stably expressing A549 and H1299 cells were assessed by Western blotting. Left panel: Representative experiment. Right panel: Quantification of *n* = 3 experiments, mean ± SEM, data normalized to “A549 Ctrl” or “H1299 Ctrl”. For SLC7A11 levels, *p*(A549 Ctrl, A549 LAPTM4B) = 0.0359, *p*(H1299 Ctrl, H1299 LAPTM4B) = 0.0003. For LAPTM4B levels, *p*(A549 Ctrl, A549 LAPTM4B) = 2.067E−06, *p*(H1299 Ctrl, H1299 LAPTM4B) = 0.009. **B** A549 cells stably expressing LAPTM4B and control cells were treated with 50 µg/mL CHX for the indicated times, and SLC7A11 protein levels were assessed by Western blotting. Upper panel: Representative experiment. Lower panel: Quantification of *n* = 3 experiments, mean ± SEM. * *p* < 0.05. **C** LAPTM4B stably expressing A549 and H1299 cells, as well as control cells, were subjected to immunoprecipitation. Cells were treated with 20 µmol/L MG-132 for 12 h before harvesting. Immunoprecipitation was performed with SLC7A11 antibody, and the lysates were immunoblotted with an antibody against Ubiquitin. Upper panel: Representative experiment. Lower panel: Quantification of *n* = 3 experiments, mean ± SEM, data normalized to “A549 Ctrl” or “H1299 Ctrl”. *p*(A549 Ctrl, A549 LAPTM4B) = 0.00012, *p*(H1299 Ctrl, H1299 LAPTM4B) = 0.0099. **D** Stably expressing LAPTM4B A549 cells and the control cells were transfected with the indicated siRNA, and subsequent western blotting was performed to determine SLC7A11 protein levels. Upper panel: Representative experiment. Lower panel: Quantification of *n* = 3 experiments, presented as mean ± SEM. * *p* < 0.05. **E** Stably expressing LAPTM4B A549 cells and the control cells were transfected with indicated siRNA. Immunoprecipitation of the cell lysate using SLC7A11 antibody, followed by immunoblotting with Ubiquitin antibody. Left panel: Representative experiment. Right panel: Quantification of at least three experiments, presented as mean ± SEM. * *p* < 0.05. **F** LAPTM4B stably expressing A549 cells and control cells were harvested to measure lipid peroxidation. Left panel: Representative experiment. Right panel: Quantification of *n* = 3 experiments, mean ± SEM. *p*(A549 Ctrl, A549 LAPTM4B) = 0.0016. **G** LAPTM4B stably expressing A549 and H1299 cells, as well as control cells, were harvested to measure MDA. Quantification of *n* = 3 experiments, mean ± SEM. *p*(A549 Ctrl, A549 LAPTM4B) = 0.0002, *p*(H1299 Ctrl, H1299 LAPTM4B) = 0.0003. **H** LAPTM4B overexpressing A549 cells and control cells were seeded at a density of 8 × 10^3^ cells per well in 96-well plates. After treatment with 5 μM erastin for 24 h, cells were stained with DAPI (blue) and EdU (red) to visualize proliferative cells. Quantification data from *n* = 3 experiments, presented as mean ± SEM. Statistical analysis revealed *p*(A549 Ctrl_DMSO, A549 Ctrl_Erastin) = 0.0481 and *p*(A549 Ctrl_DMSO, A549 LAPTM4B_DMSO) = 0.0467, *p*(A549 Ctrl_Erastin, A549 LAPTM4B_Erastin) = 0.0106. **I** LAPTM4B stably expressing A549 cells and control cells were transfected with SLC7A11 siRNA. After 72 h, cells were harvested to measure lipid peroxidation. The left panel shows a representative experiment, while the right panel presents quantification data from n = 3 experiments, displayed as mean ± SEM. Statistical analysis revealed *p*(A549 Ctrl_NC siRNA, A549 Ctrl_SLC7A11 siRNA)=0.0097, *p*(A549 Ctrl_NC siRNA, A549 LAPTM4B_NC siRNA)=0.003, and *p*(A549 LAPTM4B_NC siRNA, A549 LAPTM4B_SLC7A11 siRNA) = 0.0186. **J** LAPTM4B stably expressing A549 cells and control cells were transfected with SLC7A11 siRNA and subsequently seeded into 96-well plates. After 72 h, the cells were stained with DAPI and EdU to visualize proliferative cells. Quantification data from *n* = 3 experiments are shown as mean ± SEM. Statistical analysis revealed *p*(A549 Ctrl_NC siRNA, A549 Ctrl_SLC7A11 siRNA)=0.0012, *p*(A549 Ctrl_NC siRNA, A549 LAPTM4B_NC siRNA)=0.0002, and *p*(A549 LAPTM4B_NC siRNA, A549 LAPTM4B_SLC7A11 siRNA) = 2.542E−06.

To gain further insight into LAPTM4B’s role in ferroptosis, we next investigated whether overexpression of LAPTM4B attenuates ferroptosis. Interestingly, SLC7A11 protein levels increased by about 2–3 fold in LAPTM4B-expressing A549 cells compared to control cells, and similar results were observed in H1299 cells (Fig. 5A). Subsequently, we investigated the protein stability and poly-ubiquitination status of SLC7A11 and found that the degradation of SLC7A11 protein was slower in LAPTM4B stably expressing cells during cycloheximide treatment (Fig. 5B, Supplementary Fig. S6A). As anticipated, reduced poly-ubiquitination of SLC7A11 was observed in LAPTM4B-expressing NSCLC cells (Fig. 5C). Furthermore, in LAPTM4B stable-expressing cells and control cells, siRNA targeting NEDD4L and ZRANB1 attenuated the differential levels of SLC7A11 (Fig. 5D, Supplementary Fig. S6B). Subsequent ubiquitination assays revealed that knockdown of NEDD4L/ZRANB1 nullified the diminished ubiquitination levels of SLC7A11 observed in LAPTM4B-overexpressing cells (Fig. 5E, Supplementary Fig. S6C).

Next, we examined whether overexpression of LAPTM4B could counteract ferroptosis. To this end, we measured key ferroptosis parameters, including lipid peroxidation and MDA. Overexpression of LAPTM4B resulted in decreased levels of lipid peroxidation (Fig. 5F, Supplementary Fig. S6D). Moreover, MDA levels were also reduced in LAPTM4B stably expressing cells compared to the control cells (Fig. 5G). Furthermore, we assessed the cellular effects of LAPTM4B in erastin-induced ferroptosis. The EdU staining reported that erastin treatment caused a moderate but significant decrease in the percentage of proliferative cells in the control group, which was rescued by LAPTM4B overexpression (Fig. 5H, Supplementary Fig. S6E, F). Additionally, colony formation experiments showed more colonies were observed in LAPTM4B stable expressing cells upon erastin treatment (Supplementary Fig. S7A, B).

To confirm that the protective role of LAPTM4B in erastin-induced ferroptosis is indeed mediated by SLC7A11, we downregulated SLC7A11 expression through siRNA transient transfection in both LAPTM4B stably expressing A549 cells and control cells (Supplementary Fig. S7C). Interestingly, silencing SLC7A11 in LAPTM4B stably expressing cells induced ferroptosis, as evidenced by increased levels of lipid peroxidation (Fig. 5I). The similar results were obtained in H1299 cells, suggesting it is not cell-specific effect (Supplementary Fig. S7D–F). Besides, silencing of SLC7A11 abolished the cellular protective role of LAPTM4B upon erastin treatment, as demonstrated by EdU staining (Fig. 5J, Supplementary Fig. S7G). These rescue experiments highlighted LAPTM4B’s regulatory role of ferroptosis is dependent on SLC7A11.

Collectively, our data indicate that LAPTM4B counteracts ferroptosis and protects cells from ferroptosis-related damage via promoting the stability of SLC7A11 in vitro.

### Loss of LAPTM4B accelerates ferroptosis-mediated suppression of tumor growth in vivo

**Fig. 6:**
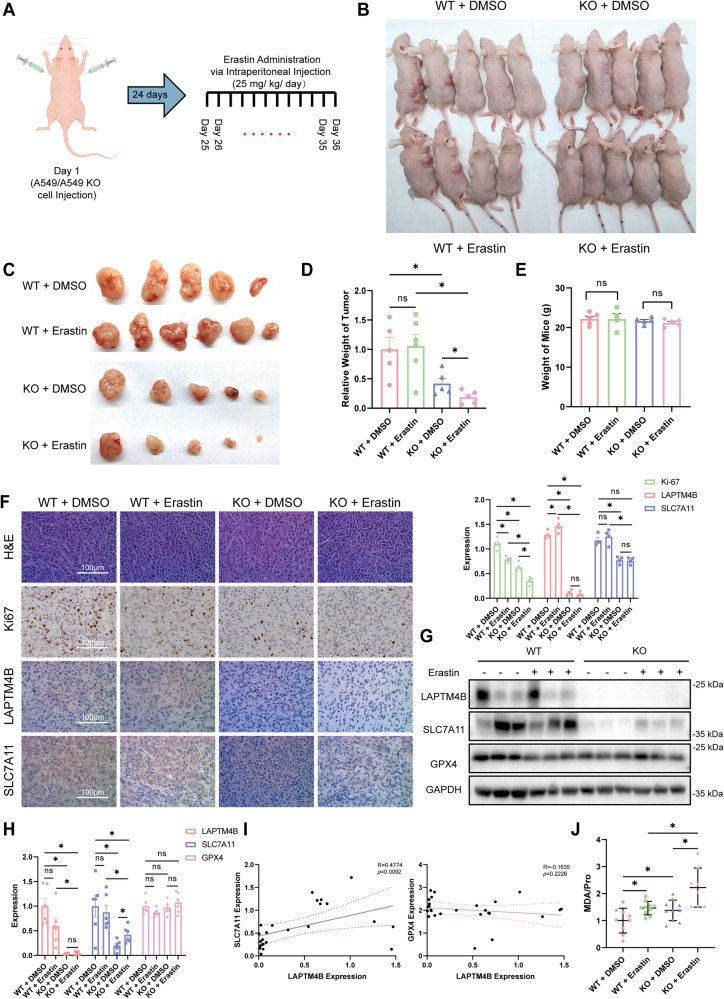
Loss of LAPTM4B accelerates ferroptosis-mediated suppression of tumor growth in vivo. **A** Flowchart depicting the experimental design of the nude mice xenograft study. Female nude mice were injected with 6 × 10^6^ WT or LAPTM4B KO A549 cells into the flanks. Once viable tumors had formed after 24 days, the mice were randomly divided into two subgroups, and administered 25 mg/kg/day of erastin or vesicle via intraperitoneal injections. The mice were sacrificed after 12 days of treatment. **B** Photographs of the mice at the time of sacrifice. **C** Photographs of the tumors used for further analysis. Note: Tumors exhibiting the highest and lowest weights within each experimental group were excluded from further analyses, due to the observed variability in tumor size. **D** Relative tumor weight in the mice with different treatments. Data presented as mean ± SEM, normalized to “WT_DMSO”. *p*(WT_DMSO, KO_DMSO) = 0.0215, *p*(KO_DMSO, KO_Erastin) = 0.027, *p*(WT_Erastin, KO_Erastin) = 0.0017. **E** Mouse weight (grams) at the time of sacrifice. **F** Immunohistochemistry (IHC) staining of Ki67, LAPTM4B, and SLC7A11 in mouse tumor tissue samples. Left panel: representative images from H&E staining and IHC staining. Scale bar: 100 µm. Right panel: quantification of IHC results. Data from 26 images from at least 4 mice per group. Data presented as mean ± SEM, normalized to “WT_DMSO”. Statistical analysis revealed the following p-values: For Ki67 staining, *p*(WT_DMSO, WT_Erastin)=0.0004, *p*(WT_DMSO, KO_DMSO) = 0.0005, *p*(WT_DMSO, KO_Erastin) = 4.35E−05, *p*(WT_Erastin, KO_Erastin) = 4.716E−05, *p*(KO_DMSO, KO_Erastin) = 0.0018. For LAPTM4B staining, *p*(WT_DMSO, WT_Erastin) = 0.0324, *p*(WT_DMSO, KO_DMSO) = 0.0001, *p*(WT_Erastin, KO_Erastin) = 4.924E−07, *p*(WT_DMSO, KO_Erastin) = 2.16E−07. For SLC7A11 staining, *p*(WT_DMSO, KO_DMSO) = 0.0009, *p*(WT_Erastin, KO_Erastin) = 0.0005. **G** Western blotting analysis of LAPTM4B, SLC7A11, and GPX4 protein levels in mouse tumor tissue samples. Representative experiments shown. **H** Quantification of Western blotting results from (**G**). Statistical analysis revealed the following *p* values: For LAPTM4B, *p*(WT_DMSO, KO_DMSO) = 0.0001, *p*(WT_DMSO, KO_Erastin) = 0.0001, *p*(WT_Erastin, KO_Erastin) = 0.0058. For SLC7A11, *p*(WT_DMSO, KO_DMSO) = 0.0052, *p*(WT_DMSO, KO_ Erastin) = 0.0309, *p*(KO_DMSO, KO_Erastin) = 0.0156, p(WT_Erastin, KO_Erastin) = 0.0156. **I** Correlation between expression levels of LAPTM4B and SLC7A11 (Left Panel) in mouse tumor samples (*R* = 0.4774, *p* = 0.0092), and correlation between expression levels of LAPTM4B and GPX4 (Right Panel, *R* = −0.1635, *p* = 0.2226). **J** Measurement of malondialdehyde (MDA) in tumor samples from mice with different treatments. Data from 15 tissues from at least 4 mice per group. Quantification of *n* = 3 experiments, presented as mean ± SEM, normalized to “WT_DMSO”. Statistical analysis revealed the following *p* values: *p*(WT_DMSO, WT_Erastin)=0.0066, *p*(WT_DMSO, KO_DMSO) = 0.033, *p*(WT_Erastin, KO_Erastin)=0.0034, *p*(KO_DMSO, KO_Erastin) = 0.0029.

Next, we aimed to assess the biological function of LAPTM4B in vivo following erastin treatment. To this end, we employed a nude mouse xenograft model by injecting 6 × 10^6^ WT or LAPTM4B KO A549 cells into the flanks of female nude mice. Once viable tumors had formed (24 days later), we randomly divided each group into two subgroups. We next administered 25 mg/kg/day of erastin or vesicle via intraperitoneal injections, following modified protocols from previous studies [43, 44]. We monitored the tumor volumes to evaluate their growth until the mice were sacrificed after 12 days (Fig. 6A). The mice showed distinguishable differences of tumor sizes between the mice injected with WT and KO cells (Fig. 6B, C). Interestingly, erastin treatment significantly reduced the tumor size in the mice injected with LAPTM4B KO cells, but not in those injected with WT A549 cells (Fig. 6C). This observation was further supported by measurements of tumor mass (Fig. 6D). Importantly, the body weights of the mice display no significant alteration, suggesting that the administered amount of erastin was well tolerated in vivo with negligible side effects (Fig. 6E). These findings indicate that the combined LAPTM4B depletion with erastin treatment exerted a significant suppression of tumor growth in vivo.

Further examination of the tumor samples by immunohistochemistry revealed increased SLC7A11 staining in LAPTM4B-positive tumor samples (Fig. 6F). This finding was supported by western blotting, showing an enhanced level of SLC7A11 in tumor samples formed by WT cells (Fig. 6G, H), consistent with the findings in cultured cells (Fig. 3A). Moreover, in these tumor samples from nude mice, the expression of LAPTM4B positively correlated with expression of SLC7A11, but not GPX4 (Fig. 6I). Additionally, to better evaluate the percentage of ferroptotic cells within the tissue, snap-frozen tumor samples from the nude mice were lysed and subjected to MDA measurement. Our data demonstrated higher levels of MDA in tumors formed by LAPTM4B KO cells compared to those from WT cells (Fig. 6J). Moreover, erastin treatment induced more pronounced increase of MDA levels in the mice injected with LAPTM4B KO cells, compared to WT cells (Fig. 6J). Collectively, these observations established that LAPTM4B depletion enhances the anti-tumor growth effect of erastin in vivo.

### LAPTM4B regulatory function on ferroptosis provides a potential therapeutic target in NSCLC

**Fig. 7:**
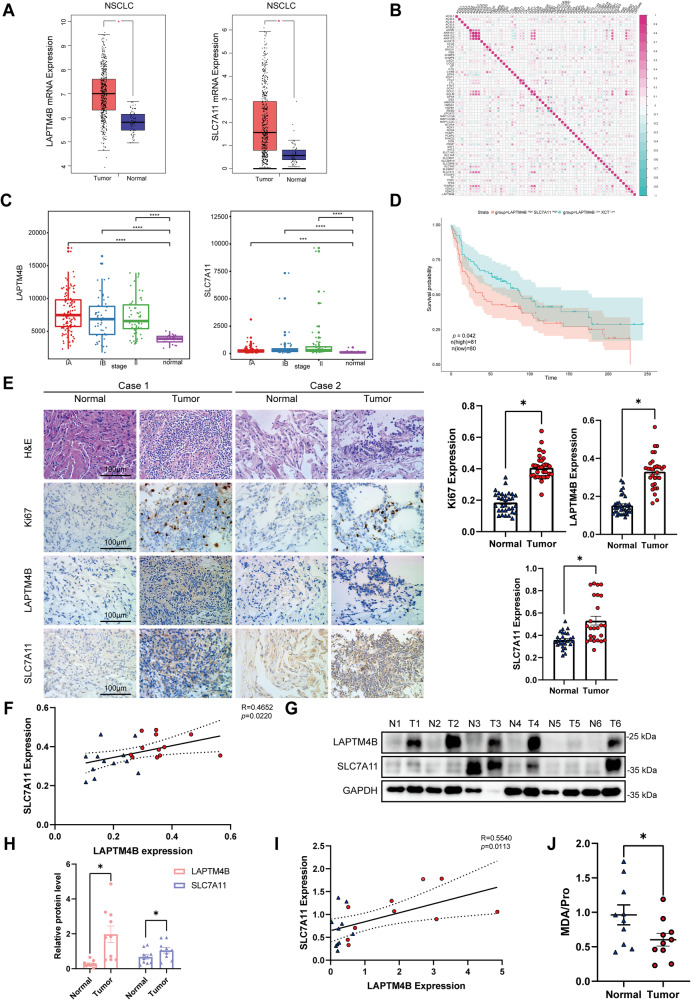
LAPTM4B as a potential therapeutic target in NSCLC through its regulatory function on ferroptosis. **A** Expression levels (log_2_^(TPM + 1)^) of LAPTM4B and SCL7A11 in NSCLC tumor tissues and adjacent normal tissues from the combined analysis of TCGA. Data presented as mean ± SEM, *p* < 0.05. “Normal tissues” represent “adjacent normal tissues”. **B** Mining and analysis of the association between LAPTM4B and central regulators of ferroptosis from the TCGA database. Correlation visualized by a heat map. “Red” indicates positive correlation, “Blue” indicates negative correlation. **C** Expression levels of LAPTM4B and SLC7A11 in different stages of NSCLC patients obtained from GEO (GSE:31210). **D** Overall survival probability of NSCLC patients based on LAPTM4B and SLC7A11 expression from the combined analysis of GEO (GSE:30219). **E** Immunohistochemistry (IHC) staining of LAPTM4B and SLC7A11 protein levels in tumor tissue and adjacent normal tissues from collected NSCLC surgery samples. Left panel: representative images from H&E staining and IHC staining. Right panel: quantification of IHC results. Data presented as mean ± SEM, normalized to “Normal”. For Ki67 expression, *p*(Normal, Tumor) = 1.13E−18. For LAPTM4B expression, *p*(Normal, Tumor) = 5.69E−13. For SLC7A11 expression, *p*(Normal, Tumor) = 0.0001. Scale bar: 100 µm. **F** Correlation between expression levels of LAPTM4B and SLC7A11 in patients’ tissue samples (*R* = 0.4652, *p* = 0.022), based on the results from IHC staining. The red color represents “NSCLC tumor tissue”, blue color represents “adjacent normal tissue”. (G). Western blotting analysis of LAPTM4B and SLC7A11 protein levels in tumor tissue and adjacent normal tissues from collected NSCLC surgery samples. **H** Quantification of results in (**G**). *N* = 3 technical repeats, mean ± SEM, normalized to “Normal”. For LAPTM4B expression, *p*(Normal, Tumor) = 0.001. For SLC7A11 expression, *p*(Normal, Tumor) = 0.0396. T: Tumor tissue; N: Adjacent normal tissue. **I** Correlation between expression levels of LAPTM4B and SLC7A11 in patients’ tissue samples (*R* = 0.554, *p* = 0.0113), based on the results from Western blotting. The red color represents “NSCLC tumor tissue”, blue color represents “adjacent normal tissue”. **J** Levels of malondialdehyde (MDA) in tumor tissue and adjacent normal tissues from collected NSCLC surgery samples. Quantification of *n* = 3 experiments, mean ± SEM, normalized to “Normal”. *p*(Normal, Tumor) = 0.0028.

Due to the function of LAPTM4B in regulating ferroptosis both in vitro and in vivo, we proceeded to investigate the clinical implications of our findings. Initially, we analyzed publicly available databases TCGA and GTEx to assess the relevance of LAPTM4B and SLC7A11 in tumor samples compared to adjacent normal tissue. Tumor samples show elevated transcript expression of both LAPTM4B and SLC7A11 (Fig. 7A). Interestingly, LAPTM4B expression correlated with several ferroptosis associated proteins, including SLC7A11, ACSL5, and ACSL6 (Fig. 7B). Further analysis from GEO (GSE#31210) showed that the expression of LAPTM4B and SLC7A11 was significantly increased in tumor samples compared to normal tissue (Fig. 7C). Furthermore, we utilized the Kaplan-Meier method to calculate overall survival of NSCLC patients. The results indicated that NSCLC patients with high expression of LAPTM4B and SLC7A11 (LAPTM4B^high^/SLC7A11^high^) has poor survival compared to the patients with low expression of LAPTM4B and SLC7A11 (LAPTM4B^low^/SLC7A11^low^) (Fig. 7D).

To further validate these findings, we performed immunohistochemistry (IHC) to evaluate LAPTM4B and SLC7A11 expression in 12 paired of NSCLC tissues and adjacent normal tissues. Representative images depicting different expression levels of LAPTM4B and SLC7A11 are presented (Fig. 7E, Supplementary Fig. S8A). Notably, high LAPTM4B protein expression was observed in 83.3% (10/12) of NSCLC tissues, whereas 75% (9/12) of adjacent normal tissues displayed low LAPTM4B expression (Fig. 7E). Similarly, high SLC7A11 protein expression was observed in 66.7% (8/12) of NSCLC tissues, while 75% (9/12) of adjacent normal tissues exhibited low SLC7A11 expression (Fig. 7E). Interestingly, the levels of LAPTM4B and SLC7A11 are significantly correlated (Fig. 7F). These expression patterns and correlations were further confirmed through western blotting using snap-frozen patient samples (Fig. 7G–I, Supplementary Fig. S8B). To evaluate the level of ferroptosis, we measured the amounts of MDA, our data indicated that patients’ tumor samples with low expression of either LAPTM4B or SLC7A11 had higher MDA levels (Fig. 7J). These findings suggest that LAPTM4B and SLC7A11 are coordinately involved in regulating ferroptosis in NSCLC patients, which further influences the progression of NSCLC and the survival probability of patients.

### The anti-ferroptosis effect of LAPTM4B could be universal in other types of cancer

LAPTM4B has been identified as an oncogenic protein that promotes malignance in various cancers [45]. However, it remains unclear whether LAPTM4B’s regulatory role on ferroptosis extends to other types of cancer. To this end, we selected Hela, A431, and PC3 as representative cells to investigate the potential involvement of LAPTM4B in cervical cancer, epidermoid cancer, and prostate cancer. For each cancer cell line, we knocked out LAPTM4B via utilizing CRISPR-Cas9 (Supplementary Fig. S9A–C). To uncover common metabolic pathways regulated by LAPTM4B, we performed metabolomic screening of soluble intracellular metabolites using Swedish Metabolomics Centre in-house spectral library, as described previously [46].

**Fig. 8:**
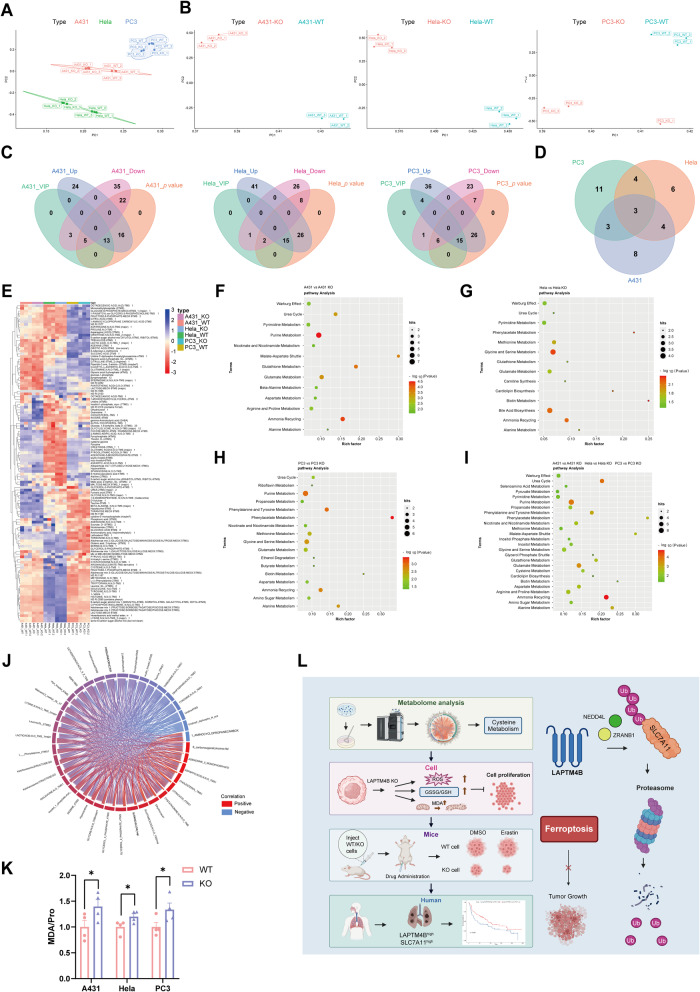
The anti-ferroptosis effect of LAPTM4B could be universal in other types of cancer. **A** Principal component analysis (PCA) of metabolomics profiling results in WT and KO A431, WT and KO Hela, and WT and KO PC3 cells. Three independent replicates are shown. **B** PCA plot of metabolomics profiling induced by LAPTM4B depletion within each individual cell line. **C** Selection of significantly perturbed metabolites by LAPTM4B depletion: 18 metabolites in A431 cells, 17 metabolites in Hela cells, and 21 metabolites in PC3 cells, after threshold (VIP > 1, *p* < 0.05) selection. **D** Union set of altered metabolites regulated by LAPTM4B among the three cell lines. **E** Heatmap displaying altered metabolites in LAPTM4B-depleted cell lines. **F** KEGG analysis of significantly altered pathways regulated by LAPTM4B in A431 cells (*p* < 0.05). **G** KEGG analysis of significantly altered pathways regulated by LAPTM4B in Hela cells (*p* < 0.05). **H** KEGG analysis of significantly altered pathways regulated by LAPTM4B in PC3 cells (*p* < 0.05). **I** KEGG analysis of significantly altered pathways regulated by LAPTM4B, based on the data intersection from A431, Hela, and PC3 cells (*p* < 0.05). **J** Circos plot was employed to visualize the metabolites and metabiotic progress regulated by LAPTM4B. **K** Measurement of malondialdehyde (MDA) in WT and LAPTM4B KO cells. Quantification of *n* = 4 experiments, mean ± SEM. Data normalized to “WT”. For A431 cells, *p*(WT, KO) = 0.003. For Hela cells, *p*(WT, KO) = 0.0045. For PC3 cells, *p*(WT, KO) = 0.0111. **L** Schematic diagram depicting the working model and the main findings in the current study, including the metabolic landscape regulated by LAPTM4B, the suppressive effect of LAPTM4B on cellular ferroptosis, and the protection of tumor cell growth from erastin-induced ferroptosis. These findings are supported by clinical patient samples and data mining of publicly available databases. The working model illustrates the detailed molecular mechanism, with LAPTM4B promoting SLC7A11 stability and protecting cells from ferroptosis through suppression of NEDD4L/ZRANB1-mediated proteasomal degradation. This mechanism stimulates NSCLC growth and progression.

The metabolomics screening successfully detected at least 119 metabolites in every individual cell line (Supplementary Table S3). Principal component analysis (PCA) demonstrated the clear separation between different cell lines (Fig. 8A), further analysis showed the shifted distribution of principle component induced by LAPTM4B depletion within each individual cell line (Fig. 8B). After applying the threshold (VIP ≥ 1, *p* < 0.05), we found that 18 metabolites were significantly perturbed by LAPTM4B depletion in A431 cells, 17 metabolites in Hela cells, and 21 metabolites in PC3 cells (Fig. 8C). The union set of altered metabolites among three cell lines suggests the vital elements potentially regulated by LAPTM4B (Fig. 8D, Supplementary Table S4), and the altered metabolites were displayed as heatmap (Fig. 8E). Notably, several common metabolites (e.g. Glycine, Glutamine, Citric acid) were consistently enriched in LAPTM4B KO samples from all three cell lines (Fig. 8E). Subsequent pathway enrichment analysis of the top 20 metabolites in each individual cell line (Fig. 8F–H), together with intersection analysis of the data (Fig. 8I), demonstrated that LAPTM4B regulate Glutathione Metabolism, Glutamate Metabolism, and Cysteine metabolism, which are central in ferroptosis. We next performed the Circos plot, our results highlighted the metabolites and metabiotic progress specifically regulated by LAPTM4B, such as glutamic acid, leucine, and glycine (Fig. 8J). We further performed malondialdehyde (MDA) measurements to assess ferroptosis in A431, PC3 and Hela WT and LAPTM4B KO cells. LAPTM4B depletion leads to elevated MDA levels in all cell types, indicating that LAPTM4B suppresses ferroptosis in several different types of cancer cells (Fig. 8K).

In summary, this study uncovered the metabolic landscape controlled by LAPTM4B in NSCLC. We demonstrate, for the first time, that LAPTM4B counteracts cellular ferroptosis. Various cellular assays and animal experiments strengthen the notion that LAPTM4B protects tumor cell growth from erastin-induced ferroptosis both in vitro and in vivo. Mechanistically, we established that LAPTM4B stabilizes SLC7A11 by suppressing the NEDD4L/ZRANB1 mediated ubiquitination and proteasomal degradation. Additionally, the findings from patient samples and data mining of publicly available databases underscore the clinical relevance of the mechanism, highlighting the LAPTM4B-SLC7A11-ferroptosis signaling axis as a potential therapeutic target (Fig. 8L).

## Discussion

Numerous studies have demonstrated an oncogenic role of LAPTM4B in various cancers [45]. LAPTM4B has been reported to regulate lysosomal leucine uptake [30] and cellular lipid signatures [32], but the comprehensive metabolic landscape controlled by LAPTM4B has not been previously studied. To the best of our knowledge, this study represents the first systematic profiling of LAPTM4B’s role in cellular metabolism. Using CRISPR-Cas9 and unbiased metabolomic approaches, we profiled a wide range of metabolites, allowing us to dissect metabolic pathways and related biological processes regulated by LAPTM4B. Our study established the inhibitory role of LAPTM4B in ferroptosis. These findings were further validated in resected samples from NSCLC patients and cancer databases.

Given that ferroptosis-related therapies have emerged as promising anti-cancer treatment strategies, with several FDA-approved drugs already available, such as sorafenib, sulfasalazine, statins, and artemisinin [6, 47, 48], we believe that developing small-molecule drugs targeting the LAPTM4B-SLC7A11 axis holds significant clinical potential. The animal experiments further support this notion, as combined treatment of ferroptosis inducers with LAPTM4B depletion resulted in a significant reduction of tumor growth with negligible side effects.

System Xc^−^ (consisting of regulatory subunit SLC7A11 and component subunit SLC3A2) acts a central role in ferroptosis regulation, with most studies focusing on transcriptional regulation [49]. Several transcription factors, such as p53, ATF, and NRF2 (nuclear factor erythroid 2-like 2), have been identified in regulating the expression of SLC7A11 [49, 50]. However, the protein stability and degradation of System Xc^−^, particularly through lysosome-derived signaling, remain poorly understood. In the current study, we observed that LAPTM4B significantly enhances SLC7A11 levels by inhibiting ubiquitin-proteasome degradation. The potential protein interaction between LAPTM4B and SLC7A11 was confirmed through immunoprecipitation and immunofluorescence experiments. We hypothesize that this interaction may underlie the regulatory function of LAPTM4B in stabilizing SLC7A11. However, the specific interacting motifs and detailed molecular mechanisms still require further investigation. Our study highlights a scenario in which a lysosomal protein participates in ferroptosis by controlling the protein stability of the central regulator, ultimately impacting tumor growth in vitro and in vivo.

Ferroptosis is triggered by excessive iron-dependent peroxidation of PUFA-containing phospholipids (PUFA-PLs) in cellular membranes [8]. In a previous lipidomic study, we found that LAPTM4B depletion led to increased levels of PUFA-containing ether lipids [32], which is in line with our current findings demonstrating LAPTM4B’s role in counteracting ferroptosis. NRF2 inhibits ferroptosis by promoting GSH synthesis [51]. Interestingly, a study by Maki *et al* demonstrated that LAPTM4B enhances the expression and nuclear translocation of NRF2 under nutrient stress, thereby increasing the transcription of NRF2 targeted genes, including NQO1 and ME1, which regulate NADPH homeostasis and inhibit ROS [18]. The data from Maki et al. not only support our current findings but also provide another potential molecular mechanism underlying LAPTM4B’s regulatory role in ferroptosis [18].

In conclusion, we have established that LAPTM4B suppresses ferroptosis and protects cells from erastin-induced ferroptosis, shedding light on LAPTM4B’s oncogenic role. Mechanistically, we have uncovered that LAPTM4B increased the SLC7A11 protein level via suppressing the NEDD4L/ZRANB1 mediated ubiquitination and proteasome degradation. Moreover, the observed anti-ferroptosis function of LAPTM4B and the potential clinical significance were further supported by the data in animal experiments, in NSCLC patients’ samples, as well as in the cancer databases, underscoring that the anti-ferroptosis role of LAPTM4B might promote NSCLC progression and diminish patients’ survival probability. Additionally, we extended the current discovery to other cancers, these findings can serve as one rationale for future targeted therapeutics development of cancers. Even though our data from clinical NSCLC patients’ samples and cancer databases encouragingly support the findings from cells and the xenograft mice model, other methodologies especially transgenic mice (e.g. LAPTM4B KO mice) are needed for future studies of LAPTM4B function in regulating cell metabolism, as well as during cancer development.

## Material and methods

### Human cell lines and tissue specimens

The non-small cell lung cancer (NSCLC) cell lines A549 and H1299, the epidermoid cancer cell line A431, the cervical cancer cell line Hela, and the prostate cancer cell line PC-3 were obtained from the American Type Culture Collection (ATCC). All cells were subjected to authentication through STR profiling and regular testing for mycoplasma contamination.

To generate LAPTM4B knockout (KO) cells, the CRISPR/Cas9n system was employed, following a previously established methodology [23, 30]. In brief, the coding sequences in exon 3 of LAPTM4B (Gene ID: 55353) were analyzed, and Cas9 nickase targets were designed (http://crispr.mit.edu). Guide RNAs (sgRNAs) were subcloned using BbsI sites. The cells were transfected with plasmids expressing Cas9 nickase and sgRNA, and then subjected to puromycin selection (2 µg/ml) for 48 h. Subsequently, single clones were isolated and confirmed via Sanger sequencing and western blotting.

For the generation of LAPTM4B stable overexpressing cell lines, the KO cells were transfected with pHAGE-P containing LAPTM4B-24-3xFlag using a Liposomal Transfection Reagent. These cells were cultured in a medium supplemented with puromycin (2 µg/ml) until a pool of resistant cells was obtained. All cells, including the KO and stable overexpressing cell lines, were cultured in DMEM supplemented with 10% FBS, l-glutamine, and penicillin/streptomycin at 37 °C with 5% CO_2_.

NSCLC cancer tissues were collected from 12 patients who underwent resection surgery. None of the patients had undergone treatment prior to surgery. The tumor samples were derived from primary tumors and classified according to the World Health Organization’s criteria. Hematoxylin and eosin (H&E) staining and immunohistochemistry (IHC) staining were performed using partial paraffin-embedded samples from 12 patients. Due to constraints in sample volume, two tumor samples were used up during the immunohistochemical procedure. Consequently, the remaining 10 paired tissues were utilized for western blotting and malondialdehyde measurement. This study was approved by the local research ethics committee, and written informed consent was obtained from all patients following the instructions from the Declaration of Helsinki.

### Q-PCR

For mRNA expression assay, total RNA was isolated from cells or tissues using RNA isolater Total RNA Extraction Reagent (Vazyme, Cat#R401-01). The cDNA was synthesized by Hifair®V one-step RT-gDNA digestion SuperMix for qPCR (Yeasen, Cat#11141ES60) using the StepOne Real-Time PCR System (Applied Biosystems, Foster City, CA, USA). All primers in our research were listed in Supplementary Table S5.

### Metabolome profiling of NSCLC cells

Metabolome profiling was conducted using a state-of-the-art UPLC-ESI-Q-Orbitrap-MS system (UHPLC, Shimadzu Nexera X2 LC-30AD, Shimadzu, Japan) coupled with Q-Exactive Plus (Thermo Scientific, San Jose, USA) to ensure high-quality data acquisition.

To maintain data consistency and reliability, quality control (QC) samples were prepared by pooling aliquots of all samples, representing the entirety of the samples under analysis, and were used for data normalization. The QC samples underwent the same sample preparation and analysis procedure as the experimental samples in each batch. Dried extracts were reconstituted in 50% acetonitrile, filtered using a disposable 0.22 µm cellulose acetate filter, transferred into 2 mL HPLC vials, and stored at −80 °C until analysis.

For liquid chromatography (LC) separation, samples were analyzed using an ACQUITY UPLC® HSS T3 column (2.1 × 100 mm, 1.8 μm) (Waters, Milford, MA, USA) with a flow rate of 0.3 mL/min. The mobile phase consisted of solvent A (0.1% formic acid in water) and solvent B (100% acetonitrile). The gradient was initiated at 0% buffer B for 2 min, followed by a linear increase to 48% over 4 min, then ramped up to 100% over 4 min and maintained for 2 min. Finally, a swift transition to 0% buffer B took place over 0.1 min, with a 3-min re-equilibration period.

Electrospray ionization (ESI) was employed in positive and negative modes separately for MS data acquisition. The HESI source conditions were set as follows: spray voltage of 3.8 kV (positive) and 3.2 kV (negative), capillary temperature at 320 °C, sheath gas (nitrogen) flow rate at 30 arbitrary units, auxiliary gas flow rate at 5 arbitrary units, probe heater temperature at 350 °C, and S-Lens RF Level at 50. The instrument was programmed to scan the m/z range of 70–1050 Da for full MS analysis. Full MS scans were acquired at a resolution of 70,000 at m/z 200, and MS/MS scans were acquired at a resolution of 17,500 at m/z 200. The maximum injection time was set to 100 ms for MS scans and 50 ms for MS/MS scans.

### Analysis of soluble intracellular metabolites

Soluble intracellular metabolites were extracted from snap frozen cell pellets. Metabolites were extracted on ice using 4 °C cold, 18 mg/ml methanol:water extraction mix (90:10 v/v) including labeled internal standards (2.5 ng/μl, myristic acid-13C3, proline-13C5 (Cil, Andover, MA, USA) and d-sucrose-13C12 (Sigma-Aldrich, St. Louis, MO, USA). Samples were homogenized using rigorous agitation at 30 Hz for 2 min in a bead mill (Retsch, MM 400) followed by proteins precipitation at −20 °C for 2 h, and centrifugation at 18,600 × *g* for 10 min at 4 °C. 200 μl extract were transferred to glass vials and evaporated until dry in a speedvac. Pooled quality control (QC) reference samples were included at the beginning and end of each analytical batch and as every tenth sample, for monitoring of platform performance. Dried samples were dissolved in pyridinic methoxyamine, derivatised and analysed by GC-EI-MS as previously described by us [46]. The identities of the resolved peaks were determined by comparing mass spectra and retention indices with data in the Swedish Metabolomics Centre in-house spectral library. NIST MS search software was used for manual verification of spectral identifications.

### Measurement of the lipid peroxidation level

The current study employed a fluorescence-based reporter BODIPY® 581/591 C11 (Thermo Fisher, Cat#C10445) to quantify the lipid peroxidation levels. The fluorescent group of the reagent undergoes a change from red to green upon oxidation in living cells. The absorption peaks at 510 nm (green) and 590 nm (red) are detected by flow cytometry, and the ratio of signals in the 590 channel and 510 channel is used to quantify the level of lipid peroxidation in cells.

### Measurement of malondialdehyde (MDA)

The current study employed the Lipid Oxidation (MDA) Assay Kit (Beyotime, Cat#S0131S) for the quantitative detection of malondialdehyde (MDA). This method relies on the chemical reaction between MDA and thiobarbituric acid (TBA), which leads to the formation of a red product that can be measured using spectrophotometry at 532 nm. This particular wavelength corresponds to the maximum absorption of the MDA-TBA adduct. By utilizing enzyme-linked immunosorbent assay (ELISA), the absorbance peak at 532 nm can be accurately measured, thereby facilitating the determination of MDA concentration.

### Measurement of GSH and GSSG

The current study employed GSH and GSSG Assay Kit (Beyotime, Cat#S0053) for the quantitative assessment of glutathione (GSSG) and glutathione (GSH). Briefly, we employed glutathione reductase to enzymatically convert oxidized glutathione (GSSG) into its reduced form glutathione (GSH). The resulting GSH subsequently reacted with the chromogenic substrate DTNB, leading to the formation of a yellow-colored compound known as TNB, as well as the regeneration of GSSG.

By appropriately configuring the reaction system and combining the two reactions, the total glutathione (2*GSSG + GSH) became the limiting factor for the production of color, with the amount of TNB formed directly correlated to the quantity of total glutathione present. Consequently, the total glutathione content could be determined by measuring the absorbance at 412 nm (A412). Furthermore, by selectively removing GSH from the sample employing suitable reagents, the aforementioned reaction principle could be effectively utilized to quantify the GSSG content. The GSH content, in turn, could be calculated by subtracting the GSSG content from the total glutathione (2*GSSG + GSH) content.

### In vivo tumor growth assay

All xenograft tumor growth experiments were conducted in compliance with animal experimentation regulations and approved by the ethics committee of Anhui Medical University (Approval number: 20200378).

Twenty female BALB/c nude mice (5 weeks old) were obtained from GemPharmatech Company (Nanjing, China) and housed under standard conditions. The mice were randomly assigned to different groups and injected with 6×10^6^ wild-type (WT) or LAPTM4B knockout (KO) A549 cells into their flanks. After 24 days, viable tumors had formed, and the mice in each group were further divided into two subgroups. One subgroup received intraperitoneal injections of 25 mg/kg/day erastin, while the other subgroup received control dimethyl sulfoxide (DMSO). The injections were administered once daily for 12 consecutive days. The mice were sacrificed afterwards, and the tumors in each group were histologically examined, counted, and subjected to statistical analysis. Variations in the tumors formed in mice across different groups were noted during the study. Additionally, the final number of mice in each group was not uniform due to an inadvertent sacrifice of one mouse in the “WT+Erastin” group during treatment. To address these inconsistencies, the maximum and minimum tumor weights from each group were excluded from subsequent analyses. In the animal experiment, the investigators were not blinded to the group allocation during the experiment.

### Data mining

TCGA and GTEx database, together with Gene Expression Omnibus (https://www.ncbi.nlm.nih.gov/geo/) were employed to investigate the expression of LAPTM4B or SLC7A11, as well as analyze the relationship between gene expression and cancer patients’ survival probability.

### Statistical analysis

All the data are presented as the mean ± standard error of the mean (SEM) from at least three independent experiments. Statistical significance was calculated using the Student’s t-test for pairwise comparisons. * indicates *p* < 0.05.

Additional detailed descriptions of methods are in Supplementary Materials and methods.

### Supplementary information

Supplementary information

Supplementary Table S1

Supplementary Table S2

Supplementary Table S3

Supplementary Table S4

Supplementary Table S5

Original Western Blot

## Data Availability

All the data generated during the current study are available from the corresponding author on reasonable request.
