# Supplementary material for: LAPTM4B counteracts ferroptosis via suppressing the ubiquitin-proteasome degradation of SLC7A11 in non-small cell lung cancer
Source: Cell Death Dis. 2024 Jun 20;15(6):436. doi: 10.1038/s41419-024-06836-x (PMC11190201; doi:10.1038/s41419-024-06836-x)
Supplement: Supplementary file 1 — Supplementary information [file 41419_2024_6836_MOESM1_ESM.pdf]

LAPTM4B counteracts ferroptosis via suppressing the ubiquitin-proteasome degradation of SLC7A11 in non-small cell lung cancer

Ruyu Yan[1,#], Dan Liu[1,#], Hongjuan Guo[1], Minxia Liu[1,2], Dongjin Lv[3], Benny Björkblom[4], Mingsong Wu[5], Hongtao Yu[1], Hao Leng[1], Bingxiao Lu[6], Yuxiang Li[1], Miaomiao Gao[1], Tomas Blom[2,7*], Kecheng Zhou[1,2,7*]

[1] School of Life Sciences, Anhui Medical University, Hefei, 230032, China

[2] Faculty of Medicine, University of Helsinki, Helsinki, 00014, Finland

[3] Department of Clinical Research, The Third Affiliated Hospital of Kunming Medical University (Tumor Hospital of Yunnan Province), Kunming, China

[4] Department of Chemistry, Umeå University, Umeå, 90187, Sweden

[5] School of Stomatology, Zunyi Medical University, Zunyi, Guizhou, 563000, China

[6] Department of Medical Oncology, The Third Affiliated Hospital of Kunming Medical University (Tumor Hospital of Yunnan Province), Kunming, China

[7] Minerva Foundation Institute for Medical Research, Helsinki, 00014, Finland

[#] Ruyu Yan and Dan Liu contribute equally to the current study

Supplementary Information

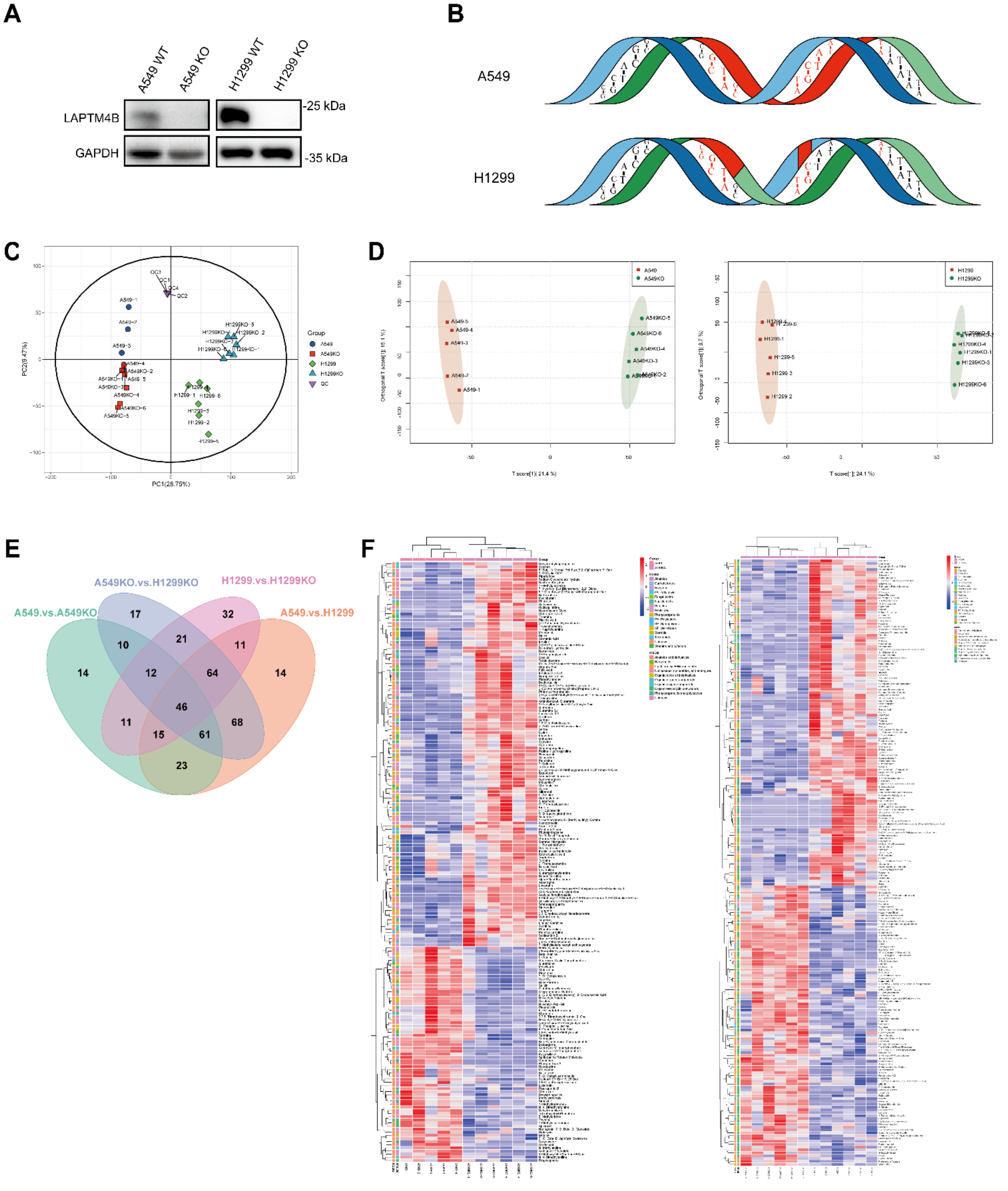

Supplementary Figure S1. Metabolic alterations induced by LAPTM4B depletion

(A) Western blot analysis of LAPTM4B expression levels in wild-type (WT) and LAPTM4B knockout (KO) A549 cells and WT and LAPTM4B KO H1299 cells.

(B) DNA sequence analysis showing DNA deletion and mutation in LAPTM4B KO A549 or H1299 cells, resulting in "out-of-frame" translation or early stop codon.

(C) Principal component analysis (PCA) of metabolomics profiling results in WT and KO A549 cells and WT and KO H1299 cells, based on positive mode (POS) data. Shown are replicates of 5-6 independent experiments.

(D) Orthogonal partial least squares-discriminant analysis (OPLS-DA) plot of metabolites perturbed by LAPTM4B depletion in A549 (left panel) and H1299 cells (right panel), based on POS data.

(E) Intersection of altered metabolites in WT and LAPTM4B KO cells, with 84 metabolites commonly enriched in LAPTM4B KO samples from both cell lines, based on POS data.

(F) Heatmap showing altered metabolites in WT and KO A549 cells (left panel) and WT and KO H1299 cells (right panel), based on POS data. Upregulation is indicated by red, while downregulation is indicated by blue.

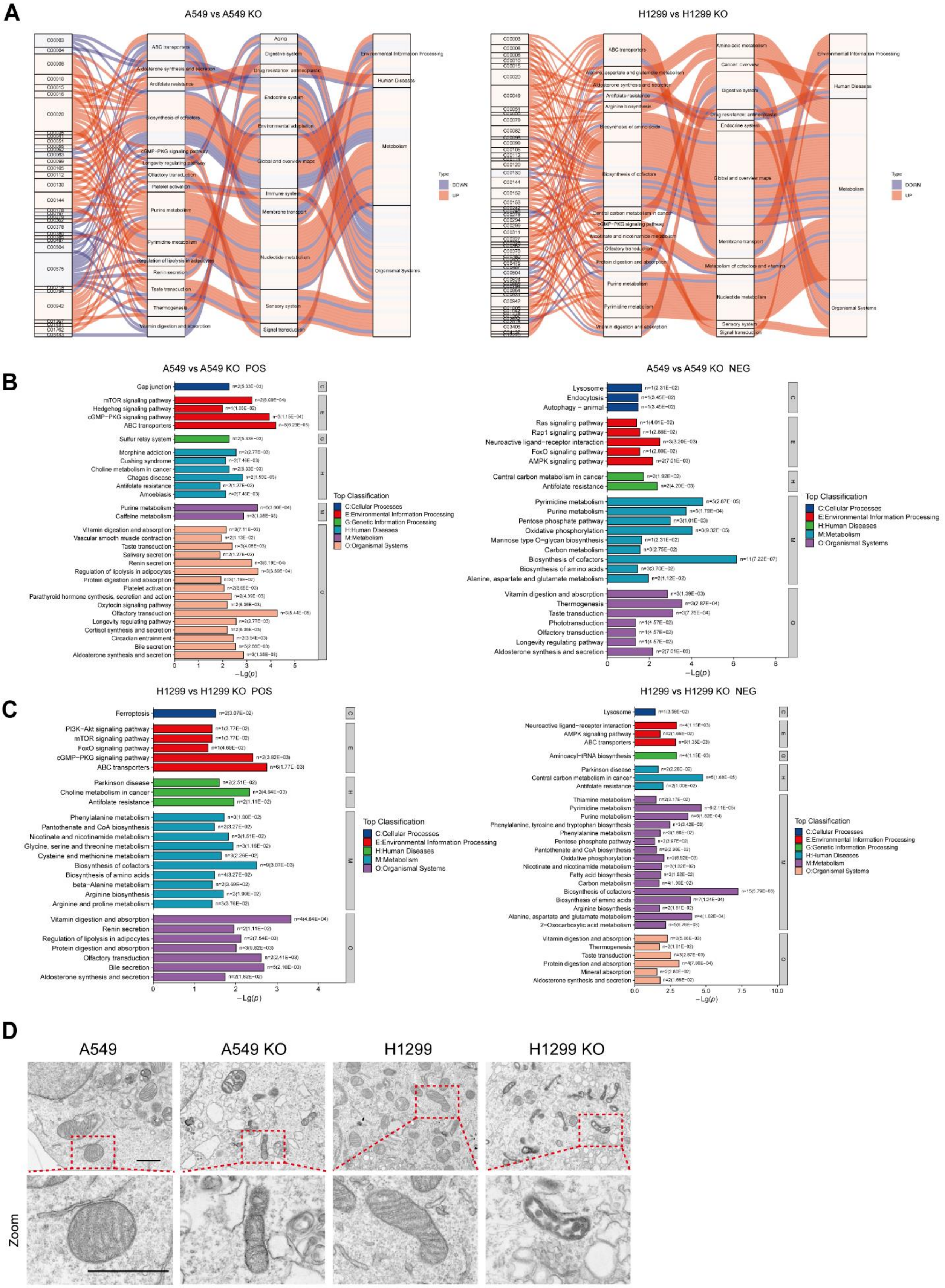
A
A549 vs A549 KO
H1299 vs H1299 KO
Type
DOWN
UP
B
A549 vs A549 KO POS
Gap junction
mTOR signaling pathway
Hedgehog signaling pathway
cGMP−PKG signaling pathway
ABC transporters
Sulfur relay system
Morphine addiction
Cushing syndrome
Choline metabolism in cancer
Chagas disease
Antifolate resistance
Amoebiasis
Purine metabolism
Caffeine metabolism
Vitamin digestion and absorption
Vascular smooth muscle contraction
Taste transduction
Salivary secretion
Renin secretion
Regulation of lipolysis in adipocytes
Protein digestion and absorption
Platelet activation
Parathyroid hormone synthesis, secretion and action
Oxytocin signaling pathway
Olfactory transduction
Longevity regulating pathway
Cortisol synthesis and secretion
Circadian entrainment
Bile secretion
Aldosterone synthesis and secretion
−Lg(p)
Top Classification
C:Cellular Processes
E:Environmental Information Processing
G:Genetic Information Processing
H:Human Diseases
M:Metabolism
O:Organismal Systems
A549 vs A549 KO NEG
Lysosome
Endocytosis
Autophagy − animal
Ras signaling pathway
Rap1 signaling pathway
Neuroactive ligand−receptor interaction
FoxO signaling pathway
AMPK signaling pathway
Central carbon metabolism in cancer
Antifolate resistance
Pyrimidine metabolism
Purine metabolism
Pentose phosphate pathway
Oxidative phosphorylation
Mannose type O−glycan biosynthesis
Carbon metabolism
Biosynthesis of cofactors
Biosynthesis of amino acids
Alanine, aspartate and glutamate metabolism
Vitamin digestion and absorption
Thermogenesis
Taste transduction
Phototransduction
Olfactory transduction
Longevity regulating pathway
Aldosterone synthesis and secretion
−Lg(p)
Top Classification
C:Cellular Processes
E:Environmental Information Processing
H:Human Diseases
M:Metabolism
O:Organismal Systems
C
H1299 vs H1299 KO POS
Ferroptosis
PI3K−Akt signaling pathway
mTOR signaling pathway
FoxO signaling pathway
cGMP−PKG signaling pathway
ABC transporters
Parkinson disease
Choline metabolism in cancer
Antifolate resistance
Phenylalanine metabolism
Pantothenate and CoA biosynthesis
Nicotinate and nicotinamide metabolism
Glycine, serine and threonine metabolism
Cysteine and methionine metabolism
Biosynthesis of cofactors
Biosynthesis of amino acids
beta−Alanine metabolism
Arginine biosynthesis
Arginine and proline metabolism
Vitamin digestion and absorption
Renin secretion
Regulation of lipolysis in adipocytes
Protein digestion and absorption
Olfactory transduction
Bile secretion
Aldosterone synthesis and secretion
−Lg(p)
Top Classification
C:Cellular Processes
E:Environmental Information Processing
H:Human Diseases
M:Metabolism
O:Organismal Systems
H1299 vs H1299 KO NEG
Lysosome
Neuroactive ligand−receptor interaction
AMPK signaling pathway
ABC transporters
Aminoacyl−tRNA biosynthesis
Parkinson disease
Central carbon metabolism in cancer
Antifolate resistance
Thiamine metabolism
Pyrimidine metabolism
Purine metabolism
Phenylalanine, tyrosine and tryptophan biosynthesis
Phenylalanine metabolism
Pentose phosphate pathway
Pantothenate and CoA biosynthesis
Oxidative phosphorylation
Nicotinate and nicotinamide metabolism
Fatty acid biosynthesis
Carbon metabolism
Biosynthesis of cofactors
Biosynthesis of amino acids
Arginine biosynthesis
Alanine, aspartate and glutamate metabolism
2−Oxocarboxylic acid metabolism
Vitamin digestion and absorption
Thermogenesis
Taste transduction
Protein digestion and absorption
Mineral absorption
Aldosterone synthesis and secretion
−Lg(p)
Top Classification
C:Cellular Processes
E:Environmental Information Processing
G:Genetic Information Processing
H:Human Diseases
M:Metabolism
O:Organismal Systems
D
A549
A549 KO
H1299
H1299 KO
Zoom

Supplementary Figure S2. LAPTM4B depletion induces ferroptosis-related metabolites and metabolic processes

(A) Sankey diagrams illustrating the top 15 enriched pathways based on LAPTM4B perturbed metabolites in A549 cells (left panel) and H1299 cells (right panel). The diagrams depict the associations and flow of metabolites, metabolic processes, and potential diseases.

(B) KEGG analysis of the top 30 pathways regulated by LAPTM4B in A549 cells. Left panel: Analysis based on positive mode (POS) data. Right panel: Analysis based on negative mode (NEG) data.

(C) KEGG analysis of the top 30 pathways regulated by LAPTM4B in H1299 cells. Left panel: Analysis based on POS data. Right panel: Analysis based on NEG data.

(D) Transmission electron microscopy analysis of mitochondrial ultrastructure in wild-type (WT) and LAPTM4B knockout (KO) A549 cells, as well as in WT and KO H1299 cells. The plot displays representative images of swollen and degenerated mitochondria. Scale bar: 1 µm. The region within the dashed red box is amplified in the lower panel.

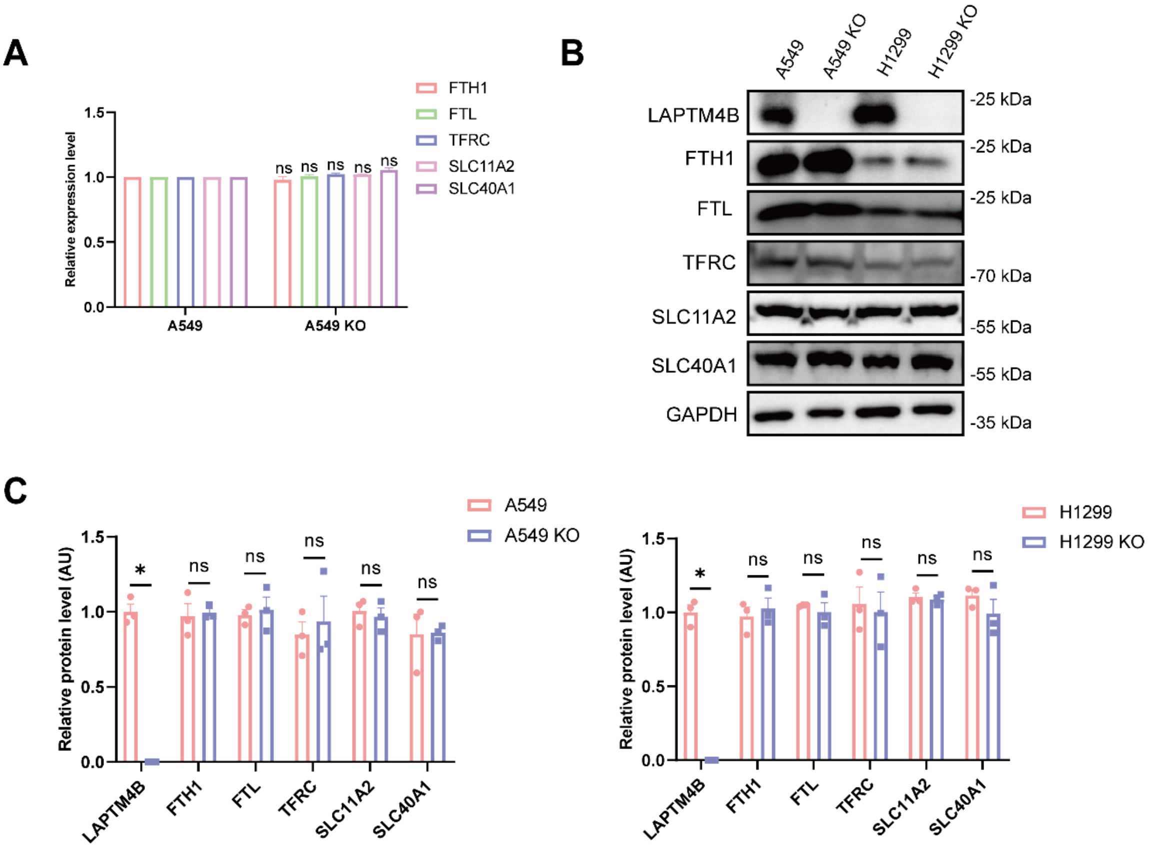

Supplementary Figure S3. LAPTM4B does not regulate the expression of crucial iron transferrin proteins in NSCLC cells

(A) Expression levels of crucial iron transferrin proteins measured by quantitative polymerase chain reaction (Q-PCR).

(B) Western blot analysis of crucial iron transferrin proteins in LAPTM4B-depleted A549 and H1299 cells.

(C) Quantification of Western blotting results from (B). Quantification of at least three experiments, presented as mean ± SEM. * $p<0.05$.

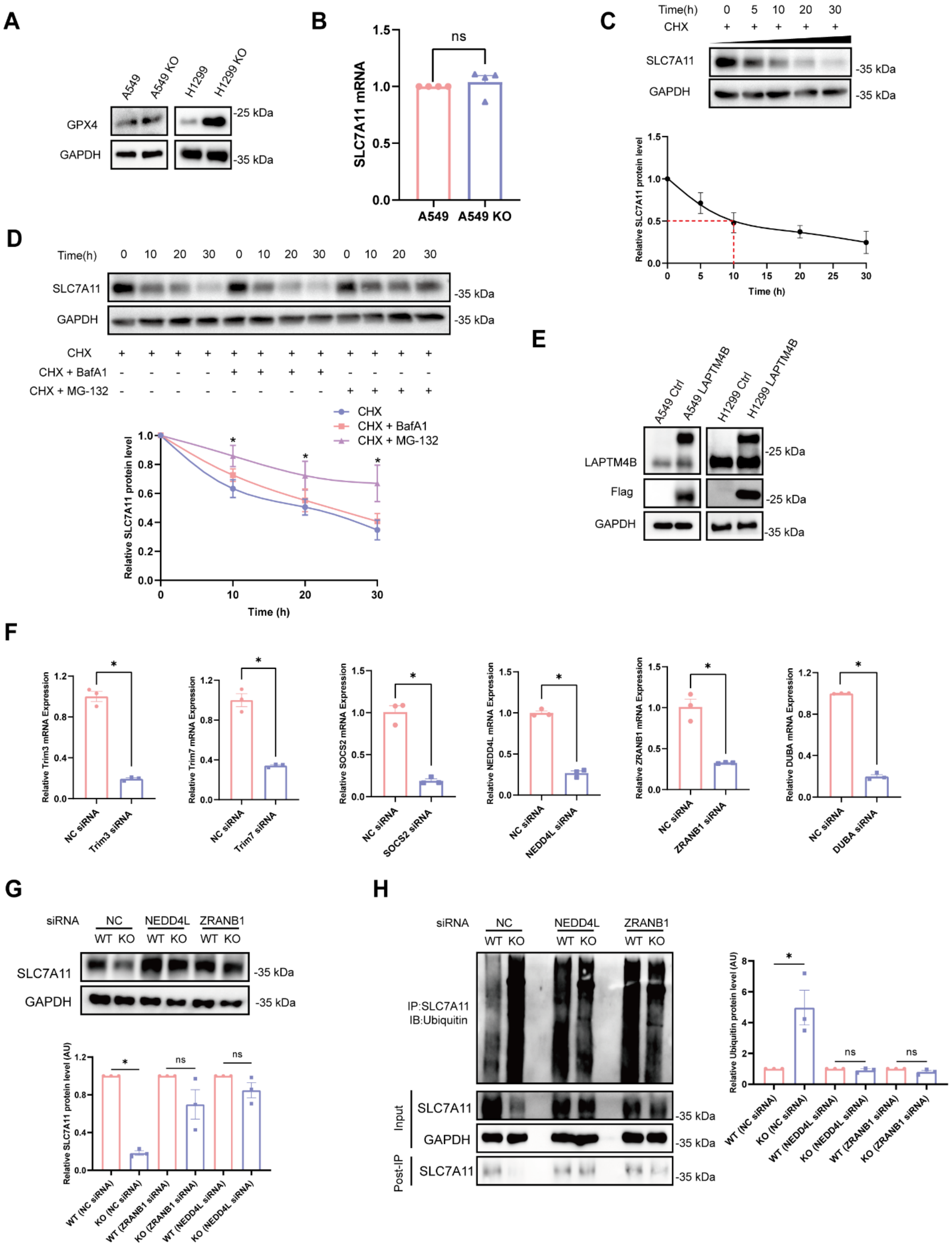

A
A549
A549 KO
H1299
H1299 KO
GPX4
GAPDH
-25 kDa
-35 kDa
B
ns
SLC7A11 mRNA
1.5
1.0
0.5
0.0
A549
A549 KO
C
Time(h) 0 5 10 20 30
CHX + + + + +
SLC7A11
GAPDH
-35 kDa
-35 kDa
Relative SLC7A11 protein level
Time (h)
D
Time(h) 0 10 20 30 0 10 20 30 0 10 20 30
SLC7A11
GAPDH
-35 kDa
-35 kDa
CHX
CHX + BafA1
CHX + MG-132
Relative SLC7A11 protein level
Time (h)
E
A549 Ctrl
A549 LAPTM4B
H1299 Ctrl
H1299 LAPTM4B
LAPTM4B
Flag
GAPDH
-25 kDa
-25 kDa
-35 kDa
F
Relative Trim3 mRNA Expression
NC siRNA
Trim3 siRNA
Relative Trim7 mRNA Expression
Trim7 siRNA
Relative SOCS2 mRNA Expression
SOCS2 siRNA
Relative NEDD4L mRNA Expression
NEDD4L siRNA
Relative ZRANB1 mRNA Expression
ZRANB1 siRNA
Relative DUBA mRNA Expression
DUBA siRNA
G
siRNA NC NEDD4L ZRANB1
WT KO WT KO WT KO
SLC7A11
GAPDH
-35 kDa
-35 kDa
Relative SLC7A11 protein level (AU)
WT (NC siRNA)
KO (NC siRNA)
WT (ZRANB1 siRNA)
KO (ZRANB1 siRNA)
WT (NEDD4L siRNA)
KO (NEDD4L siRNA)
H
siRNA NC NEDD4L ZRANB1
WT KO WT KO WT KO
IP:SLC7A11
IB:Ubiquitin
Input
SLC7A11
GAPDH
Post-IP
SLC7A11
-35 kDa
Relative Ubiquitin protein level (AU)
WT (NC siRNA)
KO (NC siRNA)
WT (NEDD4L siRNA)
KO (NEDD4L siRNA)
WT (ZRANB1 siRNA)
KO (ZRANB1 siRNA)

Supplementary Figure S4. Depletion of LAPTM4B accelerates erastin-induced ferroptosis

(A) Western blot analysis of GPX4 protein levels in LAPTM4B-depleted A549 and H1299 cells.

(B) Quantification of SLC7A11 mRNA expression in wild-type (WT) and LAPTM4B knockout (KO) cells measured by Q-PCR. Quantification of n=4 experiments, presented as mean ± SEM, data normalized to "A549".

(C) Western blot analysis of SLC7A11 protein levels in A549 cells treated with 50 µg/mL cycloheximide (CHX) for the indicated times. Upper panel: Representative experiment. Lower panel: Quantification of n=3 experiments, presented as mean ± SEM. The red dashed line indicates the time point when half of the endogenous SLC7A11 has been degraded.

(D) Western blot analysis of SLC7A11 protein levels in A549 cells treated with 1 µmol/L bafilomycin-A1 (BafA1) or 20 µmol/L MG-132, together with 50 µg/mL CHX for the indicated times. Upper panel: Representative experiment. Lower panel: Quantification of n=3 experiments, presented as mean ± SEM. * $p<0.05$.

(E) Western blot analysis of LAPTM4B expression in LAPTM4B stably expressing cells compared to control cells, using anti-LAPTM4B and anti-Flag antibodies.

(F) Measurement of the siRNAs' efficiency by Q-PCR in A549 cells.

(G) WT and LAPTM4B KO H1299 cells were transfected with the indicated siRNA, and subsequent western blotting was performed to determine SLC7A11 protein levels. Upper panel: Representative experiment. Lower panel: Quantification of n=3 experiments, presented as mean ± SEM. * $p<0.05$.

(H) WT and LAPTM4B KO H1299 cells were transfected with indicated siRNA. Immunoprecipitation of the cell lysate using SLC7A11 antibody, followed by immunoblotting with Ubiquitin antibody. Left panel: Representative experiment. Right panel: Quantification of at least three experiments, presented as mean ± SEM. * $p<0.05$.

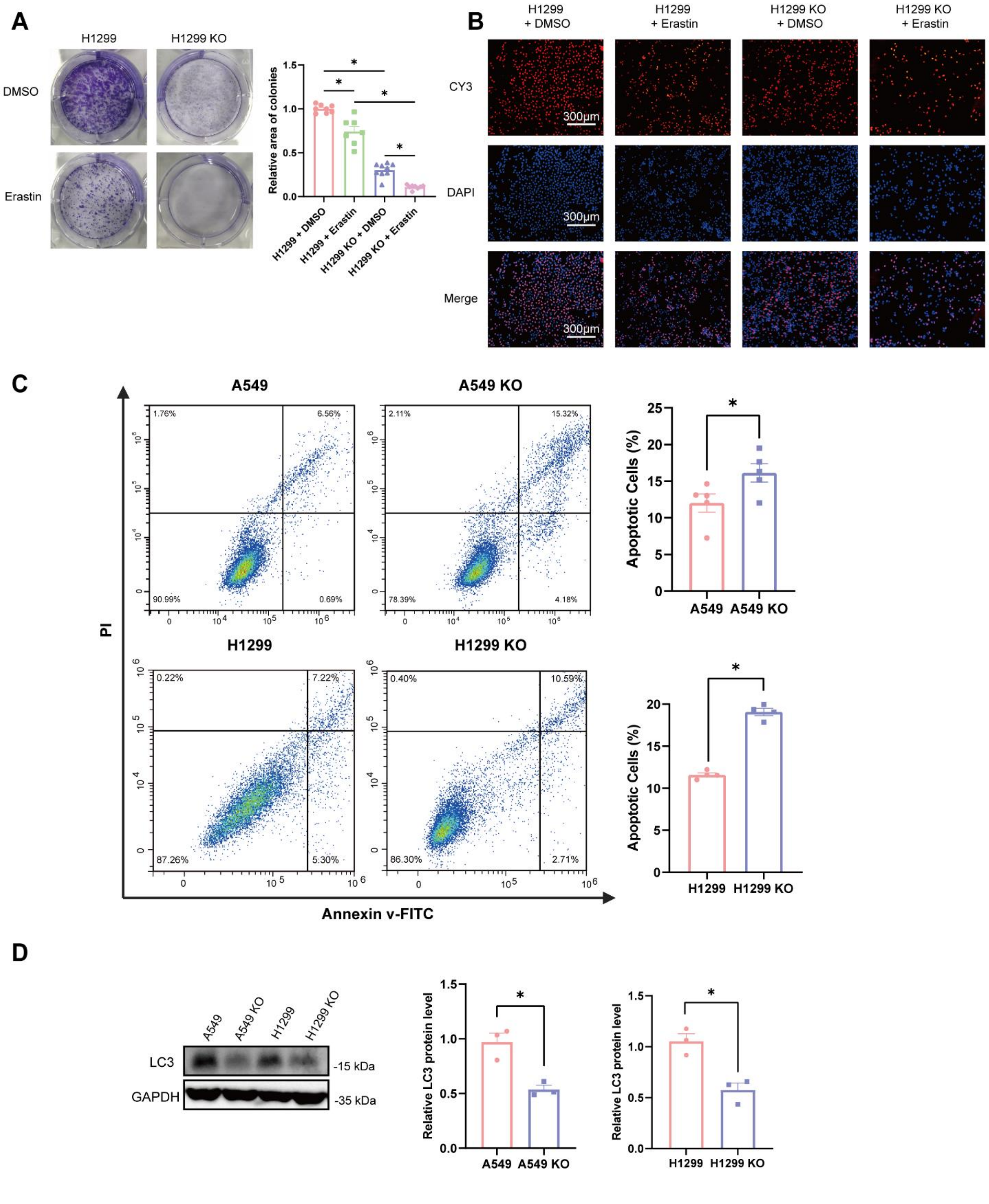
A
H1299
H1299 KO
DMSO
Erastin
Relative area of colonies
H1299 + DMSO
H1299 + Erastin
H1299 KO + DMSO
H1299 KO + Erastin
B
H1299 + DMSO
H1299 + Erastin
H1299 KO + DMSO
H1299 KO + Erastin
CY3
DAPI
Merge
300μm
C
A549
A549 KO
H1299
H1299 KO
PI
Annexin v-FITC
Apoptotic Cells (%)
D
LC3
GAPDH
-15 kDa
-35 kDa
Relative LC3 protein level

Supplementary Figure S5. LAPTM4B regulates apoptosis and autophagy in NSCLC cells

(A) 4 × 10^3 WT and LAPTM4B KO H1299 cells were seeded into a 6-well plate, treated with 5 μM erastin for 24 hours and cultured at 37°C for 10 days. Afterwards, the cells were fixed with methanol, stained with crystal violet, and subsequently imaged and quantified. Left panel: representative experiment. Right panel: quantification of n=3 experiments, mean ± SEM. *p*(H1299_DMSO, H1299_Erastin)=0.0003, *p*(H1299_DMSO, H1299 KO_DMSO)=3.895E-13, *p*(H1299 KO_DMSO, H1299 KO_Erastin)=3.537E-06, *p*(H1299_Erastin, H1299 KO_Erastin)=1.722E-08.

(B) 8 × 10^3 WT or LAPTM4B KO H1299 cells were seeded into 96-well plates. Following treatment with 5 μM erastin for 24 hours, the cells were stained with DAPI (blue) and EdU (red) to visualize the proliferative cells.

(C) Cells were collected and stained with Annexin V and PI. Left panel: representative experiment. Right panel: Quantification of at least three experiments, presented as mean ± SEM. *p*(A549, A549 KO)=0.0249, *p*(H1299, H1299 KO)=2.906E-06.

(D) Western blot analysis of LC3 protein levels in LAPTM4B-depleted A549 and H1299 cells. Left panel: representative experiment. Right panel: Quantification of n=3 experiments, mean ± SEM. *p*(A549, A549 KO)= 0.0044, *p*(H1299, H1299 KO)= 0.0047.

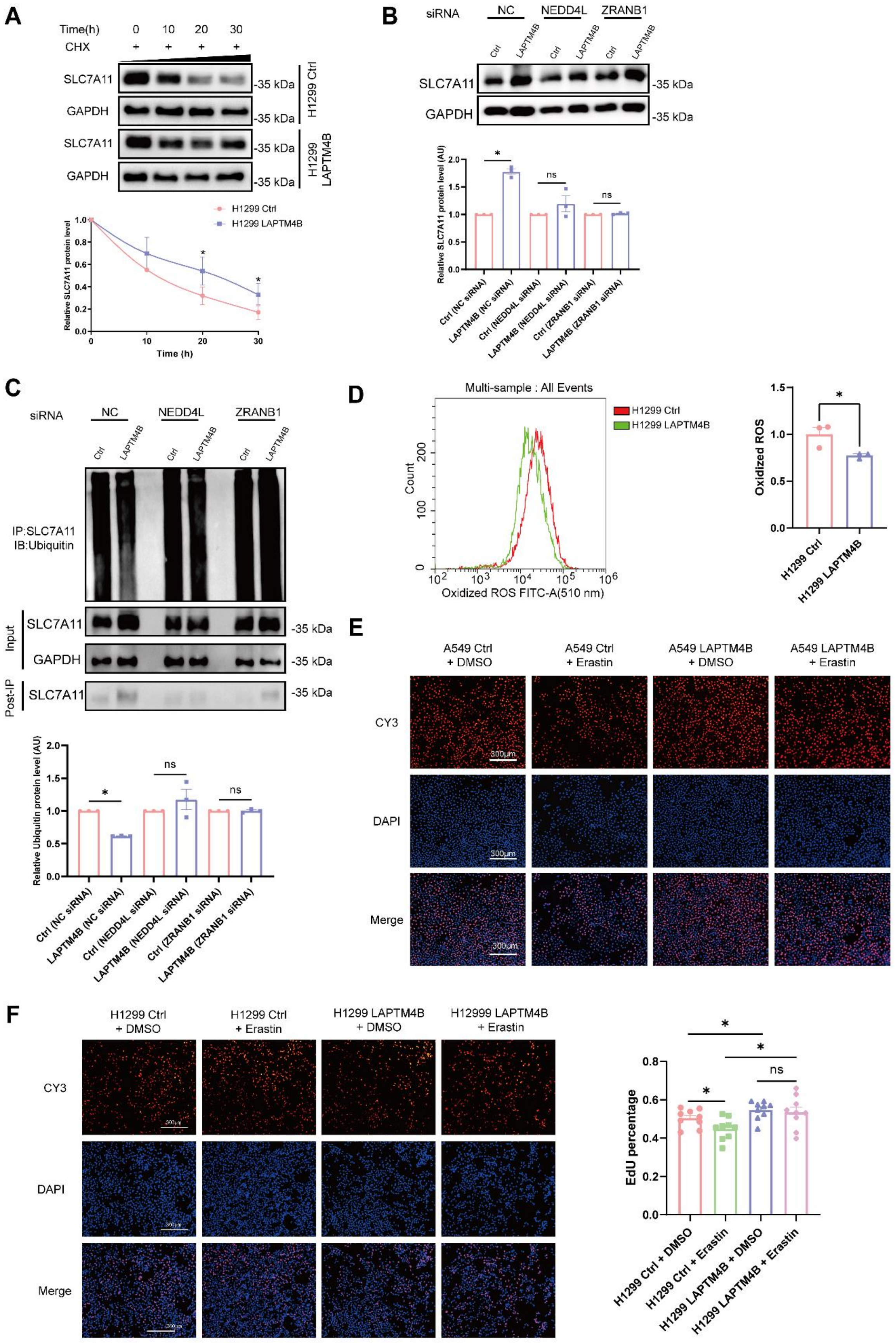
A
Time(h) 0 10 20 30
CHX + + + +
SLC7A11
GAPDH
-35 kDa
H1299 Ctrl
H1299 LAPTM4B
H1299 Ctrl
H1299 LAPTM4B
Relative SLC7A11 protein level
Time (h)
B
siRNA
NC
NEDD4L
ZRANB1
Ctrl
LAPTM4B
SLC7A11
GAPDH
-35 kDa
Relative SLC7A11 protein level (AU)
ns
Ctrl (NC siRNA)
LAPTM4B (NC siRNA)
Ctrl (NEDD4L siRNA)
LAPTM4B (NEDD4L siRNA)
Ctrl (ZRANB1 siRNA)
LAPTM4B (ZRANB1 siRNA)
C
siRNA
NC
NEDD4L
ZRANB1
IP:SLC7A11
IB:Ubiquitin
Input
SLC7A11
GAPDH
Post-IP
-35 kDa
Relative Ubiquitin protein level (AU)
D
Multi-sample : All Events
H1299 Ctrl
H1299 LAPTM4B
Count
Oxidized ROS FITC-A(510 nm)
Oxidized ROS
E
A549 Ctrl + DMSO
A549 Ctrl + Erastin
A549 LAPTM4B + DMSO
A549 LAPTM4B + Erastin
CY3
DAPI
Merge
300μm
F
H1299 Ctrl + DMSO
H1299 Ctrl + Erastin
H1299 LAPTM4B + DMSO
H12999 LAPTM4B + Erastin
CY3
DAPI
Merge
EdU percentage
H1299 Ctrl + DMSO
H1299 Ctrl + Erastin
H1299 LAPTM4B + DMSO
H1299 LAPTM4B + Erastin

Supplementary Figure S6. The regulation of LAPTM4B on the SLC7A11 degradation is facilitated by NEDD4L/ZRANB1

(A) H1299 cells stably expressing LAPTM4B and control cells were treated with 50 µg/mL CHX for the indicated times, and SLC7A11 protein levels were assessed by Western blotting. Upper panel: Representative experiment. Lower panel: Quantification of n=3 experiments, mean ± SEM. * $p<0.05$.

(B) Stably expressing LAPTM4B H1299 cells and the control cells were transfected with the indicated siRNA, and subsequent western blotting were performed to determine SLC7A11 protein levels. Upper panel: Representative experiment. Lower panel: Quantification of n=3 experiments, presented as mean ± SEM. * $p<0.05$.

(C) Stably expressing LAPTM4B H1299 cells and the control cells were transfected with indicated siRNA. Immunoprecipitation of the cell lysate using SLC7A11 antibody, followed by immunoblotting with Ubiquitin antibody. Upper panel: Representative experiment. Lower panel: Quantification of at least three experiments, presented as mean ± SEM. * $p<0.05$.

(D) LAPTM4B stably expressing H1299 cells and control cells were harvested to measure lipid peroxidation. Left panel: Representative experiment. Right panel: Quantification of n=3 experiments, mean ± SEM. *p*(H1299 Ctrl, H1299 LAPTM4B)=0.0031.

(E) LAPTM4B overexpressing A549 cells and control cells were seeded at a density of 8×10^3 cells per well in 96-well plates. After treatment with 5 µM erastin for 24 hours, cells were stained with DAPI (blue) and EdU (red) to visualize proliferative cells.

(F) LAPTM4B stably expressing H1299 cells (8×10^3) and control cells were seeded in 96-well plates. After treatment with 5 µM erastin for 24 hours, cells were stained with DAPI (blue) and EdU (red) to visualize proliferative cells. Left panel: representative experiment. Right panel: quantification of n=3 experiments, mean ± SEM. *p*(H1299 Ctrl_DMSO, H1299 Ctrl_Erastin)=0.0187, *p*(H1299 Ctrl_DMSO, H1299 LAPTM4B_DMSO)=0.0342.

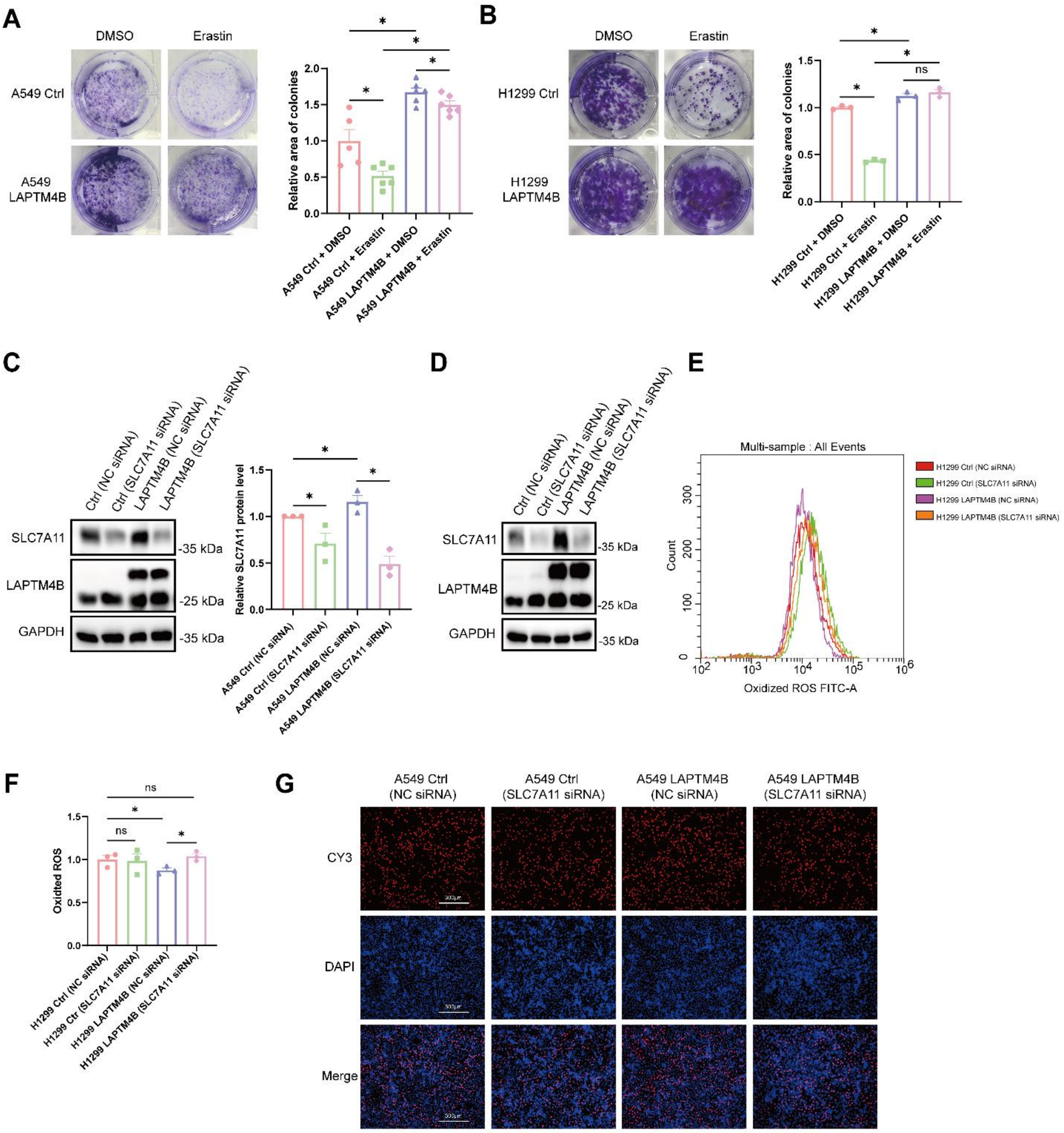

Supplementary Figure S7. The suppressive role of LAPTM4B in ferroptosis is dependent on SLC7A11

(A) LAPTM4B stably expressing A549 cells (4 × 10^3) and control cells were seeded in a 6-well plate, treated with 5 μM erastin for 24 hours, and cultured at 37°C for 10 days. Cells were fixed with methanol, stained with crystal violet, imaged, and quantified. Upper panel: representative experiment. Lower panel: quantification of n=3 experiments, mean ± SEM.

p(A549 Ctrl_DMSO, A549 Ctrl_Erastin)=0.007, *p*(A549 Ctrl_DMSO, A549 LAPTM4B_DMSO)=0.0011, *p*(A549 LAPTM4B_DMSO, A549 LAPTM4B_Erastin)=0.0286.

(B) LAPTM4B stably expressing H1299 cells (4 × 10^3) and control cells were seeded in a 6-well plate, treated with 5 μM erastin for 24 hours, and cultured at 37°C for 10 days. Cells were fixed with methanol, stained with crystal violet, imaged, and quantified. Left panel: representative experiment. Right panel: quantification of n=3 experiments, mean ± SEM. *p*(H1299 Ctrl_DMSO, H1299 Ctrl_Erastin)=1.378E-06, *p*(H1299 Ctrl_DMSO, H1299 LAPTM4B_DMSO)=0.006.

(C) LAPTM4B stably expressing A549 cells were transfected with the indicated siRNA, and subsequent western blotting was performed to determine LAPTM4B and SLC7A11 protein levels. Right panel: quantification of n=3 experiments, mean ± SEM. *p*(A549 Ctrl_NC siRNA, A549 Ctrl_SLC7A11 siRNA)=0.0299, *p*(A549 Ctrl_NC siRNA, A549 LAPTM4B_NC siRNA)=0.0439, *p*(A549 LAPTM4B_NC siRNA, A549 LAPTM4B_SLC7A11 siRNA)=0.0018.

(D) LAPTM4B stably expressing H1299 cells were transfected with indicated siRNAs, and LAPTM4B and SLC7A11 levels were determined by Western blotting.

(E) LAPTM4B stably expressing H1299 cells and control cells were transfected with SLC7A11 siRNA. 72 hours after transfection, cells were harvested to measure oxidative ROS.

(F) Quantification of ROS measurement results from (E). Quantification of n=3 experiments, mean ± SEM. *p*(H1299 Ctrl_NC siRNA, H1299 LAPTM4B_NC siRNA)=0.0403, *p*(H1299 LAPTM4B_NC siRNA, H1299 LAPTM4B_SLC7A11 siRNA)=0.0088.

(G) LAPTM4B stably expressing A549 cells and control cells were transfected with SLC7A11 siRNA and seeded in 96-well plates. 72 hours after transfection, cells were stained with DAPI (blue) and EdU (red) to visualize proliferative cells.

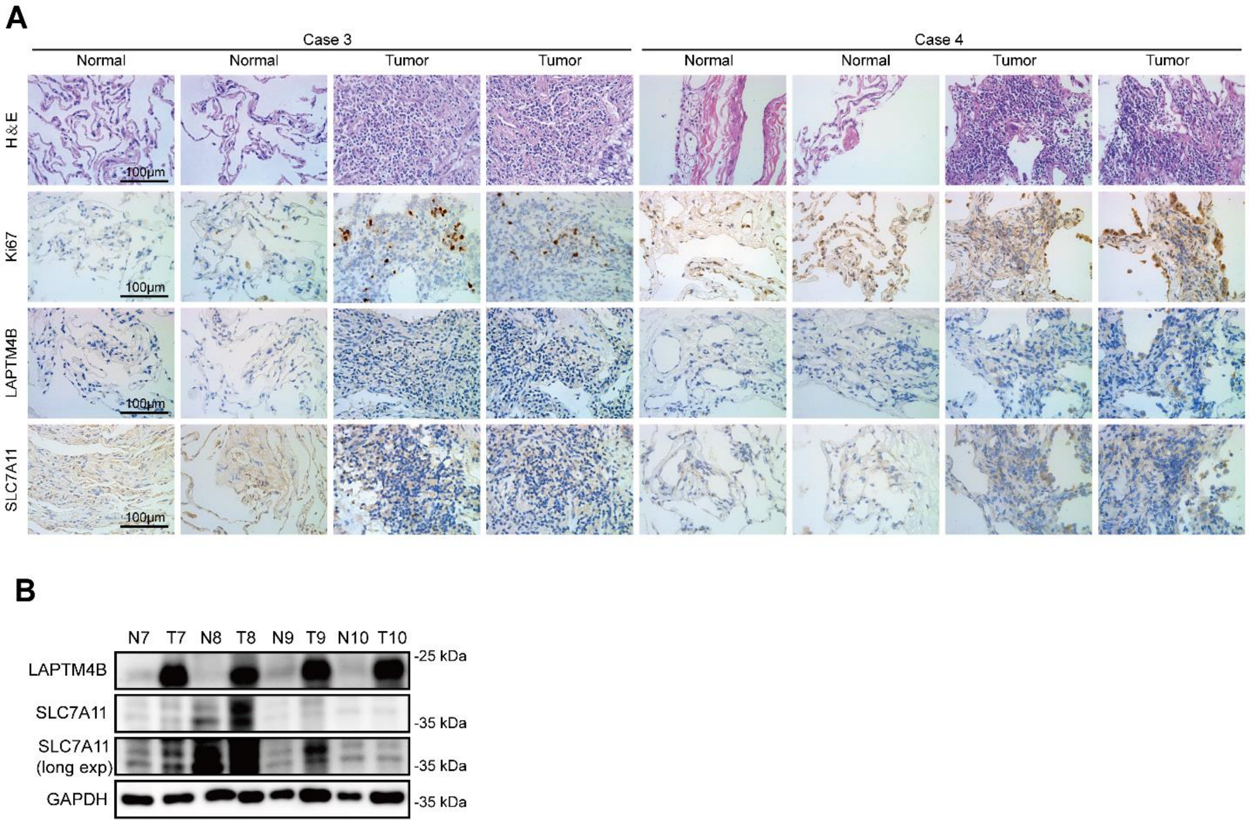

Supplementary Figure S8. Upregulation of LAPTM4B and SLC7A11 in NSCLC tumor samples

(A) Immunohistochemistry (IHC) staining of LAPTM4B and SLC7A11 protein levels in tumor tissue and adjacent normal tissues from collected NSCLC surgery samples. Representative images from H&E staining and IHC staining from different region were shown.

(B) Western blotting analysis of LAPTM4B and SLC7A11 protein levels in tumor tissue and adjacent normal tissues from collected NSCLC surgery samples.

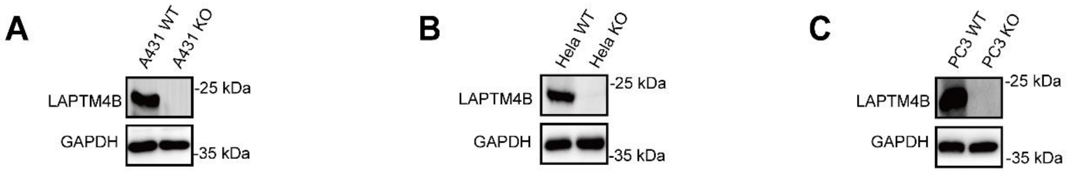

Supplementary Figure S9. Establishment of LAPTM4B knockout cells

(A) Western blot analysis showing LAPTM4B expression levels in wild-type (WT) and LAPTM4B knockout (KO) A431 cells.

(B) Western blot analysis showing LAPTM4B expression levels in WT and LAPTM4B KO Hela cells.

(C) Western blot analysis showing LAPTM4B expression levels in WT and LAPTM4B KO PC3 cells.

Supplementary Materials and Methods

Reagents, plasmids, antibodies, and siRNAs

The Erastin was from Selleck (Cat# S7242), Ferrostatin-1 from MCE (Cat# HY-100579), Liproxstatin-1 from Sigma-Aldrich (Cat# 950455-15-9), Deferoxamine mesylate from Sigma-Aldrich (Cat# 138-14-7), Z-VAD-FMK from MCE (Cat# HY-16658B), Cycloheximide from GLPBIO (Cat# GC17198), Bafilomycin A1 from GLPBIO (Cat# GC17597), MG-132 from GLPBIO (Cat# GC10383).

The mouse monoclonal anti-Flag (M2) was from Sigma-Aldrich (Cat#F1804), mouse monoclonal anti-LAPTM4B from Atlas Antibodies (Cat#AMAb91356). HA antibody from Proteintech (Cat#51064-2-AP), SLC7A11 antibody from Cell Signaling (Cat#12691S), GPX4 antibody from Proteintech (Cat#67763-1-Ig). Ki67 antibody from Servicebio (Cat#GB111499), SLC11A2 antibody from Santa Cruz (Cat#sc-166884), SLC40A1 antibody from Novus biologicals (Cat#NBP1-21502). FTL antibody from Proteintech (Cat#10727-1-AP), TFRC antibody from Proteintech (Cat#10084-2-AP), FTH1 antibody from ABclonal (Cat#A19544), LC3 antibody from Proteintech (Cat#14600-1-AP).

Protein A/G agarose beads from Santa Cruz (Cat#sc-2003), Ubiquitin antibody from Santa Cruz (Cat#sc-8017). GAPDH antibody from Proteintech (Cat#60004-1-lg). The secondary antibodies Goat Anti-Mouse IgG (H+L)-HRP (Cat#SA00001-1) and Goat Anti-Rat IgG (H+L)-HRP (Cat#SA00001-2) were from Proteintech. Cross-adsorbed Alexa Fluor 488-conjugated Goat Anti-Rabbit IgG (Cat#AS053) and Cy3 Goat Anti-Mouse IgG was from Abclonal.

The SLC7A11 siRNA (CCCTGGAGTTATGCAGCTAAT), TRIM3 siRNA (GGACTTCCATAACCATTCA), TRIM7 siRNA (GCTGAGCAGGTGTAGCAAT), SOCS2 siRNA (GAACGGCACTGTTCACCTT), NEDD4L siRNA (GGAGAATTATGTCCGTGAA), ZRANB1 siRNA (GAAGAGGCTTCTTCAATAA), DUBA siRNA (GTTGCGGGATCAGGAGAAA), and Ctrl siRNA were from RiboBio biotech company.

Western blotting

To measure the protein levels, the cells were washed with ice-cold phosphate-buffered saline (PBS) and lysed in RIPA lysis buffer (Biosharp, Cat#P0013B). The cell lysates were then boiled at 99°C for 10 minutes. Equal amounts of proteins were separated on 12% SDS-

PAGE gels and transferred onto LF-PVDF (Millipore, Cat#IPVH00010) or NC Transfer Membrane (Millipore, Cat#HATF00010). The membranes were blocked with 3% BSA in TBS containing 0.1% Tween-20 (TBST) for 1 hour at room temperature or 5% non-fat milk in TBST, and then incubated with primary antibodies overnight at 4°C. After four washes with TBST, the membranes were incubated with secondary antibodies for 1 hour at room temperature. Subsequently, the membranes were washed and incubated with Ultrasensitive ECL Chemiluminescent Substrate (Biosharp, Cat#BL523B), followed by imaging using ChemiCapture Imaging System (Clinx, Cat#6000Exp). The quantification of protein levels was performed by normalizing to an internal control protein using ImageJ software version 1.53C (NIH, Bethesda, MD; http://imagej.nih.gov/ij).

Data preprocessing and filtering for Metabolomics analysis

The raw mass spectrometry (MS) data underwent comprehensive processing using MS-DIAL software, encompassing peak alignment, retention time correction, and peak area extraction. Metabolite identification was achieved through the integration of accurate mass analysis (with a mass tolerance of less than 10 ppm) and MS/MS data comparison (with a mass tolerance of less than 0.02 Da) against established databases such as HMDB, massbank, and other publicly available repositories, as well as our own curated metabolite standard library.

To ensure data quality and reliability, only variables within the extracted-ion features that exhibited non-zero measurement values in at least one group, accounting for more than 50% of the total measurements, were retained for further analysis. This selection criterion aimed to focus on metabolites that exhibited consistent and meaningful variations across the experimental groups, ensuring robust statistical analysis and interpretation of the results.

Multivariate statistical analysis for Metabolomics analysis

All multivariate data analyses and modeling were conducted using R (version 4.0.3) and appropriate R packages, adhering to established academic practices. Prior to analysis, the data underwent mean-centering and Pareto scaling to account for variations in scale and to enhance comparability between variables. Several modeling techniques were employed in this study, including principal component analysis (PCA), orthogonal partial least squares

discriminant analysis (PLS-DA), and partial least squares discriminant analysis (OPLS-DA). These techniques were utilized to explore the underlying patterns and discriminatory features within the dataset.

To ensure the robustness and reliability of the models, permutation tests were applied to assess potential overfitting. The performance of the models was evaluated using various metrics. Descriptive performance was assessed using cumulative R2X (with a perfect model achieving R2X (cumulative) = 1) and cumulative R2Y (with a perfect model achieving R2Y (cumulative) = 1). Prediction performance was measured by cumulative Q2 (with a perfect model achieving Q2 (cumulative) = 1) and validated using a permutation test (n = 200). An ideal model should have R2 and Q2 values at the Y-axis intercept lower than those of the non-permuted model, indicating reliable predictive power.

OPLS-DA analysis enabled the identification of metabolites contributing to class discrimination through the variable importance on projection (VIP) scores. The VIP score reflects the relative contribution of each variable in discriminating among the different sample classes. It is calculated as a weighted sum of squares of PLS weights for each variable. Typically, a VIP value over 1 is considered significant, as values above this threshold indicate a considerable discriminatory ability. Hence, variables with high VIP scores are potential biomarkers and are worthy of consideration for further investigation.

The identification of metabolites with discriminatory potential was achieved by employing a rigorous statistical approach. Specifically, variable influence on projection (VIP) values obtained from the OPLS-DA model, in combination with a two-tailed Student's t-test (p value), were used at the univariate analysis level on normalized raw data. To determine statistical significance, the p value was calculated using one-way analysis of variance (ANOVA) for multiple groups analysis. Metabolites exhibiting VIP values greater than 1.0 and a p value less than 0.05 were considered to be statistically significant, indicating their potential importance in distinguishing among the studied groups.

To further assess the magnitude of change, fold change was calculated as the logarithm of the average mass response (area) ratio between two arbitrary classes. This measure provided insights into the relative differences in metabolite abundance between the compared classes. Moreover, the identified differential metabolites were utilized to perform cluster analyses using the R package. This approach facilitated the exploration of potential

patterns and relationships among the metabolites, aiding in the interpretation of their biological relevance and potential functional connections.

Transmission electron microscope measurement

Transmission electron microscope (TEM) was conducted by HITACHI company. Briefly, cells were washed with PBS and collected by centrifugation, the precipitation was re-suspended in TEM fixative and subsequently fixed at 4℃ for preservation. For agarose pre-embedding, the fixed cells underwent centrifugation. After discarding the supernatant, 0.1 M phosphate buffer (PB) with a pH of 7.4 was added to the tube, and the precipitation was re-suspended and washed in PB for 3 minutes. This washing step was repeated three times. Subsequently, a 1% agarose solution was prepared by heating and dissolving in advance. The cooled agarose solution was added to the EP tube, and before agarose solidification, the precipitation was carefully suspended and wrapped in the agarose. To further prepare the agarose blocks with samples, they were post-fixed with 1% OsO4 in 0.1 M PB (pH 7.4) for 2 hours at room temperature while being shielded from light. Following the removal of OsO4, the tissues were rinsed three times in 0.1 M PB (pH 7.4), with each rinse lasting 15 minutes. The subsequent steps involved dehydrating the samples at room temperature, followed by resin penetration and embedding. Polymerization was then performed to ensure sample stability. Ultrathin sections were obtained, and staining techniques were applied. Finally, the samples were observed under TEM, and images were captured using cuprum grids. The ultrastructure of mitochondria was analyzed and quantified using ImageJ as described previously.

Cell counting kit-8 (CCK8) assay and colony formation experiment

The current study employed a CCK-8 kit (Dojindo, Japan) to assess cellular proliferative capacities under various treatment conditions. For this purpose, 4×10^3 cells were seeded into 96-well plates containing 100 μl of culture medium and exposed to 5 μM erastin for 24, 48, and 72 hours. Subsequently, 10 μl of CCK-8 reagent was introduced into each well, and the plates were subjected to a 2 hours incubation period prior to measuring absorbance at 450 nm using a microplate reader (Spark, TECAN). This process was repeated after 24 and 48 hours of incubation. To conduct the clone formation assay, 4×10^3 WT or KO cells were seeded into a 6-well plate, treated with 2.5-5 μM erastin for 24 hours, and cultured at 37 ^oC

for 12-14 days. Afterwards, the cells were fixed with methanol, stained with crystal violet, and subsequently imaged.

EdU assay

The current study utilized an EdU kit (Rib bio, China, Cat#C10310) to determine cellular proliferation. The cells in the growth phase were obtained at a density of 8 × 10^3 cells and were seeded into 96-well plates. Following treatment with 1-5 μM erastin for 24 hours, the EdU solution was diluted using cell complete medium at a ratio of 1000:1 to prepare an appropriate quantity of 50 μM EdU medium. Subsequently, 100 μL of the 50 μM EdU medium was added to each well and incubated for 2 hours. Afterward, each well was subjected to a 30 minutes incubation with 4% paraformaldehyde-containing PBS at room temperature to fix the cells. 50 μL of 2 mg/mL glycine was added to each well, and the decolorizing shaker was incubated for 5 minutes before 100 μL of 0.5% TritonX-100 was added to each well and the decolorizing shaker was incubated for 10 minutes with 0.5% TritonX-100 in PBS. Then, 100 μL of 1X Apollo® staining solution was added and incubated for 30 minutes at room temperature on a decolorizing shaker, while being protected from light. Each well was washed by adding 100 μL of 0.5% TritonX-100 in PBS decolorizing shaker 2~3 times for 10 minutes each time. Furthermore, the DNA was stained with a 1X Hoechst33342 or DAPI solution (100:1) while being protected from light. The staining process was carried out by incubating for 30 minutes at room temperature on a decolorizing shaker. Finally, 100 μL of PBS was added, and inverted fluorescence microscope images were captured by the inverted fluorescence microscope (Zeiss, Axio Observer 3).

Measurement of cell apoptosis and PI assay

The Cell Apoptosis Kit (BestBio, Cat#BB-4101) was employed to measure the apoptosis, according to the instructions from manufacturer. After 24 hours of cell attachment, the cells were digested with EDTA-free trypsin and washed twice with pre-cooled PBS, then resuspended in 400 μL of 1x Annexin-V conjugate solution. 5 μL of Annexin-V-FITC staining solution was added and the cells were incubated for 15 minutes at 2-8°C in the dark. Subsequently, 5 μL of PI staining solution was added and the cells were again incubated for 5 minutes at 2-8°C in the dark. At least 10,000 cells were collected and analyzed using a flow cytometer (Beckman coulter, USA).

For the PI assay, cells were treated with erastin or other inhibitors for 24 hours, digested with trypsin, washed twice with pre-cooled PBS and resuspended with 400 µl PBS. Then, 5 µl of PI staining solution was added to the cell suspension and incubated for 5 minutes at 2-8 °C in a dark environment. At least 10,000 cells were collected and analyzed using a flow cytometer (Beckman coulter, USA).

Analysis of SLC7A11 ubiquitination

Cells were cultured on 60-mm dishes and subsequently subjected to scraping using RIPA lysis buffer (Beyotime, Cat# P0013) containing a protease inhibitor cocktail (MCE, Cat#HY-K0010). Prior to harvesting, the cells were treated with 20 µM MG-132 for 12 hours. The resulting lysates were clarified by centrifugation at 12,000 x g for 10 minutes at 4°C. Equal amounts of the cleared cell lysates were then incubated with 2 µg of primary SLC7A11 antibody (Cell Signaling, Cat#12691S) for 8 hours at 4°C on a rocker platform. Subsequently, 20 µl of resuspended protein A/G agarose beads (Santa Cruz, Cat#SC-2003) were added and the mixture was incubated overnight at 4°C. The immunoprecipitates were enriched through centrifugation and subjected to two washes of 10 minutes each using 1 ml RIPA. The resulting supernatant was aspirated and discarded, while the pellet was resuspended in 40 µl of 1X Sample Buffer and subsequently boiled at 99°C for 10 minutes. The precipitated proteins were then analyzed using western blotting to quantify SLC7A11 ubiquitination.

Hematoxylin and Eosin (H&E) staining and Immunohistochemistry (IHC) staining

Paraffin-embedded tissue samples were prepared with 3 µm sections for subsequent experimental procedures. Hematoxylin and eosin (HE) staining was performed using an Autostainer (Leica, Cat#CV5030) for automated processing. Hematoxylin was employed to stain the cell nuclei, while eosin was used to stain the cytoplasm.

For immunohistochemistry (IHC) experiments, tissue sections were oven-dried and subjected to dewaxing with xylene, followed by rehydration using a gradient of anhydrous ethanol. Antigen retrieval was accomplished by boiling the sections in a sodium citrate repair solution for 2 minutes, followed by rinsing with H_2O until they reached room temperature. Subsequently, the sections were blocked with 0.3% H_2O_2 for 10 minutes and incubated with

an anti-LAPTM4B monoclonal antibody (Atlas Antibodies, Cat#AMAb91356, 1:600) at room temperature for 2.5 hours. After washing the sections with PBS, a secondary HRP-conjugated antibody (MXB, Cat#KIT-5010) was applied and incubated for 30 minutes at room temperature. Finally, the sections were stained for 2 minutes using the DAB development kit (Proteintech, Cat#PR30010) and counterstained with hematoxylin for 10 seconds. The slices were then dehydrated using a gradient of anhydrous ethanol and sealed with neutral resin gel.

For IHC analysis, ten random fields of view per sample were captured using microscopy (Leica, Cat#DM2500) and quantified using the "IHC Profiler" in ImageJ software version 1.53C (NIH, Bethesda, MD; http://imagej.nih.gov/ij). The IHC signal was quantified by normalizing it to the total cell number per image. All H&E and IHC images were evaluated by two independent pathologists.

Immunofluorescence staining and confocal microscopy

Immunofluorescence staining was performed following established procedures. To begin, cells were fixed in coverslips using 4% paraformaldehyde in phosphate-buffered saline (PBS) for 20 minutes at room temperature. Subsequently, cells were quenched with 50 mM NH_4Cl for 10 minutes at room temperature. The coverslips were then washed with PBS and the cells were permeabilized with 0.1% Triton X-100 in PBS for 10 minutes. Following this, cells were blocked using 10% fetal bovine serum (FBS) in PBS for 30 minutes. The coverslips were then incubated with primary antibodies, namely anti-LAPTM4B (Atlas Antibodies, Cat#AMAb91356, diluted 1:200) and anti-SLC7A11 (Proteintech Cat#26864-1-AP, diluted 1:100) at 37°C for 45 minutes. After incubation, the cells were washed with PBS and incubated with the appropriate secondary antibody (Abclonal, Alexa Fluor 488-conjugated Goat Anti-Rabbit IgG (Cat#AS053) and Cy3 Goat Anti-Mouse IgG Cat#AS008,1:200). for 45 minutes at 37°C. Finally, the coverslips were washed with PBS, rinsed in MQ-H_2O, and mounted on microscope slides using the Anti-fluorescence quenching encapsulant (Biosharp, Cat#BL701A). Images were acquired using a Zeiss confocal microscope and subsequently analyzed using ImageJ.
