# Supplementary material for: LAPTM4B counteracts ferroptosis via suppressing the ubiquitin-proteasome degradation of SLC7A11 in non-small cell lung cancer
Source: Cell Death Dis. 2024 Jun 20;15(6):436. doi: 10.1038/s41419-024-06836-x (PMC11190201; doi:10.1038/s41419-024-06836-x)
Supplement: Supplementary file 6 — Supplementary Table S5 [file 41419_2024_6836_MOESM6_ESM.docx]

**Supplementary Table S5**

| **mRNA expression analysis**  *LAPTM4B*  *SLC7A11*  *GAPDH*  *FTL*  *FTH1*  *SLC40A1*  *TFRC*  *SLC11A2*  *Trim3*  *Trim7*  *SOCS2*  *NEDD4L*  *ZRANB1*  *DUBA* | **Forward primers (5'-3');**  **Reverse primers (5'-3')**  CCTGGATCATCCCATTCTTCTGT  AATTAGGAGGCAGTTGCCGTATG  GCACGCCCTTAGGAGAGATG  TCGCTGTGAAGGAAAAAGCAC  GAAGGTGAAGGTCGGAGTC  GAAGATGGTGATGGGATTTC  CCATGAGCTCCCAGATTCGT  TTCCAGAGCCACATCATCGC  GTTCTTCGCCGAGAGTCGTC  CCACATCATCGCGGTCAAAG  AGAAAATCCCTGGGCCCCTT  AAGTGCCACATCCGATCTCC  ATCGGTTGGTGCCACTGAATGG  ACAACAGTGGGCTGGCAGAAAC  AGCTCCACCATGACAGGAACCT  TGGCAATAGAGCGAGTCAGAACC  GCCAATGGACAAGCAGTTC  CGGCATACTGGACAGGATA  GCTCGGGGTTGAGATCACC  CCAGGCACATTGCTACACCT  TTAAAAGAGGCACCAGAAGGAAC  AGTCGATCAGATGAACCACAC  TCTGGAAGGCTGTGCTAC  TCTGGGCAGTTTCTCAGG  CTAGTGCAAGACCAAGGGTG  ACACATCTTTTAGCCTTGGCCC  CAGGCTACAACAGTGAGGACGA  GAAGCCCTTCTTGTCTCGTAGG |
| --- | --- |
