# Supplementary material for: LAPTM4B counteracts ferroptosis via suppressing the ubiquitin-proteasome degradation of SLC7A11 in non-small cell lung cancer
Source: Cell Death Dis. 2024 Jun 20;15(6):436. doi: 10.1038/s41419-024-06836-x (PMC11190201; doi:10.1038/s41419-024-06836-x)

Figure 3

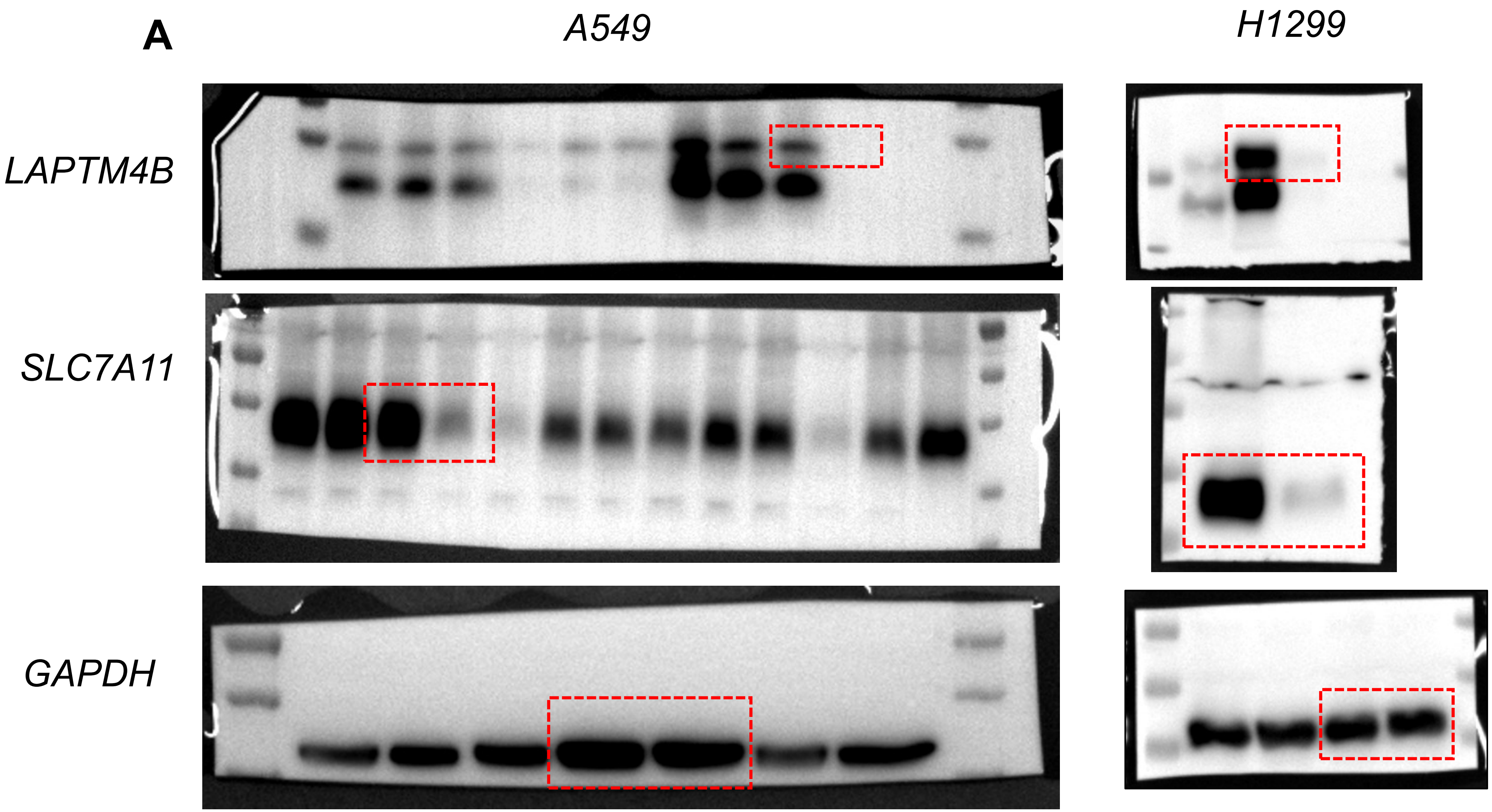

Figure 3

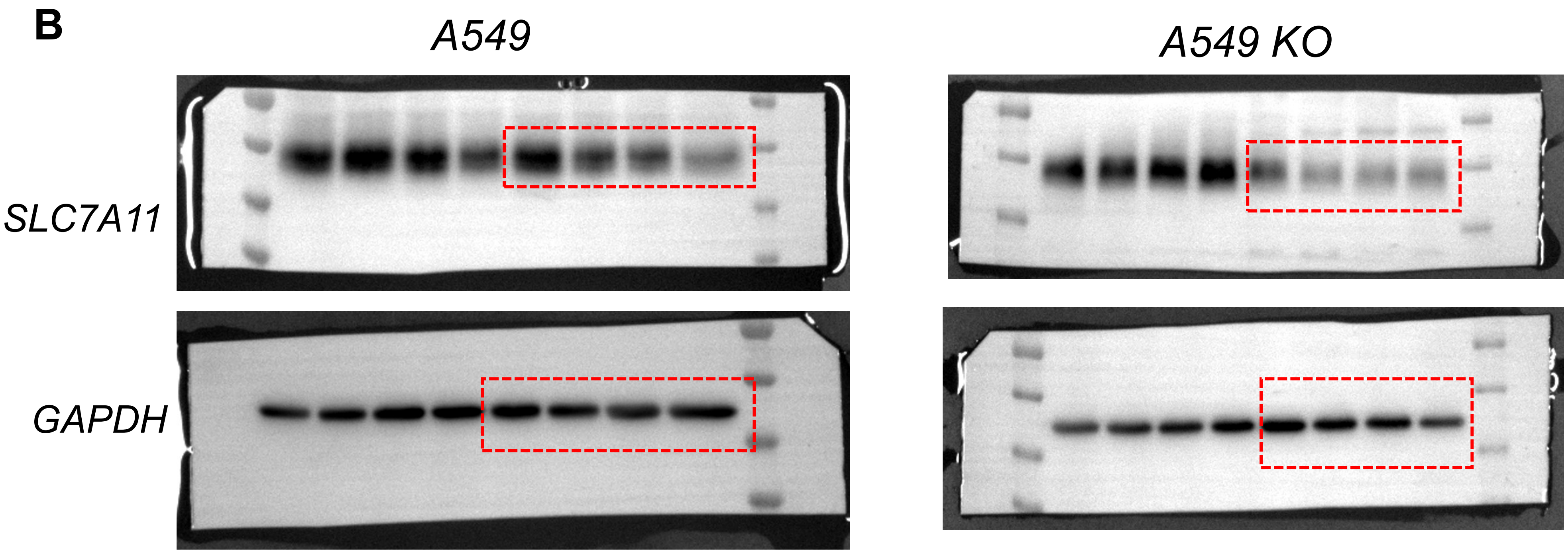

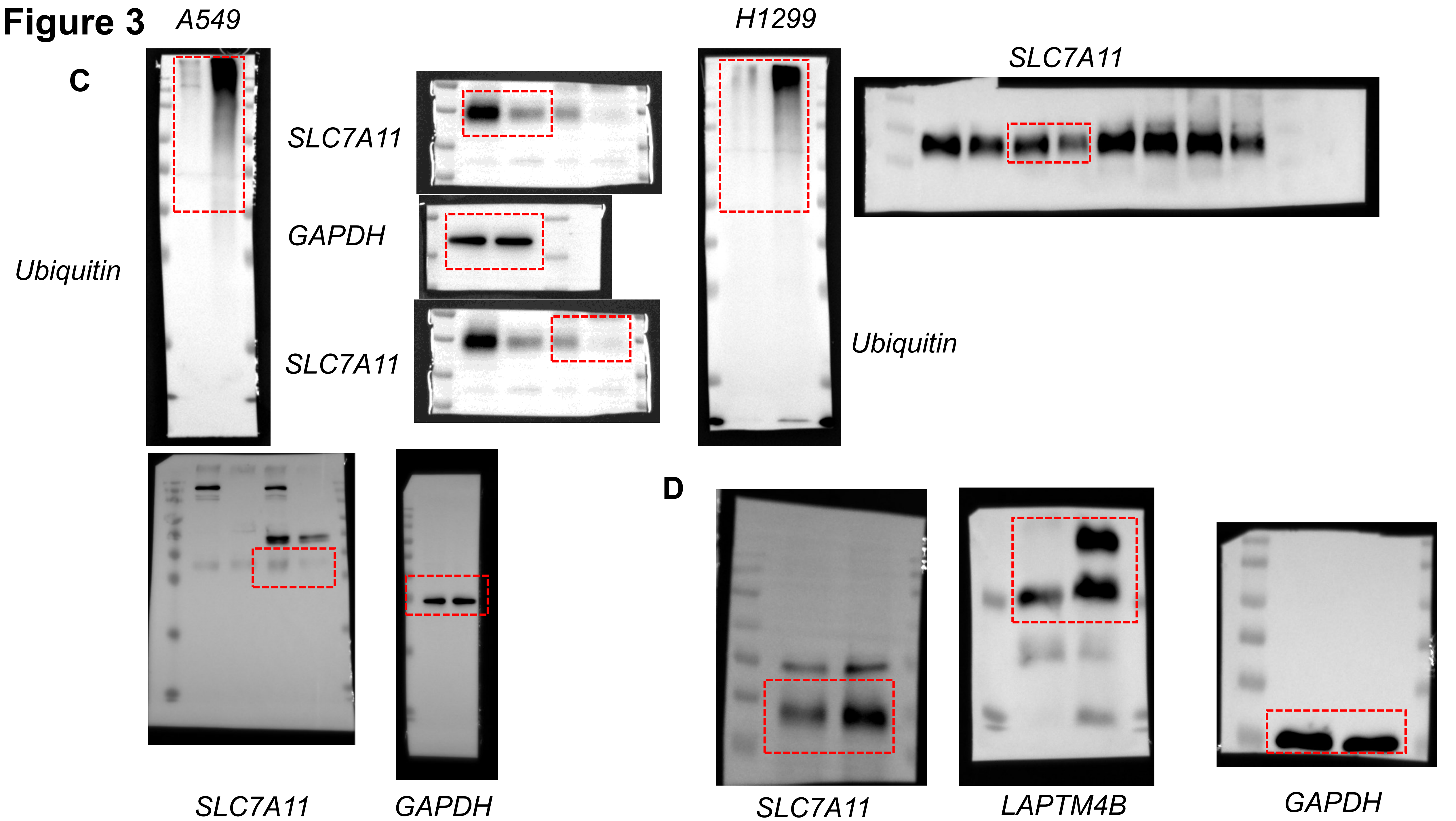
Figure 3
C
A549
Ubiquitin
SLC7A11
GAPDH
SLC7A11
SLC7A11
GAPDH
H1299
SLC7A11
Ubiquitin
D
SLC7A11
LAPTM4B
GAPDH

Figure 3

F

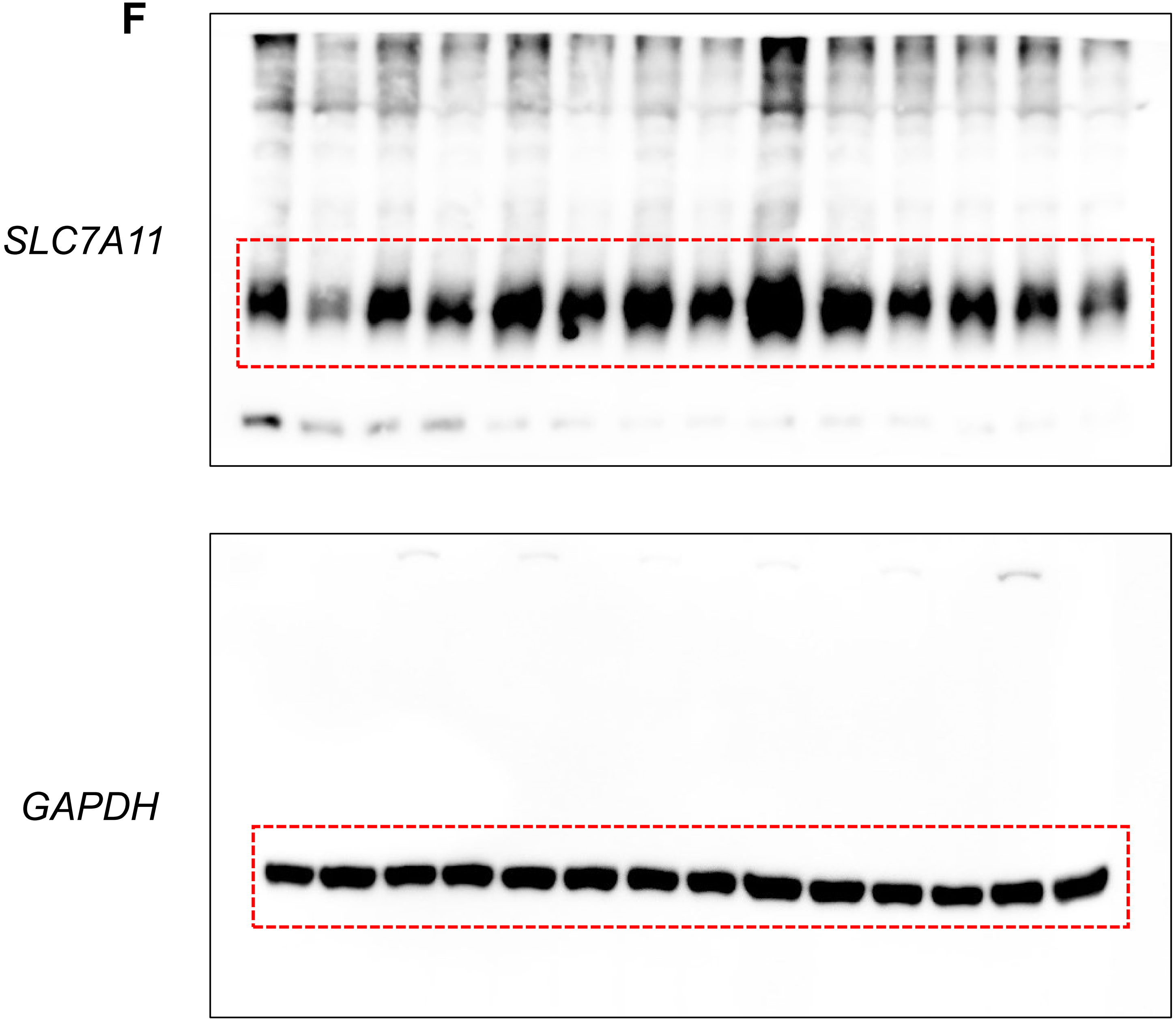

Figure 3

G

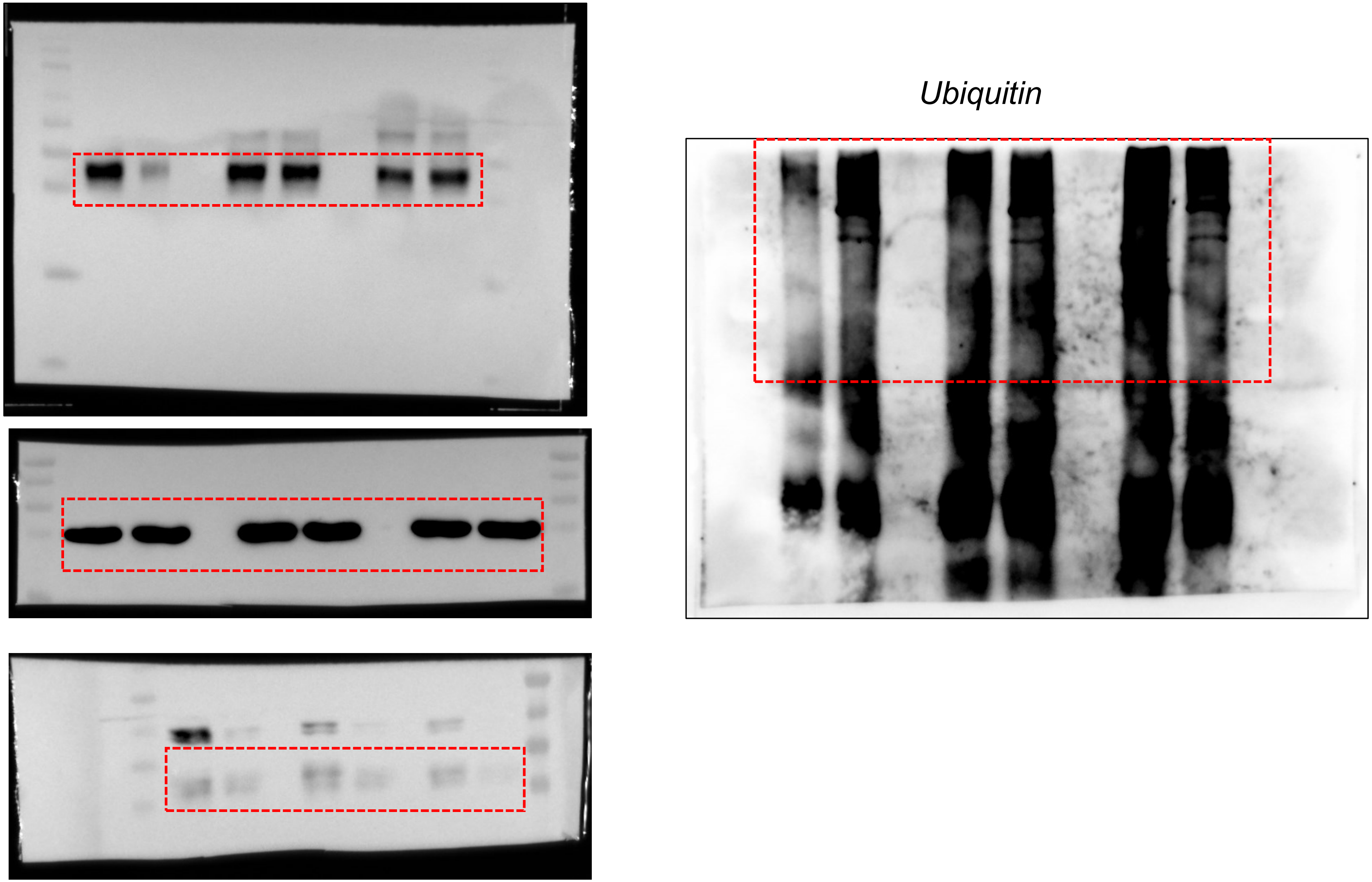

Figure 4

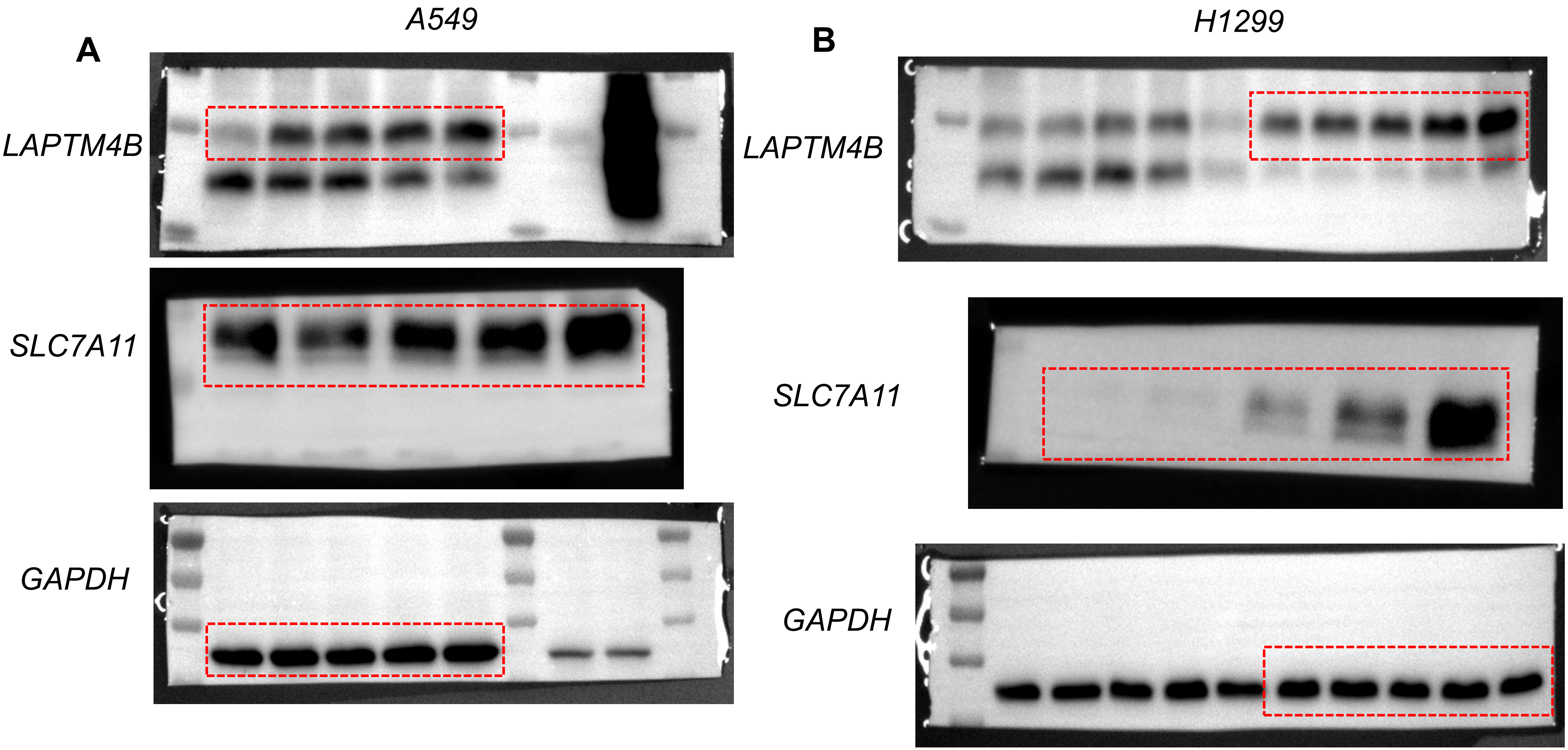

Figure 5

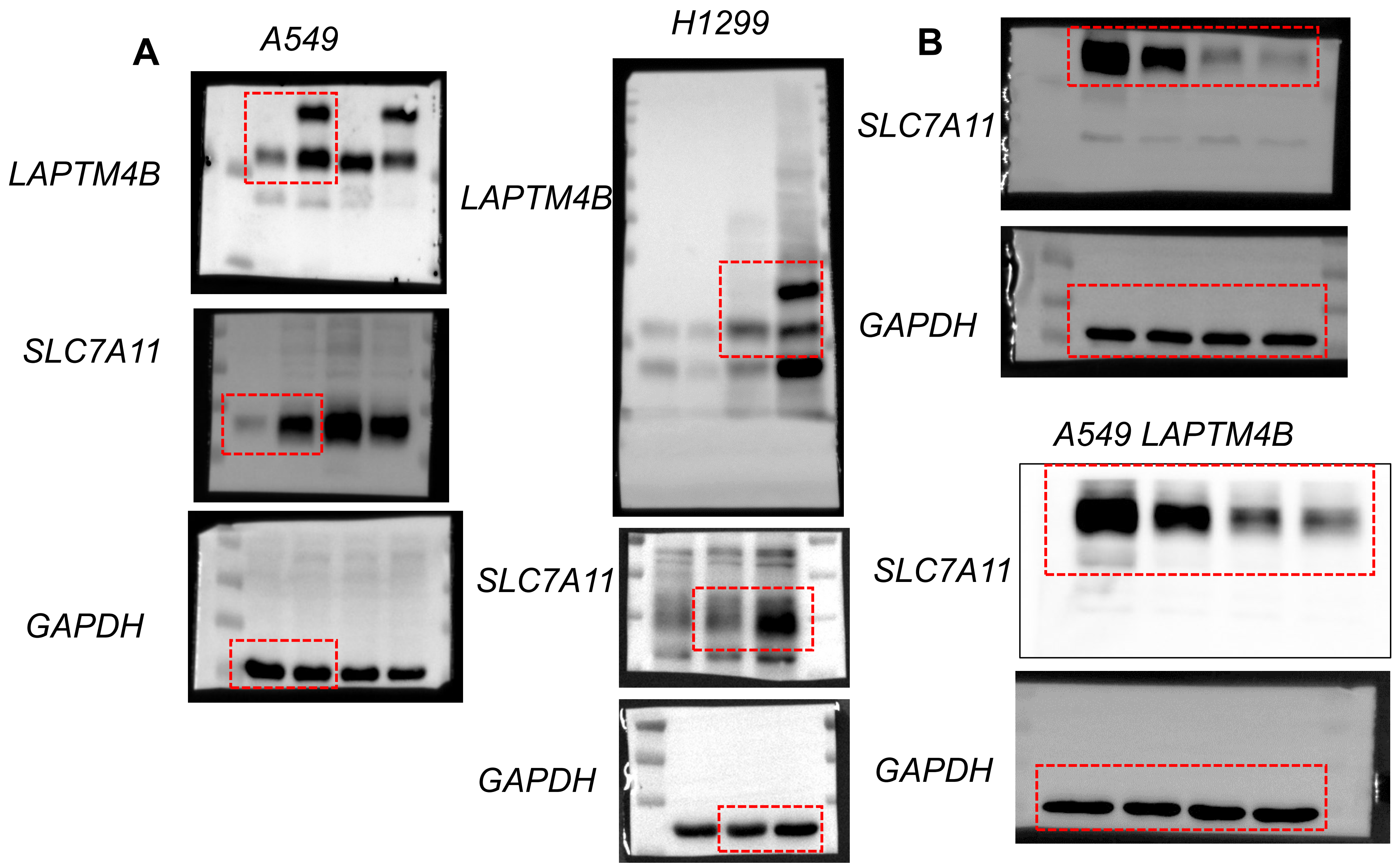
A
A549
LAPTM4B
SLC7A11
GAPDH
H1299
LAPTM4B
SLC7A11
GAPDH
B
A549 Ctrl
SLC7A11
GAPDH
A549 LAPTM4B
SLC7A11
GAPDH

Figure 5

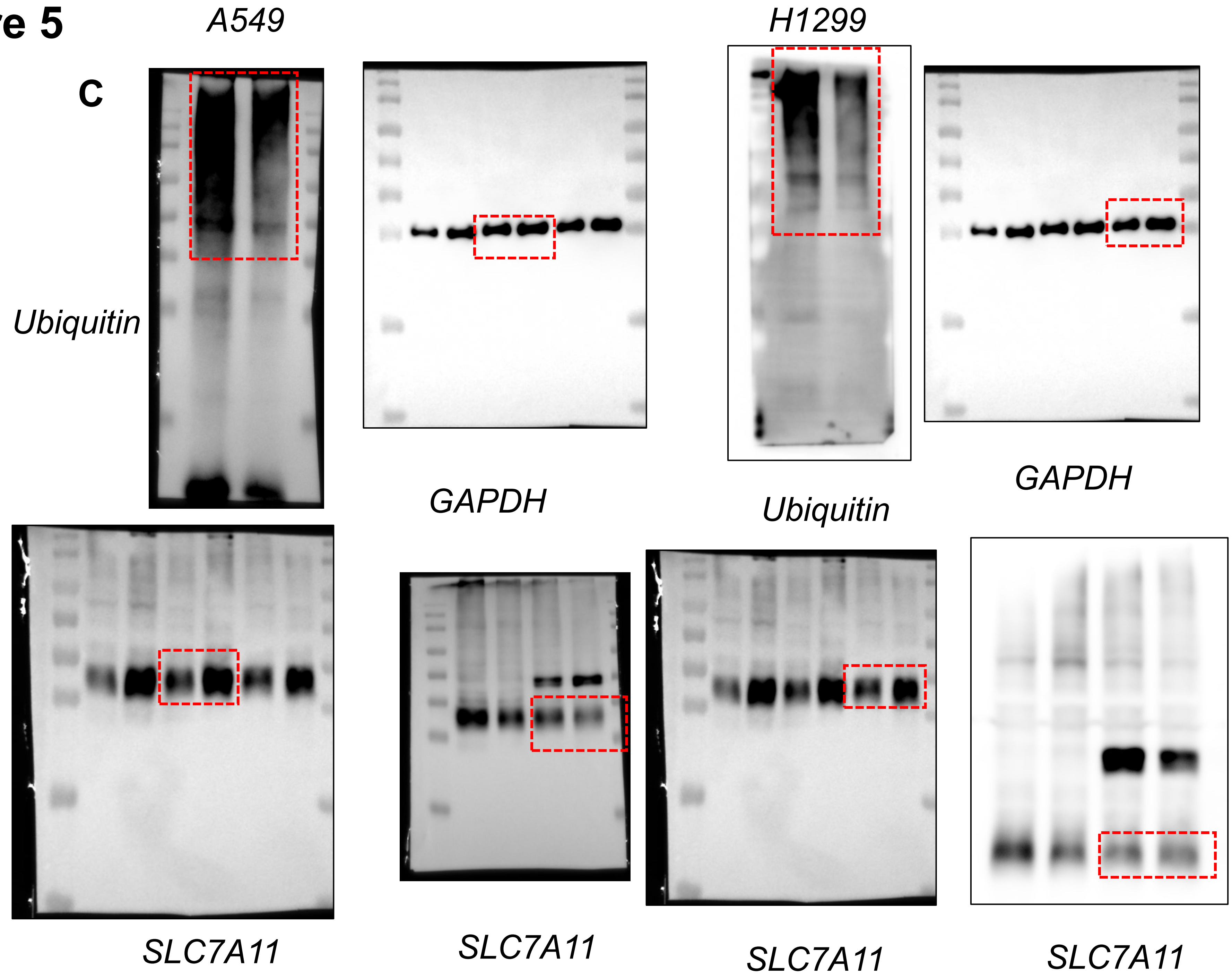

C
A549
H1299
Ubiquitin
GAPDH
Ubiquitin
GAPDH
SLC7A11
SLC7A11
SLC7A11
SLC7A11

Figure 5

D

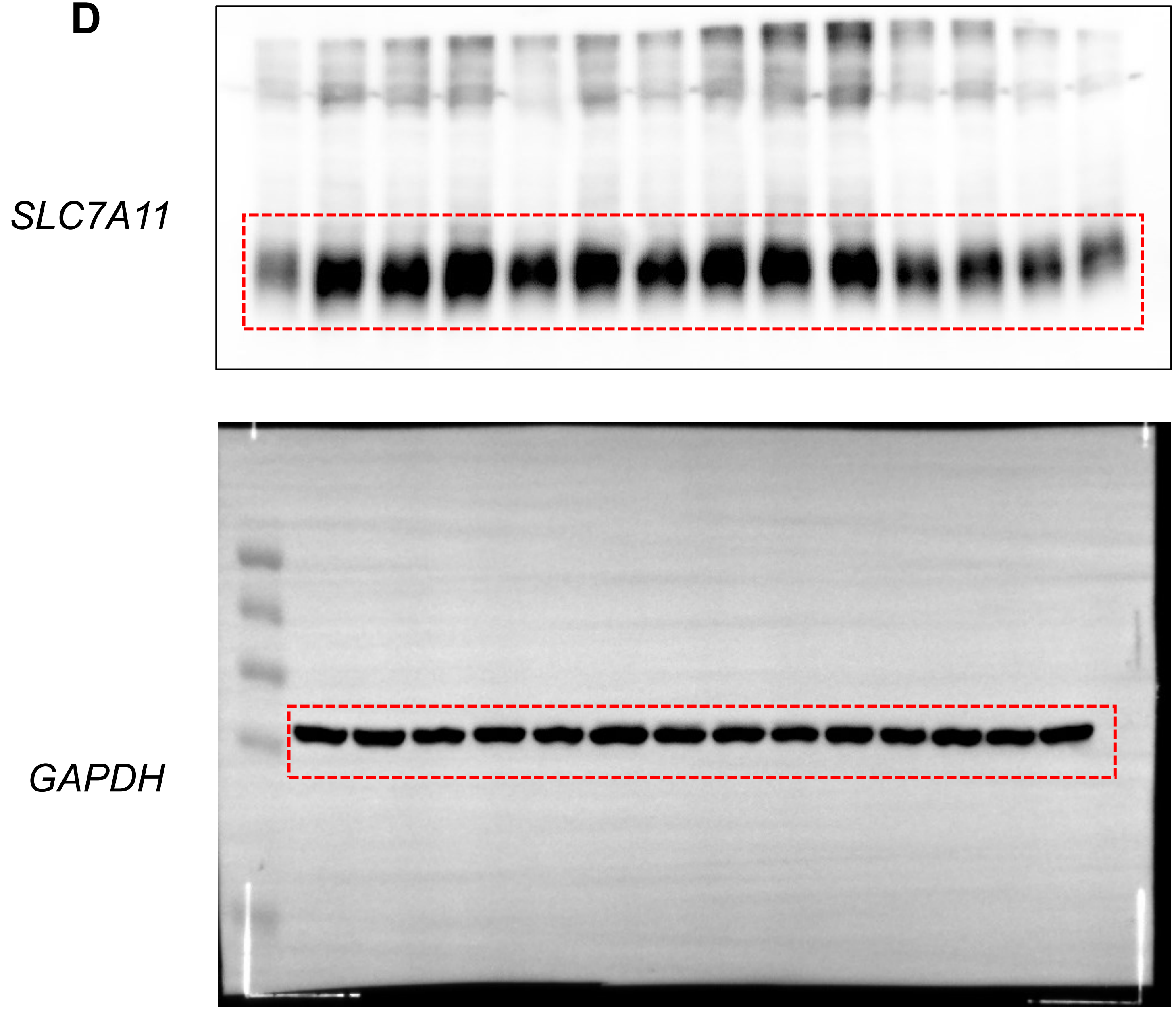

Figure 5

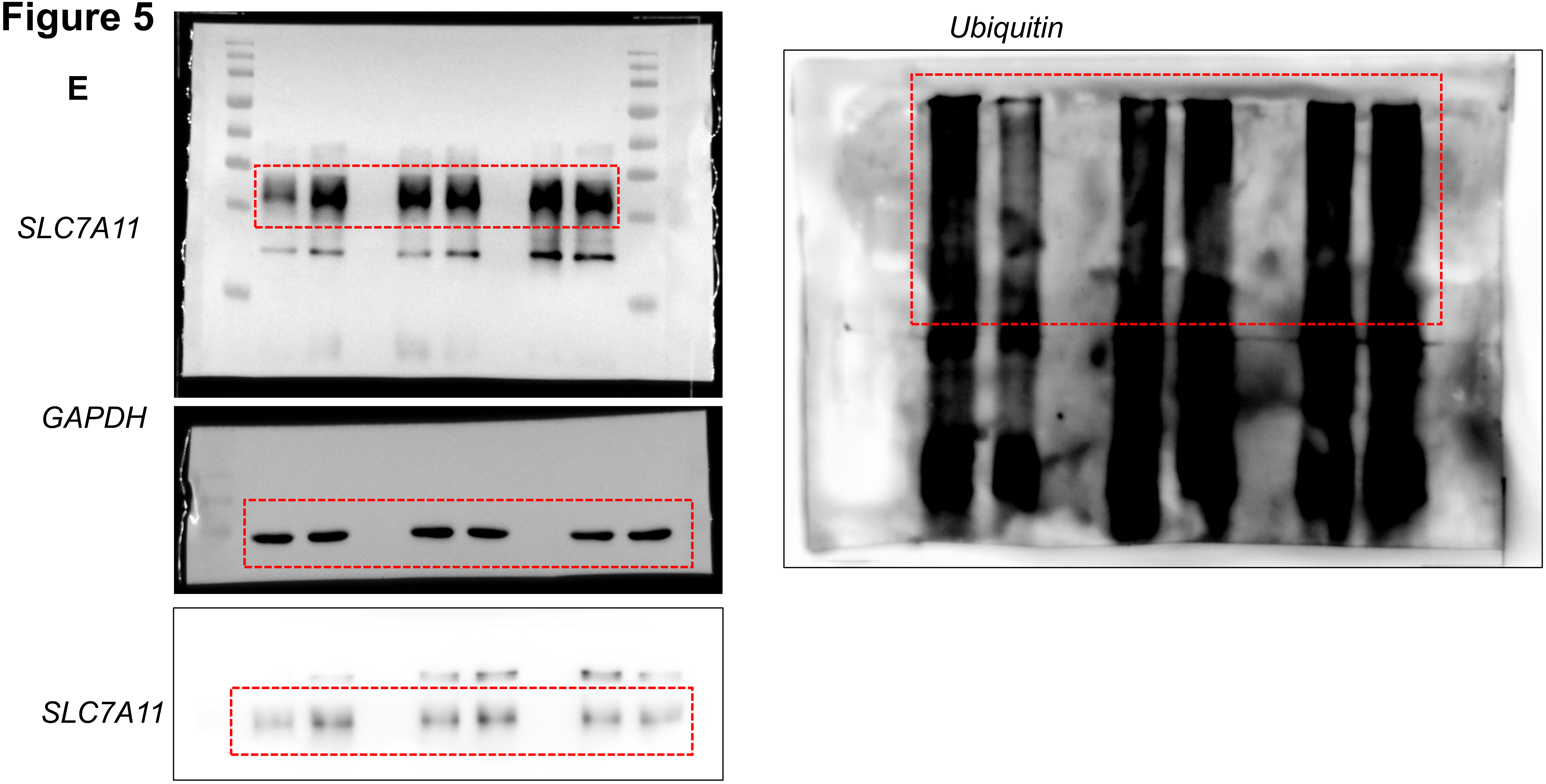

Figure 6

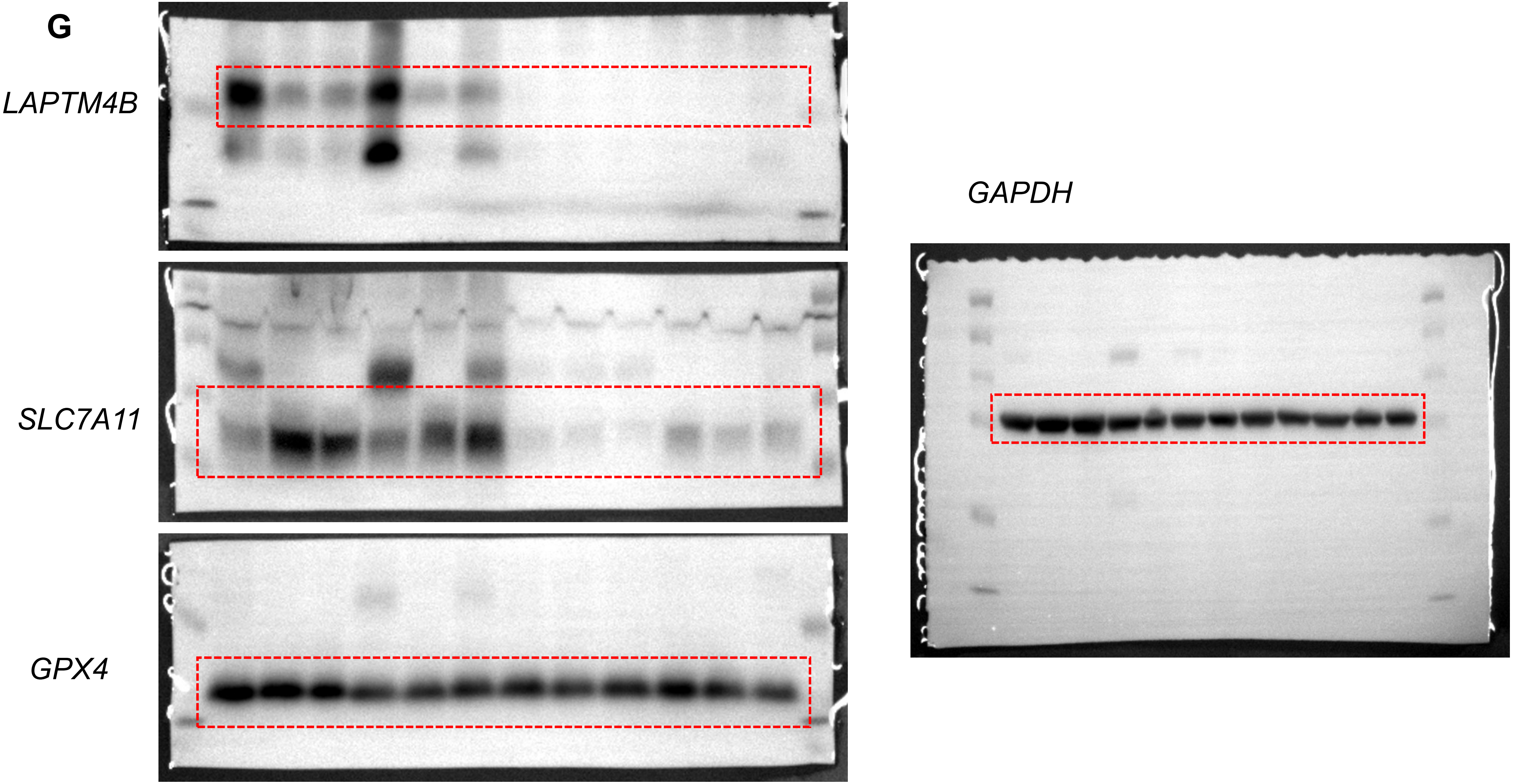

Figure 7

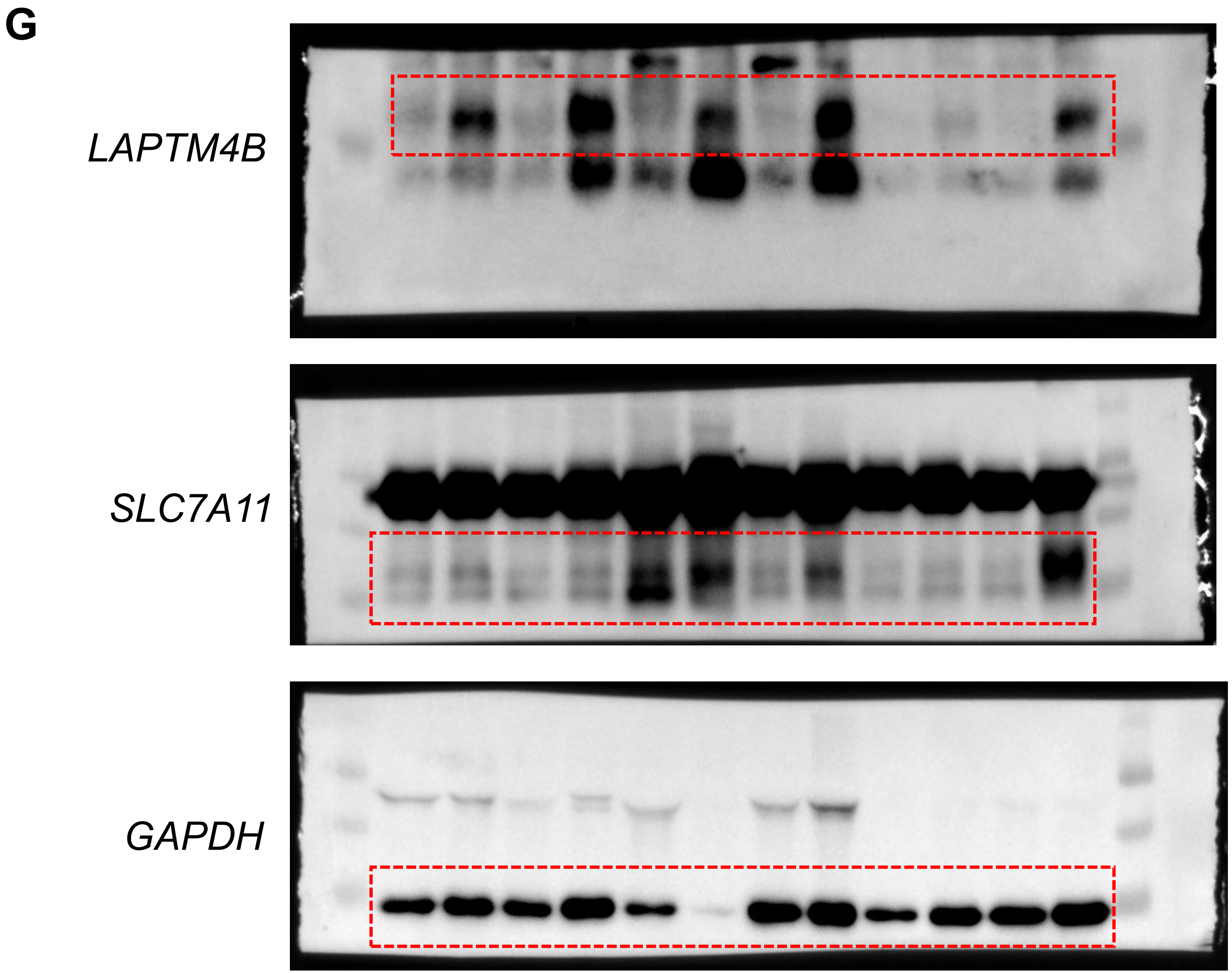

Supplementary Figure S1

A

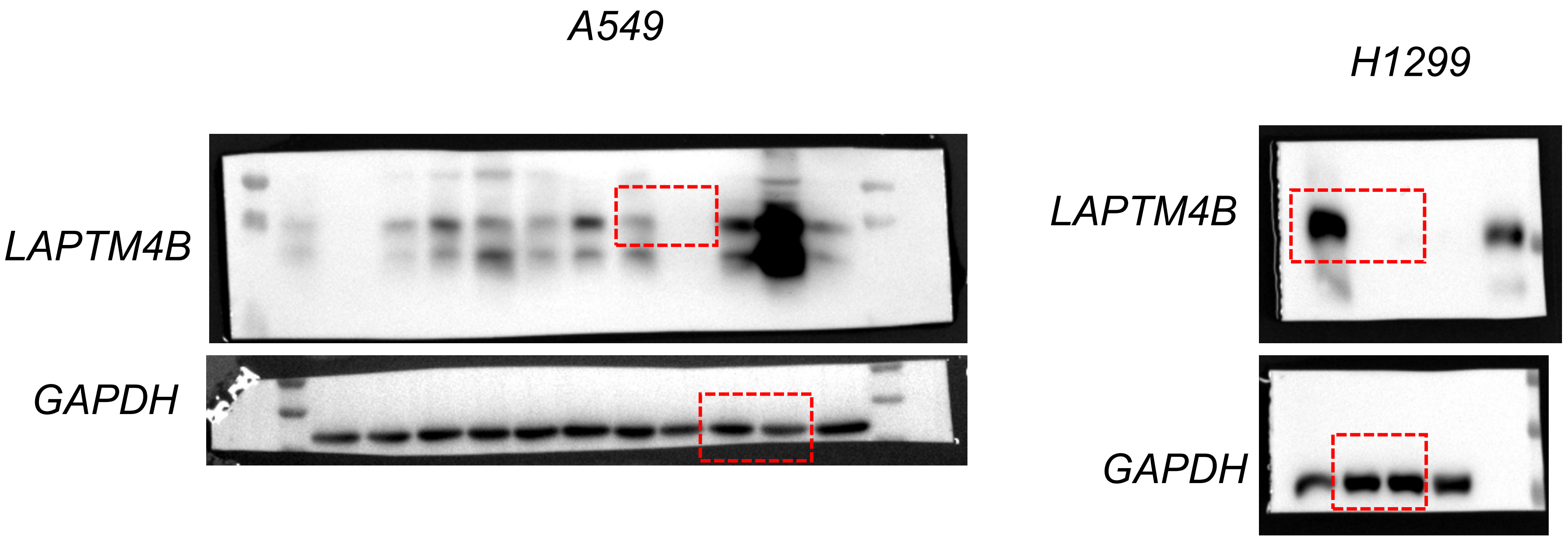

Supplementary Figure S3

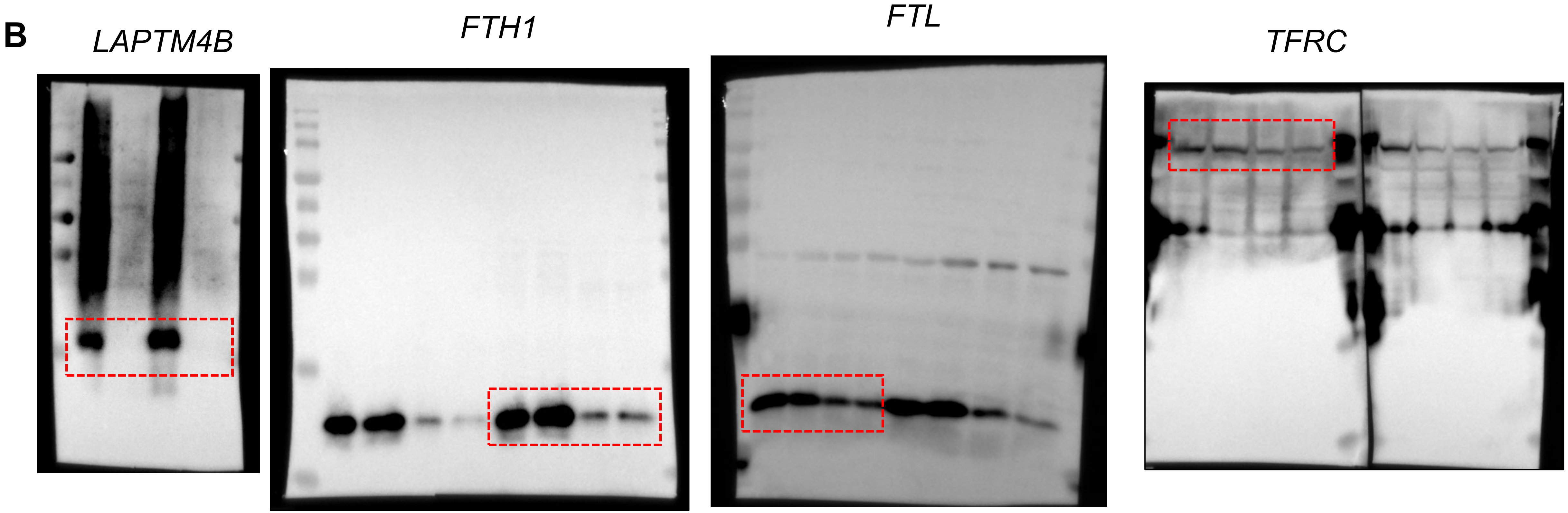

Supplementary Figure S3

B

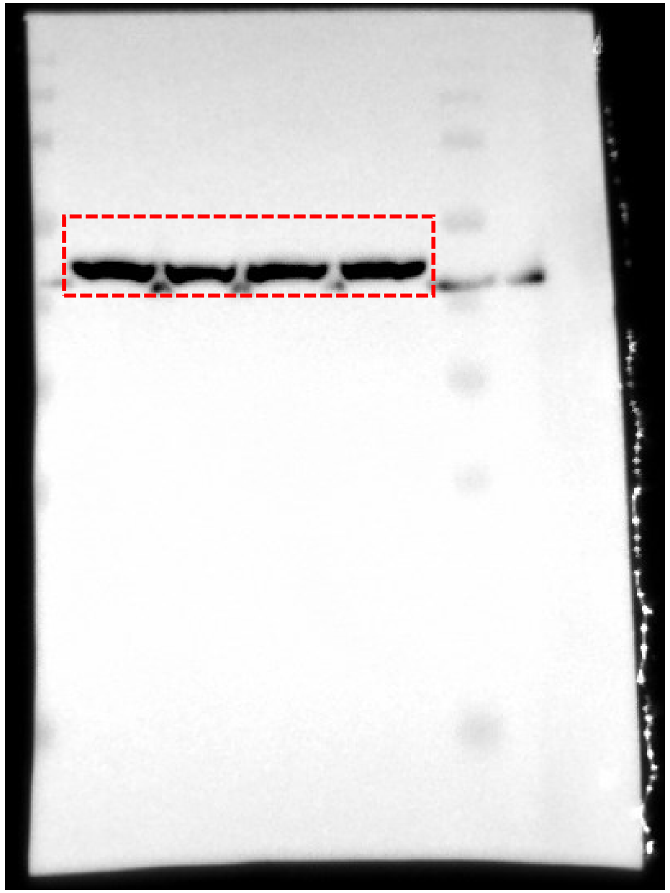

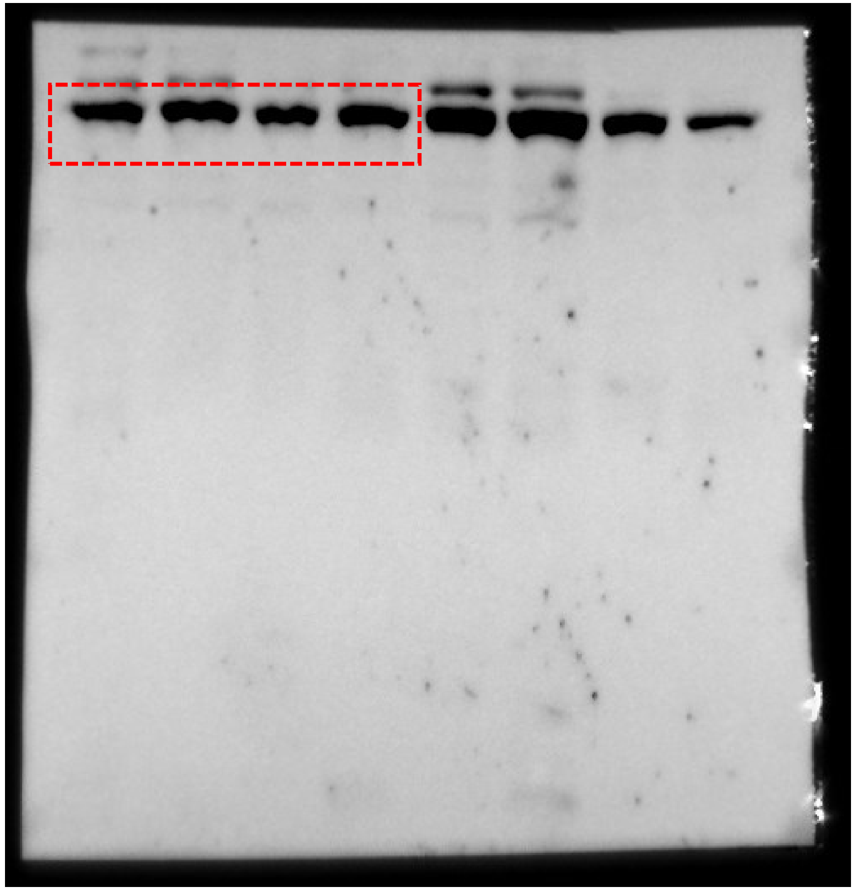

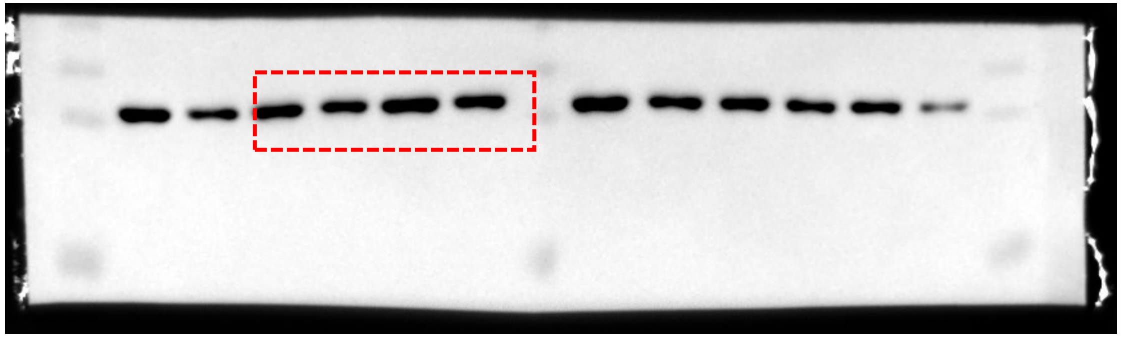

Supplementary Figure S4

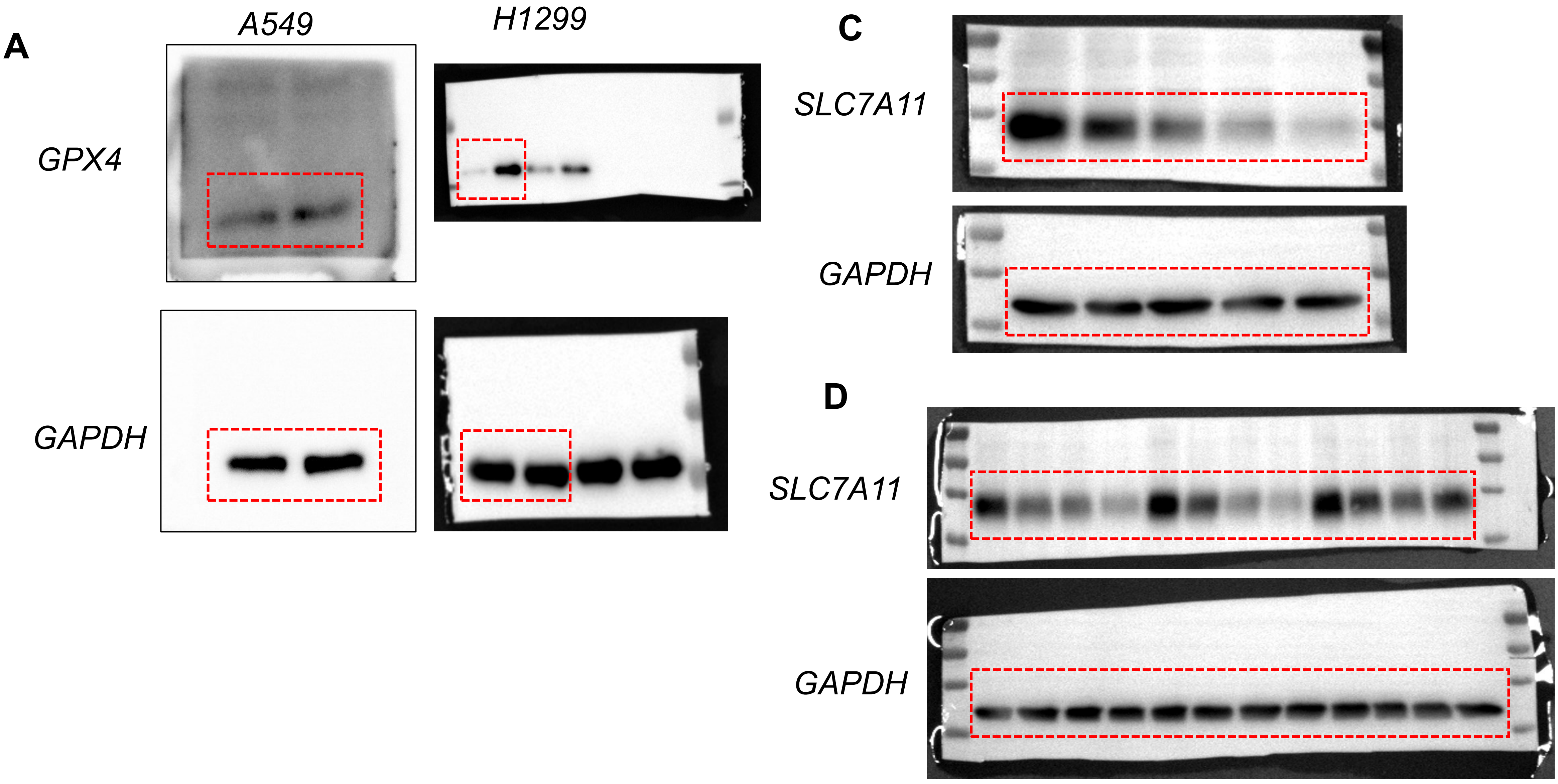

Supplementary Figure S4

E

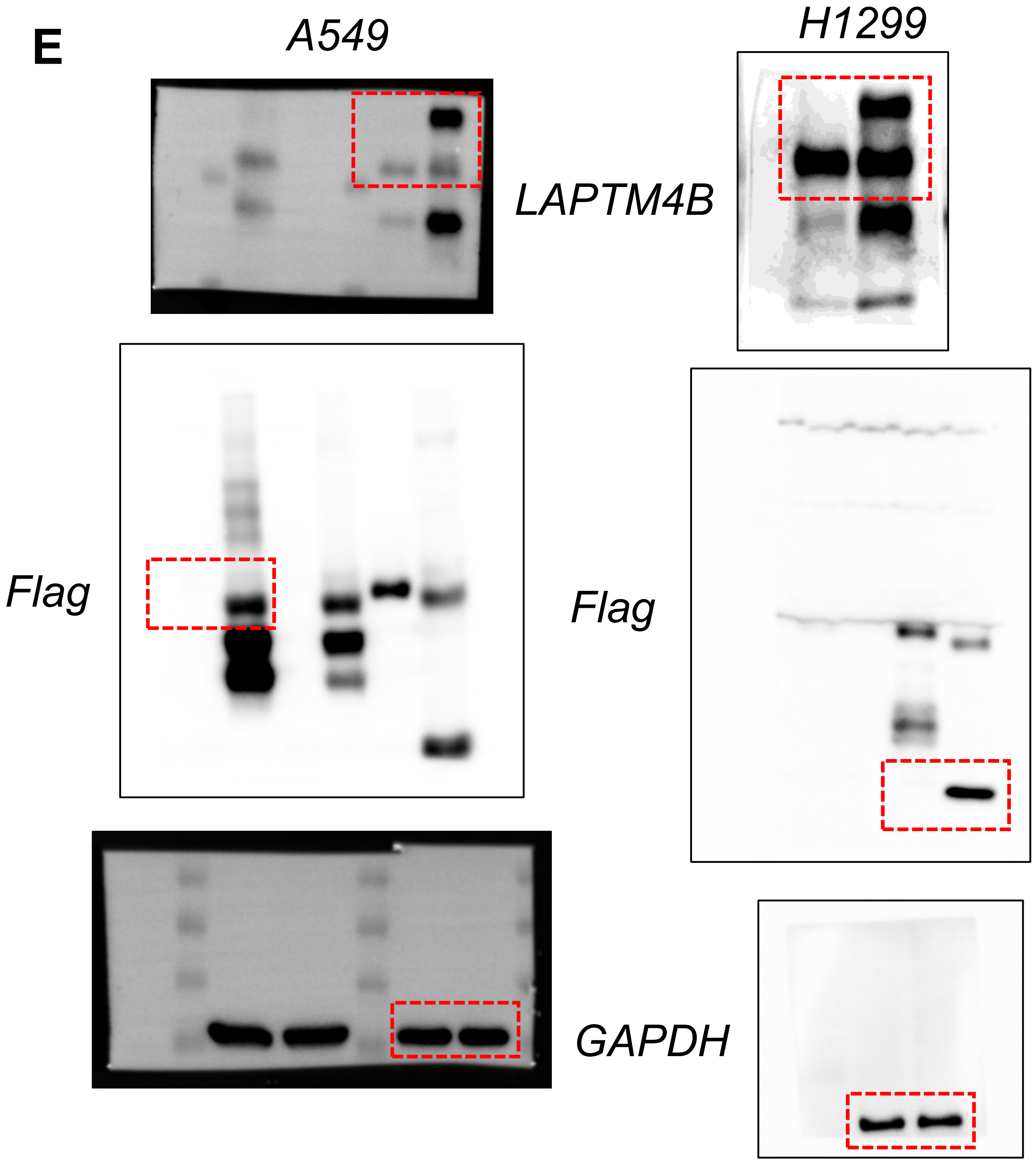

G

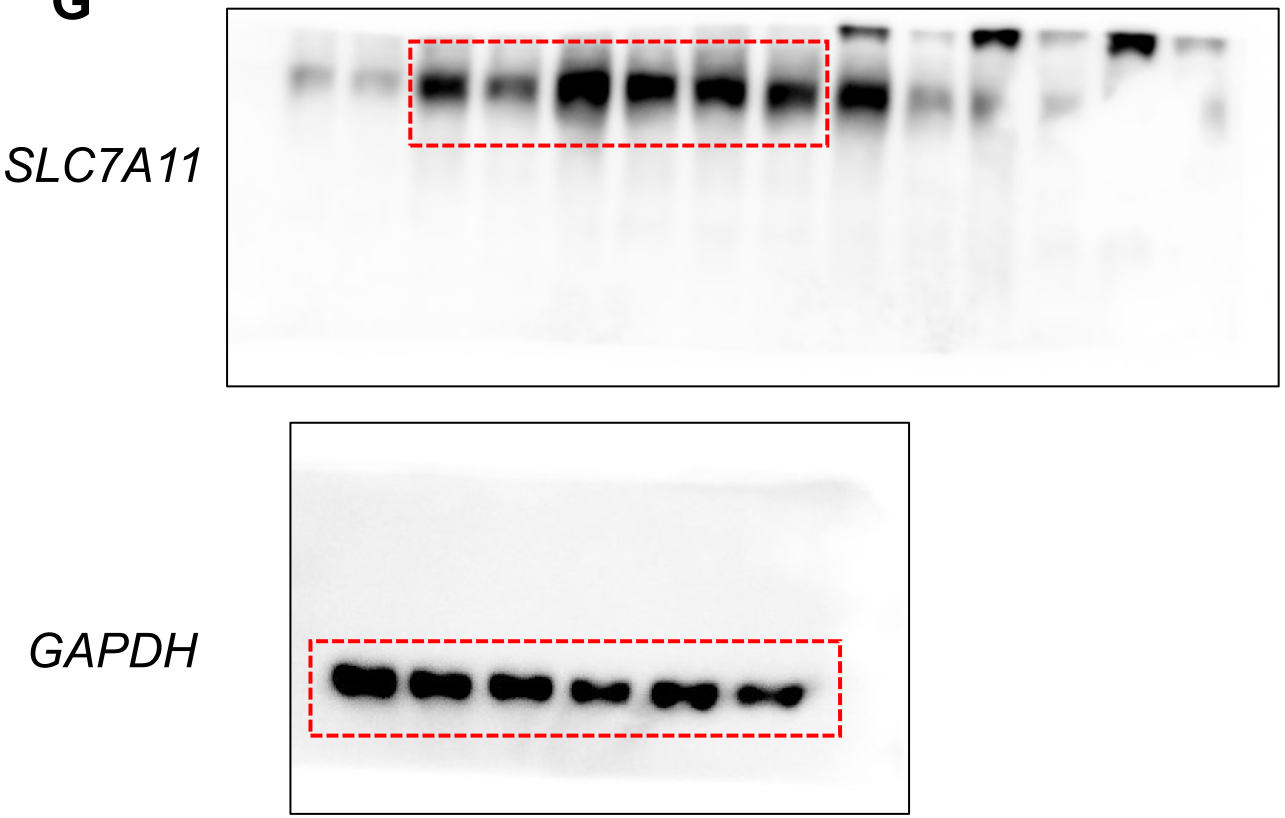

Supplementary Figure S4

H

SLC7A11

GAPDH

SLC7A11

Ubiquitin

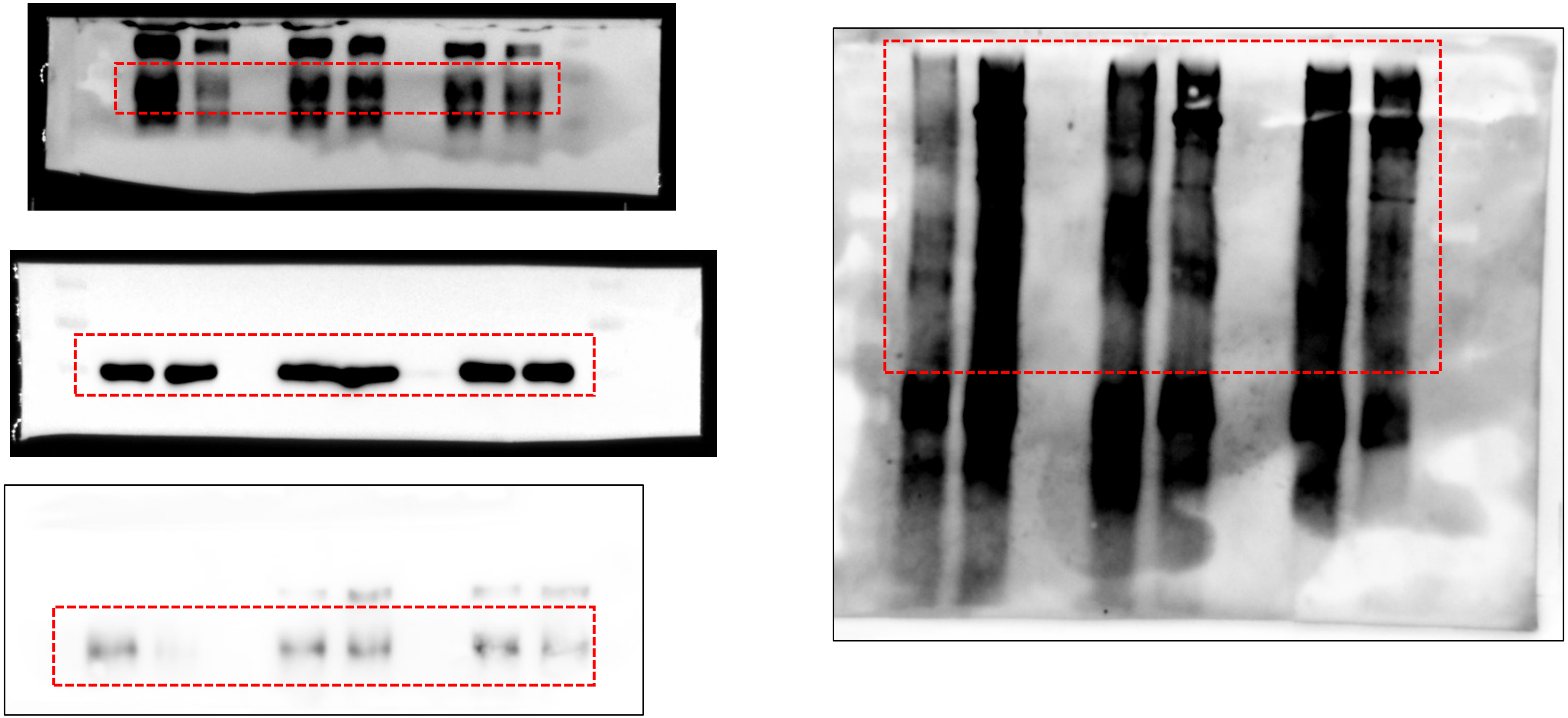

Supplementary Figure S5

D

LC3

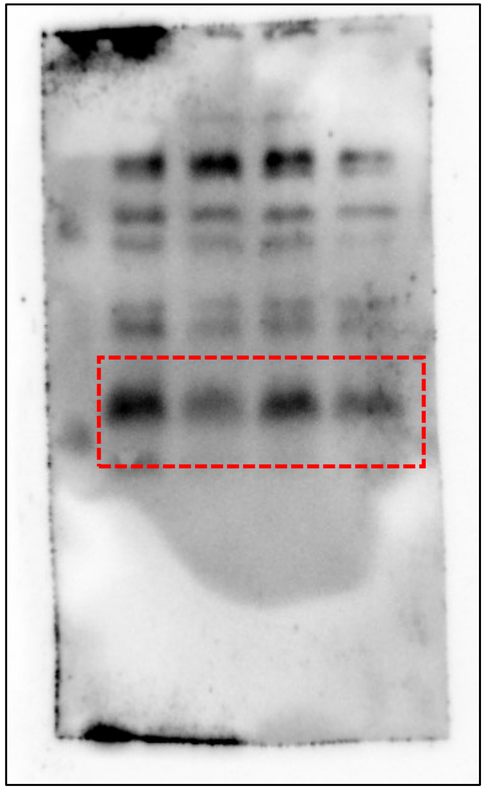

GAPDH

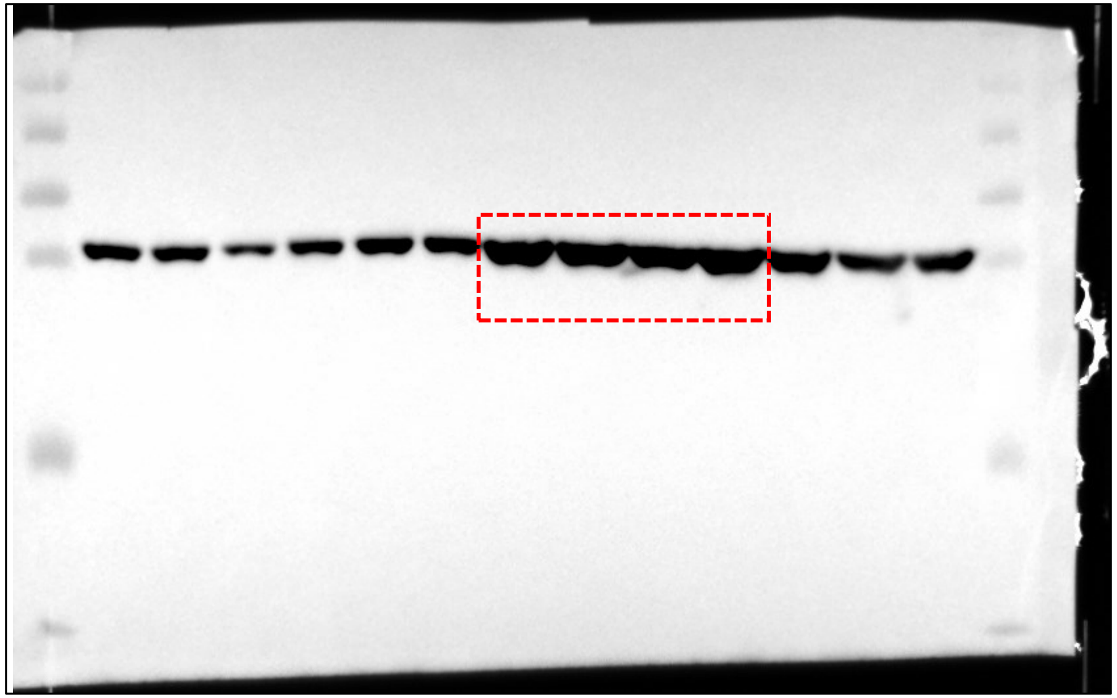

Supplementary Figure S6

A

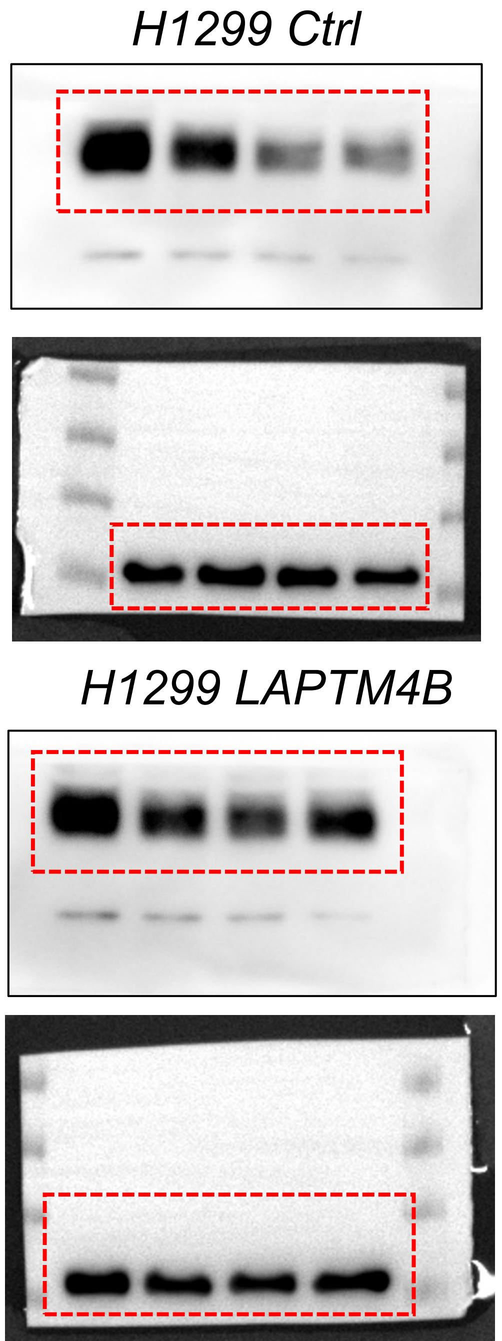

B

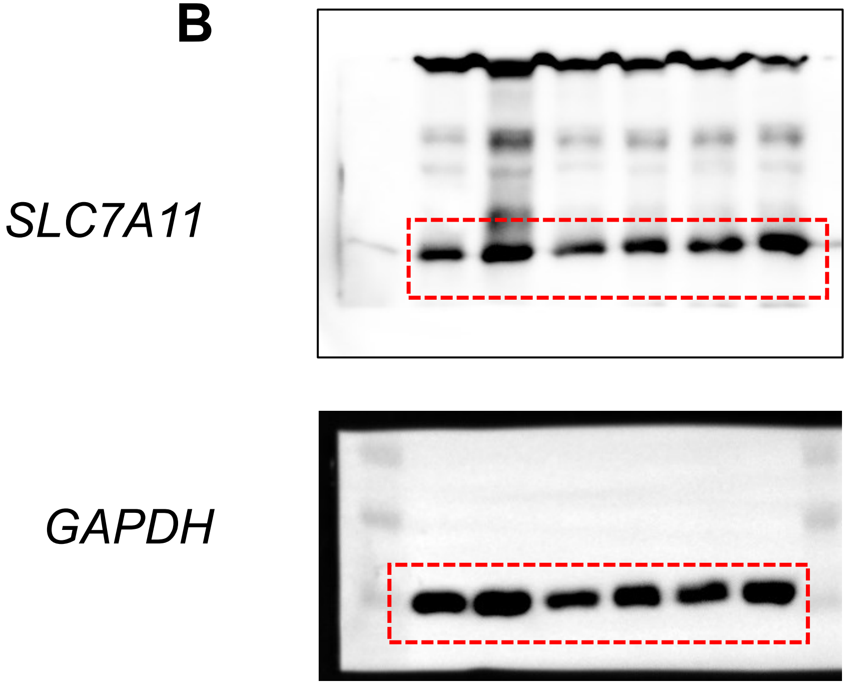

Supplementary Figure S6

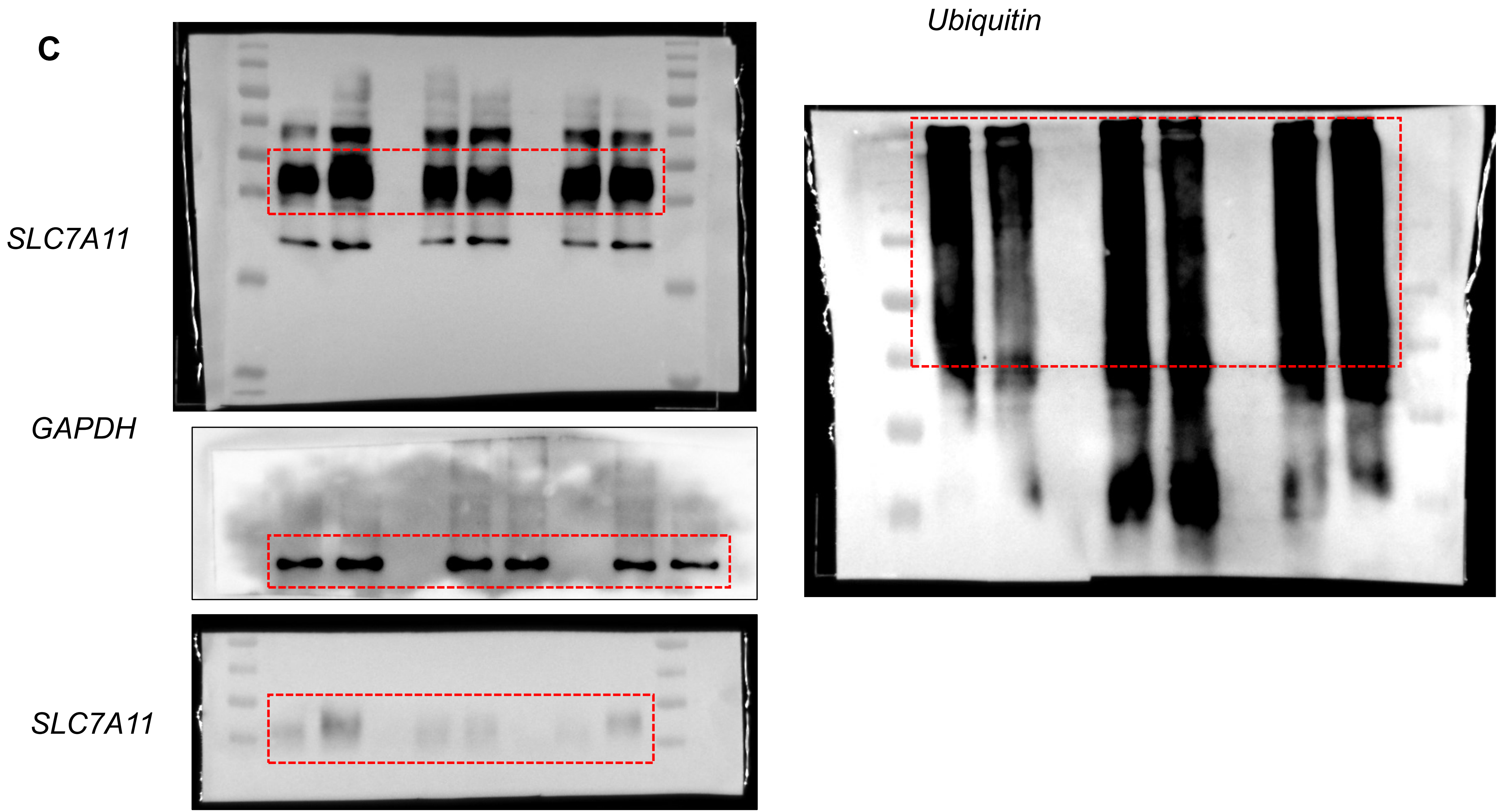

Supplementary Figure S7

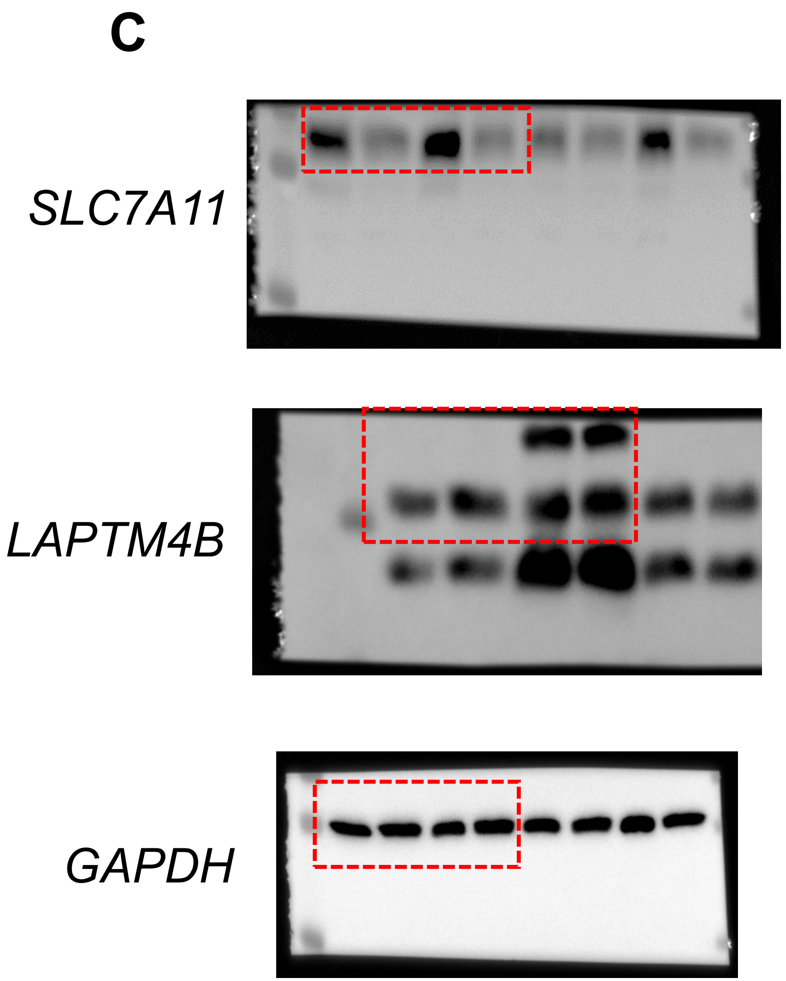

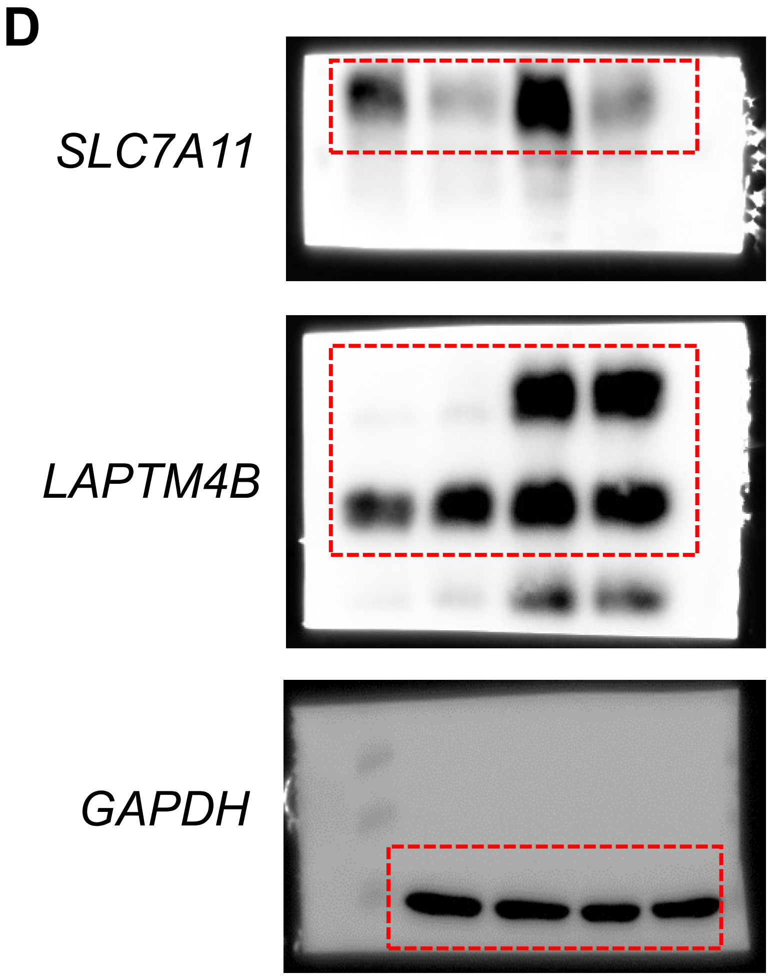

Supplementary Figure S8

B

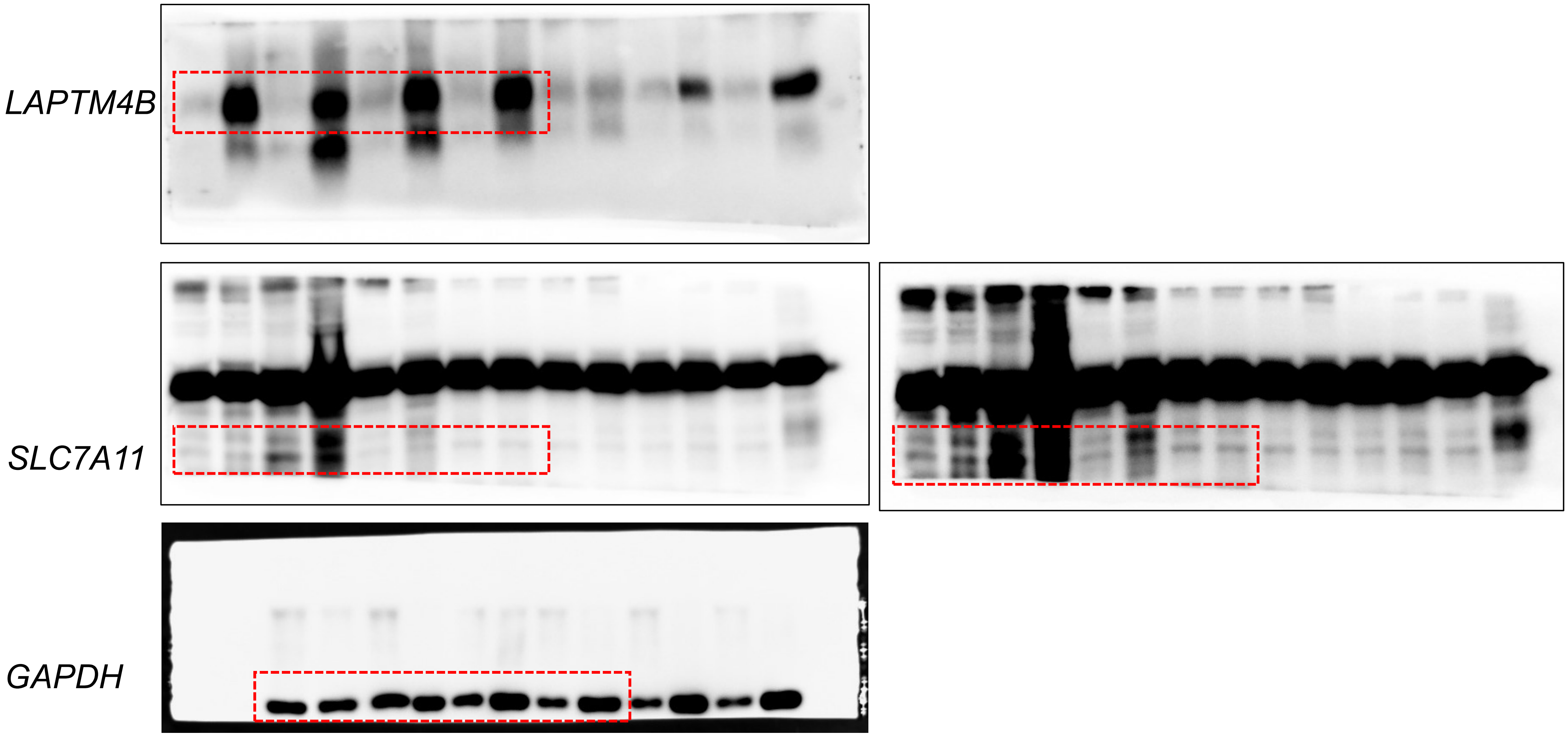

Supplementary Figure S9

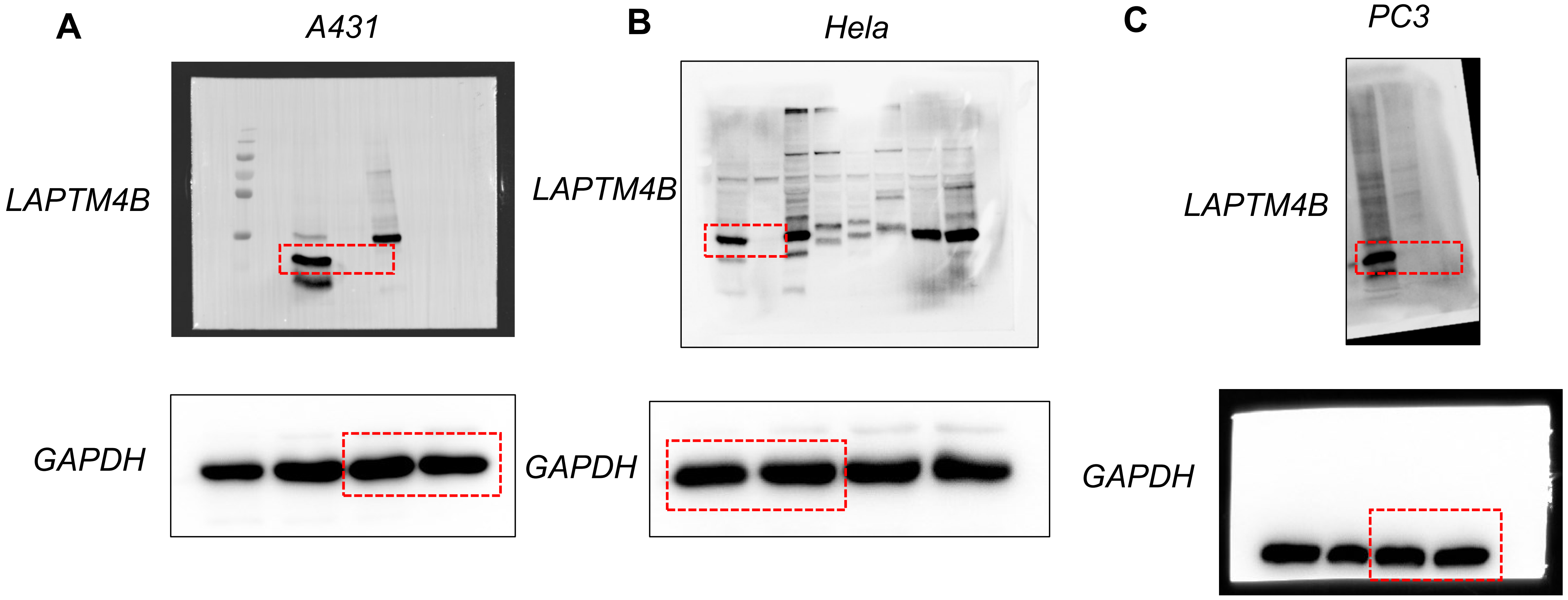

Supplement: Supplementary file 7 — Original Western Blot [file 41419_2024_6836_MOESM7_ESM.pdf]
